# Estimating the scale of chronic hepatitis B virus infection among migrants in EU/EEA countries

**DOI:** 10.1186/s12879-017-2921-8

**Published:** 2018-01-11

**Authors:** Amena A. Ahmad, Abby M. Falla, Erika Duffell, Teymur Noori, Angela Bechini, Ralf Reintjes, Irene K. Veldhuijzen

**Affiliations:** 1 Faculty Life Sciences, Department of Health Sciences, Hamburg University of Applied Sciences, Ulmenliet 20, 21033 Hamburg, Germany; 2 Department of Internal Medicine, University Medical Center Hamburg-Eppendorf, Martinistr. 52, 20246 Hamburg, Germany; 3 Department of Public Health, Erasmus MC, University Medical Center Rotterdam, Wytemaweg 80, 3015 Rotterdam, CN Netherlands; 4 Division of Infectious Disease Control, Municipal Public Health Service Rotterdam-Rijnmond, Schiedamsedijk 95, 3011 Rotterdam, EN Netherlands; 5 European Centre for Disease Prevention and Control, Granits väg 8, 171 65 Solna, Stockholm, Sweden; 6 Department of Health Sciences, University of Florence, Viale G.B. Morgagni, 48, 50134 Florence, Italy; 7 Center for Infectious Disease Control, National Institute for Public Health and the Environment, Antonie van Leeuwenhoeklaan 9, 3721 Bilthoven, MA Netherlands

**Keywords:** Hepatitis B, Chronic viral hepatitis, Migrants, Epidemiology, Europe

## Abstract

**Background:**

Chronic hepatitis B (CHB) related morbidity and mortality can be reduced through risk group screening, linkage to care and anti-viral treatment. This study estimates the number of CHB cases among foreign-born (migrants) in the European Union and European Economic Area (EU/EEA) countries in order to identify the most affected migrant populations.

**Methods:**

The CHB burden was estimated by combining: demographic data on migrant population size by country of birth in the EU/EEA, extracted from European statistical databases; and CHB prevalence in migrants’ countries of birth and in EU/EEA countries, derived from a systematic literature search. The relative contribution of migrants from endemic countries to the total CHB burden in each country was also estimated. The reliability of using country of birth prevalence as a proxy for prevalence among migrants was assessed by comparing it to the prevalence found in studies among migrants in Europe.

**Results:**

An estimated 1–1.9 million CHB-infected migrants from endemic countries (prevalence ≥2%) reside in the EU/EEA. Migrants from endemic countries comprise 10.3% of the total EU/EEA population but account for 25% (15%–35%) of all CHB cases. Migrants born in China and Romania contribute the largest number of infections, with over 100,000 estimated CHB cases each, followed by migrants from Turkey, Albania and Russia, in descending order, with over 50,000 estimated CHB cases each. The CHB prevalence reported in studies among migrants in EU/EEA countries was lower than the country of birth prevalence in 9 of 14 studies.

**Conclusions:**

Migrants from endemic countries are disproportionately affected by CHB; their contribution however varies between EU/EEA countries. Migrant focused screening strategies would be most effective in countries with a high relative contribution of migrants and a low general population prevalence. In countries with a higher general population prevalence and a lower relative contribution of migrants, screening specific birth cohorts may be a more effective use of scarce resources. Quantifying the number of CHB infections among 50 different migrant groups residing in each of the 31 EU/EEA host countries helps to identify the most affected migrant communities who would benefit from targeted screening and linkage to care.

## Background

Migration flows in the first half of the twentieth century were predominantly from Europe towards America. Since the Second World War, economic and geopolitical factors such as decolonisation, labour migration, the collapse of communism, air travel, economic growth and political crisis have changed this and migration to Europe has increased [1]. Much of this migration has been from low- and middle-income countries in Asia and Africa, many of which have a high prevalence of hepatitis B and C [2, 3]. Existing case-based surveillance systems such as the European hepatitis B and C surveillance system managed by the European Centre for Disease Prevention and Control are unable to accurately quantify the number of chronic viral hepatitis cases among migrants on account of different reporting, testing and screening practices among member states. Additional information sources and epidemiologic research are needed to estimate the scale of chronic hepatitis B virus infection in this population [4].

Hepatitis B virus (HBV) infection primarily affects the liver. It usually has an insidious onset and can remain undetected for many years. Up to 5% of HBV infections in adults (up to 90% in young children) can progress to become chronic and up to 30% of chronic cases may develop liver cirrhosis [5].

Public health measures, including antenatal screening, childhood HBV vaccination, stringent testing of blood products, improved infection control practices and harm reduction programmes, have led to a significant reduction of viral hepatitis transmission and a decline in the number of acute HBV cases reported in many European Union/European Economic Area (EU/EEA) countries [6]. Limited or more recent implementation of these primary prevention measures explains the high prevalence of viral hepatitis seen in many parts of the world, but especially in South East Asia, Sub-Saharan Africa and Eastern Europe [7]. Vertical transmission from mother to child and nosocomial transmission are considered to be the main routes in intermediate (2–8%) and high (>8%) HBsAg prevalence countries [5, 7].

Worldwide viral hepatitis related mortality in absolute terms increased by 63% between 1990 and 2013, while the associated disability adjusted life years increased by 34% during this time [8]. This global increase is largely the result of inadequate prevention measures combined with population growth in hepatitis endemic areas [8]. An estimated 13 to 14 million people in the WHO European region are chronically infected with hepatitis B [9, 10] and about 36,000 people die every year as a consequence [9]. In Europe, chronic HBV infection is a major cause of liver cirrhosis and 10–15% of hepatocellular carcinoma (HCC), cases are attributed to chronic hepatitis B (CHB) [10].

Antiviral treatment with nucleot(s)ides such as tenofovir or entecavir can prevent the development of cirrhosis and HCC and can suppress viral replication in a very high proportion of cases [3]. However, because of the largely asymptomatic nature of the infection until late stages, it is estimated that between 40% and 80% of people infected are unaware of their infection and many are not diagnosed until after liver damage has occurred [10, 11]. The population health benefits of effective treatment can only be realised by improving early detection of infection through targeted testing among risk groups.

The WHO recently ratified the strategic goal to eliminate chronic viral hepatitis as a health threat in Europe by 2030. The strategy and action plan published to support countries and the region to achieve this goal highlight ‘the who’ and ‘the where’ as the first two strategic pillars of elimination [12, 13]. Whilst it is suspected that a large proportion of migrants to the EU come from hepatitis B (HBsAg) intermediate (2%–8%) and high (>8%) endemicity countries [2, 3], little is known about the epidemiology of CHB among migrants. Specifically lacking are robust estimates of the number of infections among migrants and knowledge about which groups are most affected. Estimates of which migrant groups are most affected and would therefore benefit most from (linguistically/culturally/specifically) targeted screening programmes, early detection and treatment are required if Europe is to achieve this ambitious elimination goal.

The aims of this study are: 1) to estimate the number of CHB cases among the foreign-born population originating from intermediate and high HBV endemicity countries residing in the 31 countries of the EU/EEA; 2) to estimate the relative contribution of migrants to the overall burden of CHB in Europe; and 3) to identify the migrant groups among whom the largest number of cases are found so as to help direct more effective screening programmes. In a sister paper (INFD-D-17-00468) [14], we conduct a similar analysis for chronic hepatitis C among migrants from endemic countries.

## Methods

**Fig. 1:**
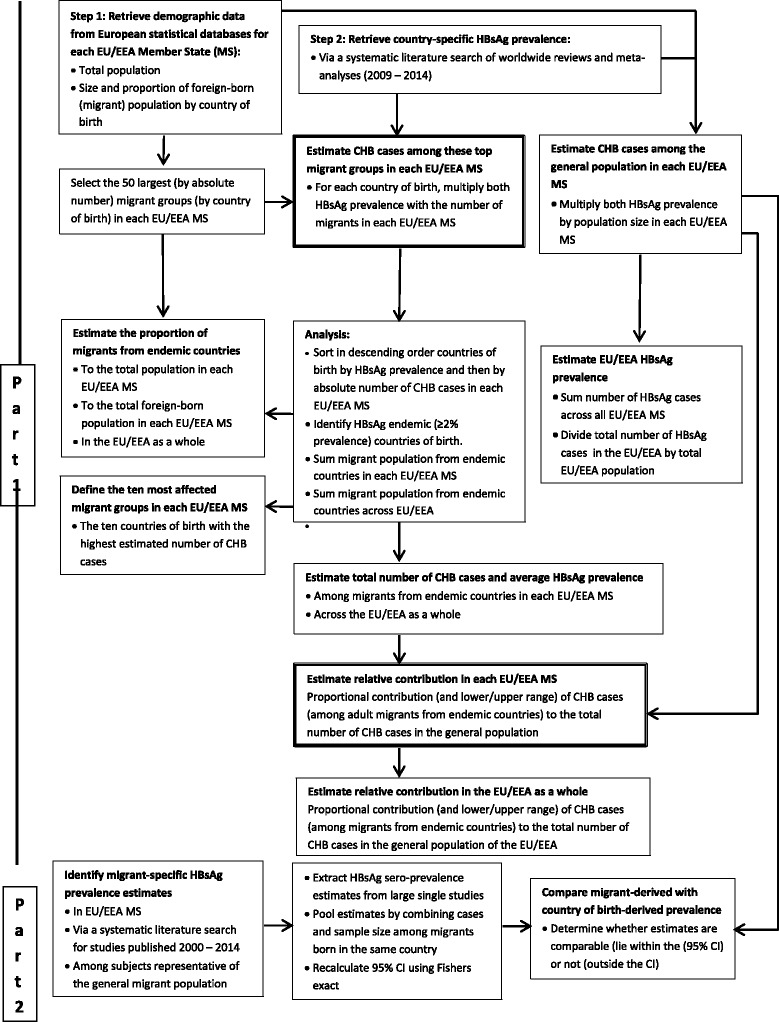
Schematic representation of the methodological process to estimating the burden of chronic hepatitis B among migrants in the EU/EEA

The data retrieval and analysis process are described in detail below and in a schematic representation (Fig. 1). To estimate the number of CHB cases among migrants in each EU/EEA country, demographic data on the number of foreign-born migrants by country of birth living, in EU/EEA countries were extracted from statistical databases. Country of birth-specific and EU/EEA country-specific general population Hepatitis B surface antigen (HBsAg) prevalence estimates were derived from a systematic literature search (Part 1). To assess the reliability of using country of birth-derived HBsAg prevalence as a proxy for the prevalence among migrants, a systematic literature search was conducted to identify prevalence estimates among migrants in Europe and to compare these with country of birth-derived prevalence (Part 2).

### Definitions

Migrant (foreign-born population): includes all persons who were born outside their current country of residence (and listed in the demographic registration databases used in this study). This includes within-EU/EEA migrants, i.e. persons born in another EU/EEA country, as well as those born outside the EU. It does not include undocumented migrants.

Chronic hepatitis B (CHB): refers to a positive hepatitis B surface antigen (HBsAg) test Note: According to the WHO and EU (2012) case definition, detection of HBsAg on two occasions at least 6 months apart is classified as CHB. In this study, as in the seroprevalence and screening studies from which prevalence data for this study were extracted, the presence of HBsAg is taken as the standard proxy for chronic infection. The lack of regular screening for HBV and the reduction in incidence of acute HBV infections in most countries justify the assumption that the overwhelming majority of HBsAg positive cases are chronic.

Hepatitis B endemic country: countries with a ≥ 2% HBsAg prevalence in the general population. This follows from the WHO classification of low (<2%), intermediate (2% to 8%) and high (>8%) endemic countries.

### Part 1: The contribution of migrants from endemic countries to the burden of CHB in the EU/EEA

#### Demographic data

The size and country of birth of the foreign-born migrant population was obtained for the 31 EU/EEA countries from Eurostat for 2013, if available [15]. Where Eurostat data by country of birth were missing (Croatia, Cyprus, France, Germany, Malta, Portugal and the UK), data from the EU 2011 – Housing and Population Census were used [16]. The most recent demographic data for Greece (2012) and Luxembourg (2010) were only available from the Organisation for Economic Co-operation and Development’ (OECD) Stats website [17]. No demographic data were available from the above sources for Lithuania. Data were thus obtained from the Lithuanian National Statistics Service (2013) [18]. The data source is indicated in footnotes in Table 1. For each EU/EEA country, the countries of birth of foreign-born migrants were arranged in descending order of magnitude by the number of migrants. The top 50 countries of birth by size of migrant population were selected for estimating the CHB burden.

#### Systematic literature search to estimate country- specific HBsAg prevalence

The online databases Medline, Embase, the Cochrane library, Web of Science, Scopus, PubMed publisher and Google Scholar were searched in January 2015 for reviews, systematic reviews and meta-analyses in English about the prevalence of hepatitis B in the general population at national level. The search terms (described in full in Annex 1 of the Additional file 1) consisted of a combination of disease-related (hepatitis B), outcome-related (prevalence), population-related (general population, worldwide), and study design-related (reviews) terms. Since the aim was to identify recent reviews, the search was restricted to papers published between 2009 and 2014. The titles and abstracts of retrieved articles were assessed for relevance using exclusion criteria. Key exclusion criteria included studies about hepatitis other than type B; focusing on natural history, clinical features or complications of hepatitis; about medical treatment; focusing on high risk groups e.g. people who inject drugs; and single case studies and cost effectiveness analyses. Full texts of the selected abstracts were retrieved and assessed, decisions to exclude were recorded and a PRISMA flowchart (described in Annex 2 of the Additional file 1) was prepared.

From included reviews, country-specific HBsAg prevalence estimates and confidence intervals (CI) were extracted into a Microsoft Excel database. Where a country-specific estimate was unavailable, the relevant Global Burden of Disease region estimate was used, if available. If a meta-analysis reported a statistically significant time trend, the estimate from the most recent period was selected. When multiple estimates for a country were available from different reviews, the most robust or relevant review was selected based on the following criteria: sampling method; representativeness of population studied; geographical coverage; sample size; quality of included studies and data collection timeframe. Decisions were made jointly by two reviewers (AF and IV) with the rationale recorded for each decision about a chosen estimate. This rationale, together with the search strategy, the inclusion and exclusion criteria and the PRISMA flowchart are described in annexs 1, 2, 3 and 5 of the Additional file 1 to this article.

#### Estimating the number of CHB cases among foreign-born migrants from endemic countries in each EU/EEA country

The retrieved HBsAg general population prevalence estimate in the countries of origin were multiplied by the number of migrants from that respective country in each EU/EEA country. The number of migrants born in endemic countries (≥2%) was summed to determine the total and proportional contribution of migrants from intermediate/high hepatitis B endemicity countries to the overall number of migrants residing in the host country. The ten migrant populations originating from intermediate and high endemicity countries with the highest number of HBsAg infected cases in the EU/EEA were determined.

#### Relative contribution

To estimate the relative contribution of migrants born in endemic countries to the overall number of people infected with CHB in the respective EU host country, the estimated number of infected cases among migrants was divided by the number of infected persons in that country based on the general population prevalence. Given the uncertainty in the size of migrant population and the CHB prevalence estimates in the countries of birth, the range of the relative contribution with a lower and a higher limit was calculated using the Delta method [19].

#### EU/EEA-level estimates

The population of the EU/EEA was derived by summing up the population of all 31 EU/EEA countries as extracted from demographic sources. To estimate the HBsAg prevalence in the EU/EEA, the number of estimated HBsAg positive cases in all 31 EU/EEA countries was summed up and divided by the total EU/EEA population. To derive the lower and upper prevalence range, the lower and upper estimates of the number of cases across the 31 countries were summed up. To estimate the number of cases among migrants to and within the EU/EEA, the number of cases among migrants from endemic countries (≥2%) across all 31 countries was summed up. This was then divided by the number of cases in the EU/EEA to derive the relative contribution of migrants from endemic countries to the burden of CHB infection in the EU/EEA.

### Part 2: Systematic literature search for HBsAg prevalence in migrant populations in Europe

The online databases Medline, Embase, the Cochrane library, Web of Science, Scopus, PubMed publisher and Google Scholar were searched in November 2014 for studies in English that estimate the prevalence of hepatitis B among migrants in any of the 31 EU/EEA countries. The search consisted of a combination of disease-related (hepatitis B), outcome-related (prevalence), population-related (migrants) and geographical area (EU/EEA countries) terms and was limited to studies published between 2000 and 2014. Only studies about the prevalence in migrants who were considered to be representative of the general migrant population (i.e. not refugees or asylum seekers, hospital patients or other higher risk groups and not lower risk groups like pregnant women or children) were compared with in-country of birth derived prevalence estimates. The full search strategy, inclusion and exclusion criteria and PRISMA flowchart can be found in annexs 3, 4 and 6 of the Additional file 1.

Country-level HBsAg prevalence estimates among migrants residing in different European countries were extracted from the included studies and entered into Microsoft Excel. Pooled estimates for countries of birth were produced by combining the numbers tested and the number of cases. A 95% CI was re-calculated using the Fisher’s exact method. Both pooled and large single study (>25 subjects from a single country of birth) estimates were compared with the in-country estimates extracted in Part 1 to determine whether in-country estimates reflect the prevalence found in migrants. When the point estimate from a study in migrants (Part 2) fell within the CI of the in-country estimate (from Part 1), the estimate was considered to be comparable; when it fell below the lower CI limit, it was considered lower than the in-country prevalence; and when it was higher than the upper CI limit the prevalence in migrants was considered to be higher.

## Results

### Estimated CHB prevalence and number of infected cases in 31 EU/EEA countries

Chronic hepatitis B (HBsAg) prevalence differs considerably among EU/EEA countries, ranging from 0.1% in Ireland and the Netherlands to 5.5% in Romania. The average prevalence in the general population of the EU/EEA is estimated at 1.1%, corresponding to an estimated 5.7 million cases (range 4.0 to 7.5 million). These estimates, together with the total number of infected cases, are listed in Table 1. Italy and Romania are the EU/EEA countries with the highest estimated number of CHB cases, both above 1 million.

**Table 1:** Chronic hepatitis B prevalence in the general population of 31 EU/EEA countries and the estimated number of infected cases

Country	Total Population	HBsAg prevalence			Estimated no. of CHB cases		
		%	Low 95% CI	High 95% CI	Central estimate	Lower estimate	Upper estimate
Austria	8,451,149	0.55	0.34	0.71	46,481	28,734	60,003
Belgium	11,161,642	0.7	0.4	1.2	78,131	44,647	133,940
Bulgaria	7,284,552	4.25	2.80	5.70	309,593	203,967	415,219
Croatia	4284,889^a^	1.47	0.84	2.10	62,988	35,993	89,983
Cyprus	840,407^a^	0.9	0.3	2	7564	2521	16,808
Czech Republic	10,516,125	0.70	0.43	0.98	73,613	45,219	103,058
Denmark	5,602,628	0.55	0.34	0.71	30,814	19,049	39,779
Estonia	1,320,174	0.58	0.42	0.74	7657	5545	9769
Finland	5,426,674	0.2	0.1	0.4	10,853	5427	21,707
France	64,932,339^a^	0.68	0.44	1.05	441,540	285,702	681,790
Germany	80,219,695^a^	0.6	0.4	0.8	481,318	320,879	641,758
Greece	11,090,000^b^	2.33	1.54	3.11	258,397	170,786	344,899
Hungary	9,908,798	1.08	0.04	2.11	107,015	3964	209,076
Iceland	321,857	0.55	0.34	0.71	1770	1094	2285
Ireland	4591,087	0.1	0	0.3	4591	0	13,773
Italy	59,685,227	1.89	1.26	2.52	1,128,051	752,034	1,504,068
Latvia	2,023,825	1.39	1.10	1.67	28,131	22,262	33,798
Liechtenstein	36,838	0.55	0.34	0.71	203	125	262
Lithuania	2,971,905 ^d^	2.03	1.37	2.69	60,330	40,715	79,944
Luxembourg	506,953^c^	0.55	0.34	0.71	2788	1724	3599
Malta	417,432	0.55	0.34	0.71	2296	1419	2964
Netherlands	16,779,575	0.1	0	0.2	16,780	0	33,559
Norway	5,049,223	0.55	0.34	0.71	27,771	17,167	35,849
Poland	38,533,299	1.44	1.16	1.72	554,880	446,986	662,773
Portugal	10,562,178^a^	1.35	0.66	2.04	142,589	69,710	215,468
Romania	20,020,074	5.49	5.24	5.73	1,099,102	1,049,052	1,147,150
Slovakia	5,410,836	0.70	0.43	0.98	37,876	23,267	53,026
Slovenia	2,058,821	3.29	2.33	4.24	67,735	47,971	87,294
Spain	46,727,890	0.66	0.34	0.97	308,404	158,875	453,261
Sweden	9,555,893	0.2	0.1	0.4	19,112	9556	38,224
United Kingdom	63,182,180^a^	0.54	0.30	0.60	341,184	189,547	379,093
EU/EEA^e^	509,474,165	1.12	0.79^e^	1.47^e^	5,705,260	4,003,937	7,514,179

^*^Source is EUROSTAT 2013 unless indicated by the following symbol:

^a^ESS 2011 Census

^b^OECD 2012

^c^OECDC 2010

^d^http:///www.euras.lt (Lithuanian National Statistics Agency

^e^For the 31 EU/EEA countries the cumulative HBsAg prevalence and the upper and lower CI were estimated from the sum of the estimated number of CHB cases (central, lower and upper estimate), hence these should be considered as upper and lower prevalence ranges and not CI

### The distribution of migrants in the EU/EEA based on HBV endemicity in country of birth

**Fig. 2:**
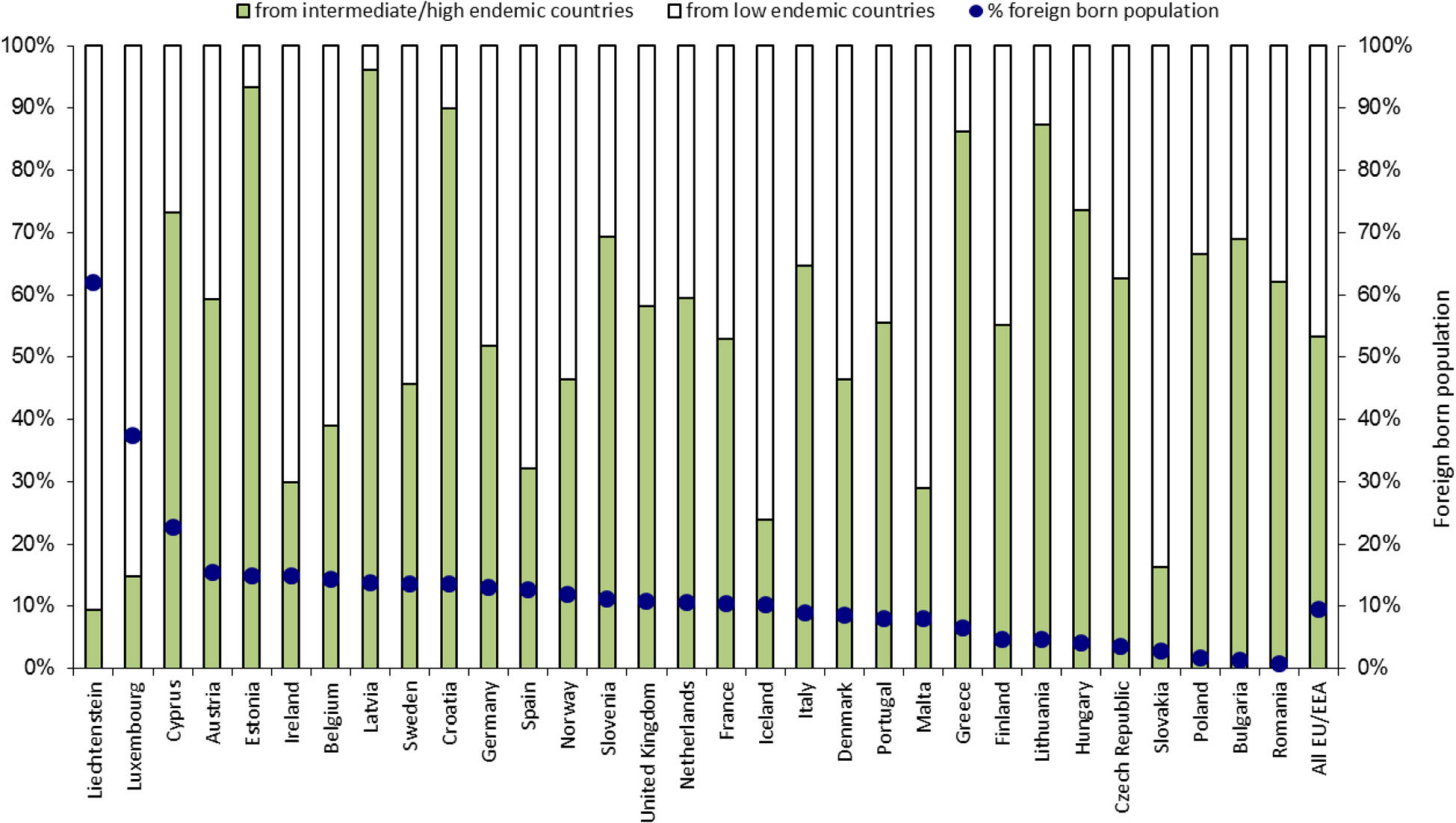
Total (%) of foreign born population (blue dots) in each EU/EEA country and of those the proportion originating from HBsAg endemic countries (≥2%)

The top 50 foreign-born populations in each EU/EEA country included in our analysis make up at least 95% of the total migrant population in 19 of 31 EU/EEA countries and at least 90% in all but three EU/EEA countries (Denmark, Sweden and the UK where it is at least 85%). These migrant populations account for approximately 9.5% of the population in the EU/EEA. The proportion, however, ranges from 0.9% in Romania and 1.3% in Bulgaria to more than 40% in Luxembourg and 62% in Liechtenstein (Fig. 2). Just over half of the EU/EEA migrant population were born in HBV endemic (≥2% prevalence) countries. EU/EEA countries with the highest proportion of migrants from endemic countries among their foreign-born population are Croatia, Estonia and Latvia (>90%), and those with the lowest proportion are Liechtenstein, Luxembourg and Slovakia (<16%) (Fig. 2). The foreign-born population and the number and proportion from endemic countries in the EU/EEA and by country are shown in Table 2.

**Table 2:** Foreign-born population and proportion of foreign-born population (from endemic (HBsAg ≥2%) countries), residing in the 31 EU/EEA host countries and the estimated number and range of CHB cases among migrants in these countries as well as the estimated relative contribution to the total number of cases in the EU/EEA host country

Country	Population of foreign-born migrants			Foreign-born migrants from endemic countries				
	Number from top 50 countries	Number from HBV endemic countries	Proportion from HBV endemic countries	Estimated number of CHB cases			Average CHB prevalence	Estimated relative contribution (and range) of CHB cases among migrants to the total number of CHB cases in the host country
				Central estimate	Lower estimate	Upper estimate		
Austria	1,298,945	768,773	9.1%	33,456	25,757	41,040	4.4%	72% (43% - >100%^a^)
Belgium	1,596,848	622,206	5.6%	42,530	32,218	54,309	6.8%	54% (20% - 89%)
Bulgaria	90,990	62,755	0.9%	2436	1860	3039	3.9%	1% (0% - 1%)
Croatia	582,271	523,470	12.2%	18,673	11,966	25,376	3.6%	30% (13% - 46%)
Cyprus	190,568	139,689	16.6%	6770	5141	8445	4.8%	90% (2% - >100%^a^)
Czech Republic	374,296	234,291	2.2%	12,185	9637	14,752	5.2%	17% (9% - 24%)
Denmark	484,139	224,384	4.0%	12,352	9605	15,152	5.5%	40% (24% - 56%)
Estonia	197,744	184,642	14.0%	5432	3822	7038	2.9%	71% (42% - 100%)
Finland	257,044	141,953	2.6%	8136	6206	10,067	5.7%	75% (16% - >100%^a^)
France	6,775,948	3,591,002	5.5%	212,538	131,238	380,923	5.9%	48% (13% - 84%)
Germany	10,426,860	5,398,700	6.7%	234,792	180,867	288,066	4.3%	49% (29% - 68%)
Greece	713,471	615,986	5.6%	43,163	36,636	49,346	7.0%	17% (11% - 23%)
Hungary	411,403	302,781	3.1%	15,286	13,649	16,940	5.0%	14% (1% - 28%)
Iceland	32,910	7857	2.4%	421	349	494	5.4%	24% (15% - 33%)
Ireland	687,462	205,071	4.5%	13,196	10,935	15,574	6.4%	>100% (<0% - >100%^a^)
Italy	5,319,754	3,443,409	5.8%	213,063	174,632	251,539	6.2%	19% (12% - 26%)
Latvia	278,243	267,617	13.2%	7866	5269	10,454	2.9%	28% (17% - 39%)
Liechtenstein	22,806	2140	5.8%	97	74	119	4.5%	48% (28% - 67%)
Lithuania	139,712	121,992	4.1%	3765	2469	5057	3.1%	6% (3% - 9%)
Luxembourg	189,858	28,085	5.5%	1450	913	2019	5.2%	52% (26% - 78%)
Malta	33,301	9629	2.3%	637	429	860	6.6%	28% (15% - 41%)
Netherlands	1,772,756	1,052,695	6.3%	56,650	40,335	73,016	5.4%	>100% (<0% - >100%^a^)
Norway	597,316	277,047	5.5%	17,021	12,125	21,979	6.1%	61% (34% - 88%)
Poland	659,657	438,446	1.1%	11,679	7018	16,342	2.7%	2% (1% - 3%)
Portugal	854,830	475,155	4.5%	42,688	29,595	55,795	9.0%	30% (12% - 48%)
Romania	166,973	103,740	0.5%	7531	5453	9581	7.3%	1% (0% - 1%)
Slovakia	155,346	25,170	0.5%	1073	846	1301	4.3%	3% (2% - 4%)
Slovenia	231,276	160,220	7.8%	5713	3756	7663	3.6%	8% (5% - 12%)
Spain	5,930,170	1,909,343	4.1%	118,316	92,282	148,318	6.2%	38% (18% - 59%)
Sweden	1,304,130	596,303	6.2%	33,850	23,728	44,011	5.7%	>100% (34% - >100%^a^)
United Kingdom	6,845,805	3,976,870	6.3%	244,409	195,342	294,417	6.1%	72% (47% - 96%)
EU/EEA	48,622,832	25.911.421	5.1%	1,427,174	1,074,152	1,873,032	5.5%	25% (15% - 35%)

^a^The Delta method does not give a reliable confidence interval around the relative contribution as the relative contribution is close to 100% and the distribution of cases in the general population is extremely skewed

### Country-specific HBsAg prevalence estimates

The most comprehensive systematic global review of country-specific prevalence in the general population identified by the systematic literature search was a review by Kowdley et al. published in 2012 [20]. This provided CHB prevalence estimates for 102 countries, based on studies published between 1980 and July 2010 using population-based surveys and studies of groups considered representative of the general population, such as pregnant women, school children, military recruits and healthy controls from cohort studies. Studies in emigrants to the United States, Europe, Australia and elsewhere were also included. Studies in blood donors and in higher risk populations were excluded. The Kowdley et al. review used meta-analytic methods to estimate country- and region-specific pooled HBsAg seroprevalence and corresponding 95% CI [20].

Since the Kowdley review did not include a prevalence estimate for the United States, this was taken from the most recent nationally representative survey in 2011 [21]. For 11 countries (China, Egypt, Ethiopia, Greece, Italy, Romania, Saudi Arabia, South Korea, Spain, Thailand and Turkey), Kowdley [20] reported a statistically significant decrease in prevalence over time and therefore the post-2000 estimate was taken. Estimates from other studies were considered more robust or relevant than this review for 11 countries (Albania, Algeria, Belgium, Cyprus, Finland, Ireland, Libya, Morocco, the Netherlands, Sweden and Tunisia) [9, 11, 22], and the reasons for this are listed in annex 7 of the Additional file 1. For the 44 countries and territories where no country estimate was available, the relevant regional estimate calculated by Kowdley was used. All country-specific prevalence estimates used can be seen in annex 8 of the Additional file 1, to this article.

### Estimated prevalence and number of CHB infections among migrants

In the EU/EEA overall, between 1 million and 1.9 million migrants born in endemic countries are estimated to have CHB infection, which corresponds to an estimated prevalence of 5.5%. The estimated cumulative number and range of CHB cases among the top 50 migrant populations from intermediate and high endemicity countries in each EU/EEA country is listed in Table 2. The average HBsAg prevalence among migrants from intermediate and high endemicity countries is also available for each EU/EEA country, and ranges from 3% in Estonia, Latvia, Lithuania and Poland to 9% in Portugal.

Migrants originating from China and Romania contribute the largest number of infections, with over 100,000 CHB cases each, followed by migrants from Turkey, Albania and Russia, in descending order, with over 50,000 CHB cases each. Table 3 lists the ten migrant populations with the highest estimated number of CHB cases, adding up to over 680,000 cases and corresponding to 48% of CHB cases among migrants from endemic countries in the EU/EEA. Table 3 lists the EU host countries with the largest populations of migrants born in these countries. Estimates for the 50 migrant groups in each EU/EEA country can be found in annex 9 of the Additional file 1 to this article.

**Table 3:** The ten migrant groups (from HBsAg endemic countries) with the highest estimated number of CHB cases (rounded) and the main host EU/EEA countries

Migrant country of origin	Total migrant population in Europe	HBsAg prevalence	Cumulative number of CHB cases	Host countries (first 6 with largest populations)^a^
Romania	2,817,458	5.5	154,679	Italy, Spain, Germany, Hungary, UK, Austria
China	1,012,550	10.2	103,585	UK, Italy, Spain, France, Germany, Netherlands
Turkey	2,266,977	4.3	97,255	Germany, France, Netherlands, Austria, Belgium, UK
Albania	804,570	9.0	72,412	Italy, Greece, Belgium, Austria, Bulgaria
Russia	1,810,197	2.9	52,315	Germany, Latvia, Estonia, Italy, Spain, Lithuania
Vietnam	365,048	12.5	45,557	France, Germany, Czech Republic, UK, Sweden, Norway
Nigeria	336,155	13.3	44,741	UK, Italy, Spain, Ireland, Netherlands, Austria
Kazakhstan	828,526	5.0	41,013	Germany, Latvia, Czech Republic, Poland, Lithuania, Estonia
Algeria	1,482,465	2.6	38,544	France, Spain, Belgium, Italy, Ireland
India	1,120,352	3.2	36.188	UK, Italy, Germany, France, Spain, Ireland
Total			686,289^b^	

^a^if migrant population is at least 1000

^b^The sum of CHB cases among the ten migrant groups with the largest number of CHB cases (686,289) corresponds to 48% of the total number of CHB cases among migrants from endemic countries (1,427,174)

Migrants from China, Romania and Russia are among the top ten migrant populations with the highest estimated number of CHB cases in 28, 19 and 18 respectively of the 31 EU/EEA countries as listed in  Table 4.

**Table 4:** Countries of birth of foreign-born migrants found amongst the ten migrant groups most affected by chronic hepatitis B in 10 or more of the 31 EU/EEA countries^a^

Country of birth of migrants	Number of EU/EEA countries (of 31)	EU/EEA Countries
China	28	AUT, BEL, BLG, HR, CZ, DK, DE, FIN, FR, EE, HU, IRL, ISL, IT, LIE, LT, LUX, MT, NL, NO, PL, PT, RO, SK, SI, ES, SE, UK
Romania	19	AUT, BEL, BLG, HR, CY, CZ, DK, DE, GRC, HU, IRL, ISL, IT, LUX, MT, PL, PT, SK, ES,
Russia	18	AT, BLG, HR, CY, CZ, DE, FIN, EE, GRC, HU, ISL, LT, LV, MT, PL, RO, SK, SI
Ukraine	14	BLG, HR, CZ, DE, EE, HU, IT, LT, LV, PL, PT, RO, SK, SI
Vietnam	14	BLG, CY, CZ, DK, DE, FIN, FR, HU, ISL, NL, NO, PL, SK, SE
Turkey	12	AUT, BEL, BLG, DK, DE, FIN, FR, GRC, LIE, NL, RO, SE
Moldova	11	BLG, CY, CZ, EE, IRL, IT, LT, LV, PT, RO, SI
Philippines	11	AUT, CY, DK, GRC, IRL, ISL, IT, MT, NO, ES, UK
Afghanistan	10	AUT, BEL, DK, DE, FIN, HU, NL, NO, SK, SE
Bosnia and Herzegovina	10	AUT, HR, DK, DE, LIE, LUX, NO, PL, SI, SE

^a^selected from the ten largest CHB affected migrant groups from intermediate/high endemicity countries in the EU/EEA countries

At least three of the ten migrant populations most affected by CHB in the Czech Republic, Denmark, Finland, Hungary, Iceland, Liechtenstein, Norway, the Netherlands and Sweden were born in South-East or East Asian countries including China, Vietnam, the Philippines and Thailand. People born in Yugoslavia before 1992 or in one of the former Yugoslav Republics since 1992 are represented among three or more of the top ten migrant populations with the highest number of infected cases in Austria, Liechtenstein and Luxembourg as well as in Croatia and Slovenia. Similarly, people born in the Soviet Union before 1991 or in one of the former Soviet Republics since 1991 are represented among three or more of the top ten migrant populations most affected by CHB in Bulgaria, Cyprus, the Czech Republic, Germany, Hungary, Poland, Romania and Slovenia as well as in the Baltic states of Estonia, Latvia and Lithuania (see annex 9 of the Additional file 1).

In the UK, migrants from India, Pakistan and Bangladesh are among the top ten migrant populations with the highest number of infected cases. Migrants from Maghreb countries such as Algeria and Tunisia are represented in the top ten in France. Across the EU/EEA, African countries of origin that contribute a large number of estimated cases include Eritrea, Ghana, Nigeria, Senegal, Somalia and South Africa. In Belgium, France, Luxembourg, Malta, Portugal and the UK, four or more of the ten migrant populations most affected by CHB are from African countries.

### Relative contribution of migrants from endemic countries to the overall CHB burden in EU/EEA countries

**Fig. 3:**
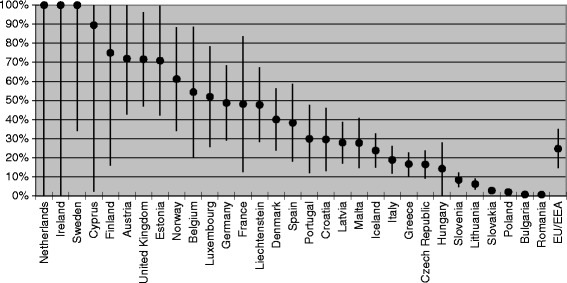
Relative contribution of migrants to the total number of CHB cases per EU/EEA country

The relative proportion of infected migrants from endemic countries among the overall number of CHB cases in EU/EEA countries is shown in Table 2 and Fig. 3. Migrants from intermediate and high endemicity countries contribute more than 90% and, in some instances, up to 100% of the total estimated number of CHB cases in Cyprus, Ireland, the Netherlands and Sweden. Conversely, in Bulgaria, Poland, Romania and Slovakia, migrants contribute less than 4% of the total.

### Comparing migrant-derived HBsAg prevalence with country of origin estimates

Sixteen HBsAg prevalence studies in migrants residing in Europe were identified from the literature search for comparison with the in-country estimates derived in Part 1 (Table 5). Prevalence figures for migrants from Suriname and the Dutch Antilles could only be compared with a regional estimate, since in-country data were not available from the review studies in Part 1.

**Table 5:** HBsAg prevalence derived from studies among general migrant populations resident in Europe compared to in-country prevalence estimates derived from worldwide systematic reviews

Country	Migrants				In-country of origin			Comparison
	N tested	Prevalence	95% CI	Reference	Prevalence	95% CI	Reference	
Afghanistan	293	2.1	0.8–4.4	[30]	10.5	5.9–15.1	[20]	Lower
Albania	504^a^	11.7	9.0–14.8	[31]	9.0	8.1–9.8	[9]	Higher
Bangladesh	934	1.3	0.7–2.2	[32, 33]	4.8	4.0–5.6	[20]	Lower
China^b^	1319	9.4	7.9–11.1	[33, 34]	10.2 ^c^	9.4–11.2	[20]	Comparable
Dutch Antilles	38	2.6	0.1–13.8	[35]	4.5^d^	2.5–6.6	[20]	Comparable
Egypt	465	1.1	0.4–2.5	[36]	4.2 ^c^	1.9–6.5	[20]	Lower
Former USSR	675	4.7	3.3–6.6	[30, 37]	3.8	2.7–4.9	[20]	Comparable
India	1334	0.1	0–0.4	[32, 38]	3.2	2.9–3.6	[20]	Lower
Iran	153	0.7	0.1–2.5	[30]	3.1	2.7–3.5	[20]	Lower
Iraq	290	0.7	0–3.6	[30]	1.3	0–2.9	[20]	Comparable
Morocco	305	0.3	0–1.8	[35, 39]	1.8	1.5–5.9	[20, 22]	Lower
Pakistan	3786	1.6	1.2–2.1	[32, 33, 38, 40]	4.2	3.6–4.8	[20]	Lower
Somalia	317	7.3	4.6–10.7	[41]	12.4	8.9–15.9	[20]	Lower
Suriname	56	0	0–6.4	[35]	4.5^d^	2.5–6.6	[20]	Lower
Turkey	902	3.7	2.5–5.1	[30, 35, 39]	4.3^c^	3.7–4.9	[20]	Comparable
Vietnam	149	10.7	6.3–16.9	[30, 33]	12.5	11.5–13.5	[20]	Lower

Notes:

^a^age range of participants 10–23 years

^b^including Hong Kong

^c^statistically significant decline over time reported therefore the latest estimate (from year 2000 onwards) selected

^d^regional estimate for Caribbean only

In nine of the remaining 14 studies in migrants, HBsAg prevalence figures were lower than the derived in-country estimate. Prevalence among migrants was comparable with the in-country or region estimate for four migrant populations. HBsAg prevalence among migrants from Albania was higher than the in-country estimate.

## Discussion

The number of CHB cases in the general population of the 31 EU/EEA countries is estimated at between 4 million and 7.5 million cases, with a disproportionately high number of these cases found among migrants. Although migrants from endemic countries make up only one in 20 EU/EEA citizens, they account for one in four of all CHB infections. Migrants from ten countries account for 48% of all CHB cases among migrants in the EU/EEA.

The data suggest that the relative contribution of migrants to the overall CHB burden is higher in Western and Northern European countries than in Southern and Eastern European countries. The relative contribution of migrants to the overall burden of CHB is lowest (<4%) in EU/EEA countries with a higher HBsAg prevalence and a lower proportion of migrants such as Romania, Bulgaria, Slovakia and Poland. Conversely, the relative contribution is very high (>100%) in EU/EEA countries with a very low HBsAg prevalence such as Ireland, the Netherlands and Sweden. The estimate of over 100% is a result of the prevalence in the general population of the host country likely to be underestimated (because migrants and other higher risk, harder to reach populations are underrepresented in the samples used to determine this prevalence) or because the prevalence in countries of birth of migrants is an over-estimation of the actual prevalence among migrants.

To assess whether the country of birth prevalence estimates used are over-estimates, we compared these estimates to the prevalence reported in migrant studies in Europe and found evidence indicating an over-estimation. Based on the epidemiologic features of HBV, the lower than in-country prevalence among migrants from 9 of 14 countries was surprising, since the infection is often acquired at birth or in early childhood in countries where HBV is endemic. One explanation could be an age or cohort effect, since the estimates of in-country prevalence include older data from the 1980s and 1990s, while most of the migrant studies in Europe used for comparison were conducted after 2000. A recent study estimating hepatitis B prevalence by region showed a decrease in most regions between 1990 and 2005 [6]. This decrease is largely explained by the widespread introduction of antenatal HBV screening together with risk group and infant HBV vaccination programmes [23] and the resulting decline in incidence. The healthy migrant effect, the hypothesis that migrants are often younger and healthier than the general population in their countries of birth, may also be a factor [24]. Study design should also be considered. The figures for prevalence among migrants living in EU/EEA countries tend to be based on data from small-scale, local studies that mostly use convenience sampling, such as screening studies (Table 5). These may under-estimate the true prevalence because people who have already been diagnosed may not participate. Nevertheless, although prevalence among migrants from intermediate and high endemicity countries may not be as high as in the country of birth, the evidence strongly suggests that it is still considerably higher than among the host population in EU/EEA countries and high enough for screening of affected migrant groups to be cost-effective [11, 25].

This study seeks to inform national screening efforts. The results suggests that an approach focused solely on migrants would have limited impact in most Eastern European countries, specifically Bulgaria, Lithuania, Poland, Romania, Slovakia and Slovenia, where the relative contribution of migrants to the overall national burden of CHB is low (between 1% and 8%) and the proportion of migrants from endemic countries is also low (<5%). In addition, Bulgaria, Lithuania, Romania and Slovenia are HBV-endemic countries with an HBsAg prevalence of >2.0% to 5.5%. A more effective approach would be to screen sub-groups of the general population, i.e. birth cohorts born before antenatal screening, childhood HBV vaccination and the regular screening of blood/blood products were introduced. Screening individuals potentially exposed to HBV through transfusions, transplants and dental or surgical procedures may also help to identify cases.

In contrast, targeted migrant screening approaches would be of value in countries such as Austria, Cyprus, Estonia, Finland, Ireland, the Netherlands, Sweden and UK, where more than 70% of CHB cases are estimated to be among migrants from endemic countries. To optimise cost-effectiveness, screening should target those migrant populations that are most at risk of chronic viral hepatitis infection where the likelihood of detecting cases is higher.

Screening and subsequent contact tracing increases the diagnosis rate and, together with effective linkage to and retention in care and antiviral treatment, are the key to effective secondary prevention. The asymptomatic nature of the disease until its late stages and the lack of awareness of risk among migrants and often also among health care providers limit early detection. Other barriers to screening include language, health care access and entitlement issues, high work-load among health care staff, and the potential stigma or fear associated with being diagnosed positive [26, 27]. The EU-funded HEPscreen project describes four different screening approaches: (i) outreach screening (e.g. through awareness raising and screening sessions in communities or workplaces); (ii) extension of existing screening programmes (e.g. expanding tuberculosis screening to include other diseases); (iii) opportunistic screening (e.g. offering viral hepatitis screening when patients attend for other health care services); and (iv) invitation-based screening (e.g. using municipal or general practice registers). Summaries of screening studies conducted using these different methods, practical guides on how to implement different screening approaches among migrants and resource and logistical considerations are available on the website http://www.hepscreen.eu/ and present a useful resource for public health practitioners [28].

Undocumented migrants are also an important and vulnerable group. Lack of robust demographic data and the diversity in size and country of birth of undocumented migrants [29] hinder effective planning, resourcing and evaluation of screening interventions for this population. In addition, undocumented migrants face specific access and entitlement challenges when accessing public health services. Promoting voluntary screening for this vulnerable group would have public health benefits, but would require national policies that allow undocumented migrants to receive treatment without adverse consequences.

The systematic approach to estimation of the burden of CHB among migrant populations in the EU/EEA overall and in each of the 31 EU/EEA countries is a strength of this study. The data that underpin these estimates are derived from a common demographic data source (Eurostat 2013) for most countries and from published meta-analytic studies. A potential limitation is that, for some countries, demographic data from earlier years and other sources had to be used. In addition, there are differences in population registration and reporting systems between EU/EEA countries. However, using data from 2013 or earlier has the advantage of limiting the effect of reporting delays with respect to accurate numbers of migrants and their countries of birth.

Using country of birth prevalence data to estimate the CHB burden among migrants may have resulted in a degree of over-estimation for some countries, because of differences in the age structure, risk profile and socio-economic status between different migrant groups. Migrants sharing the same country of birth but residing in different EU/EEA countries may also differ in terms of risk profile as a result of differences in host country pull factors and reasons for migration. Data allowing a comparison of prevalence figures between the general population in the migrants’ countries of birth and from studies among migrants in Europe were however only available for 14 countries. An additional strength of this study is that, even if the absolute number of estimated CHB cases lies closer to the lower estimate, it identifies which migrant populations would benefit most from targeted screening programs and linkage to care.

## Conclusions

Today, anti-viral treatments for CHB can benefit most patients, offering the prospect of significant public health gains through secondary prevention. Expanded access to screening, linkage to care and treatment, together with the continued implementation of existing primary prevention measures such as vaccinaiton and antenatal screening, are the cornerstones of eliminating viral hepatitis as a global public health threat in the next few decades.

This study confirms that migrant populations are a key risk group for CHB in specific EU/EEA countries. It details the number of CHB cases among migrants by country of birth in each EU/EEA country, identifies the migrant populations that would benefit most from screening and treatment, and highlights which EU/EEA countries would benefit from a migrant-targeted screening approach. The findings in this study about which migrant groups are at highest risk is also useful for developing linguistically-specific and culturally-sensitive screening programmes and raising awareness among physicians so that they offer screening. Efficient and innovative public health approaches to increase access to screening and to screen high-risk populations are needed. Experience from a migrant-specific screening project conducted at the EU/EEA level [28] can help to inform the design of screening programmes that can successfully reach migrant communities. Planners and practitioners can also use the results presented here and those from the HEPscreen project to develop evidence-based screening interventions that target the most affected migrant populations.

Further research is required to inform the development and assessment of effective and cost-effective screening interventions and long-term patient follow up, as well as to improve linkage to and retention in treatment and care among hard to reach risk populations, if we are to realise the vision of a world free of viral hepatitis.

## Additional file

Additional file 1: Estimating the scale of chronic hepatitis B virus infection among migrants in EU/EEA countries. The ‘Chronic Hepatitis B (CHB) Additional file 1’ includes 9 annexes. Annexs 1, 2, 5 and 7 present data on the search strategy, the PRISMA flow chart, the inclusion and exclusion criteria of global/worldwide systematic reviews and meta-analyses and the rationale for the selected country specific hepatitis B and C prevalence figures. (ii) Annexs 3, 4 and 6 present data on the search strategy, the PRISMA flow chart and the inclusion and exclusion criteria for chronic hepatitis B/C prevalence studies among migrants in the EU/EEA. (iii) Annex 8 presents a list of the country specific HBsAg prevalence estimates selected along with the corresponding source. (iv) Annex 9 presents country tables which list the estimated number of Chronic Hepatitis B (CHB) cases among the 50 largest migrant populations residing in the individual EU/EEA host countries. (DOCX 487 kb)

## Abbreviations

CHB: Chronic hepatitis B
CI: Confidence intervals
EU/EEA: European Union and European Economic Area
HBsAg: Hepatitis B surface antigen
HBV: Hepatitis B virus
HCC: Hepatocellular carcinoma
Migrants: Foreign-born population
OECD: Organisation for Economic Co-operation and Development
PRISMA: Preferred Reporting Items for Systematic Reviews and Meta Analyses
UK: United Kingdom
WHO: World Health Organisation
