# Supplementary material for: Estimating the scale of chronic hepatitis B virus infection among migrants in EU/EEA countries
Source: BMC Infect Dis. 2018 Jan 11;18:34. doi: 10.1186/s12879-017-2921-8 (PMC5765695; doi:10.1186/s12879-017-2921-8)
Supplement: Additional file 1: — Estimating the scale of chronic hepatitis B virus infection among migrants in EU/EEA countries. The ‘Chronic Hepatitis B (CHB) Additional file 1’ includes 9 annexes. Annexs 1, 2, 5 and 7 present data on the search strategy, the PRISMA flow chart, the inclusion and exclusion criteria of global/worldwide systematic reviews and meta-analyses and the rationale for the selected country specific hepatitis B and C prevalence figures. (ii) Annexs 3, 4 and 6 present data on the search strategy, the PRISMA flow chart and the inclusion and exclusion criteria for chronic hepatitis B/C prevalence studies among migrants in the EU/EEA. (iii) Annex 8 presents a list of the country specific HBsAg prevalence estimates selected along with the corresponding source. (iv) Annex 9 presents country tables which list the estimated number of Chronic Hepatitis B (CHB) cases among the 50 largest migrant populations residing in the individual EU/EEA host countries. (DOCX 487 kb) [file 12879_2017_2921_MOESM1_ESM.docx]

**Estimating the burden of chronic hepatitis B virus infection among migrants in EU/EEA countries**

Amena A. AHMAD (corresponding author)^1,2*^, Abby M. FALLA^3,4*^, Erika DUFFELL^5^, Teymur NOORI^5^, Angela BECHINI^6^, Ralf REINTJES^1^, Irene K. VELDHUIJZEN^4,7^

* Both authors contributed equally to the manuscript

^1^ Faculty Life Sciences, Department of Health Sciences, Hamburg University of Applied Sciences, Hamburg, Germany

^2^ Department of Internal Medicine, University Medical Center Hamburg-Eppendorf, Germany

^3^ Department of Public Health, Erasmus MC, University Medical Center Rotterdam, the Netherlands

^4^ Division of Infectious Disease Control, Municipal Public Health Service Rotterdam-Rijnmond, the Netherlands

^5^ European Centre for Disease Prevention and Control, Stockholm, Sweden

^6^ Department of Health Sciences, University of Florence, Florence, Italy

^7^ Center for Infectious Disease Control, National Institute for Public Health and the Environment, Bilthoven, the Netherlands

Corresponding author:

Amena Almes Ahmad; Email: Amenaalmes.Ahmad@haw-hamburg.de; Tel: +49(0)40-42875-6106, Address: Hochschule fuer Angewandte Wissenschaften, Dept. Gesundheitswissenschaften, Ulmenliet 20, 21033 Hamburg, Germany

**Supplementary Chronic Hepatitis B (CHB) Data File**

Table of Contents

[Annex 1: Search strategy for systematic reviews and meta-analyses of global/worldwide prevalence studies 4](#_Toc458018672)

[Annex 2: PRISMA diagram of systematic search for global HBsAg/ANTI-HCV sero-prevalence 7](#_Toc458018673)

[Annex 3: Description of the search strategy and retrievals for chronic hepatitis B/C prevalence studies among migrants 8](#_Toc458018674)

[Annex 4: PRISMA flow diagram of systematic search for HBsAg/ANTI-HCV sero-prevalence in migrants in the EU/EEA 12](#_Toc458018675)

[Annex 5: Inclusion/exclusion criteria for systematic reviews/meta analyses reporting the prevalence worldwide/in various continents/regions 13](#_Toc458018676)

[Annex 6: Inclusion/exclusion criteria for studies retrieved by the search for articles reporting the prevalence among migrants 14](#_Toc458018677)

[Annex 7: Comparison of HBsAg estimates from systematic reviews and meta-analyses and rationale for estimate selected for analysis 15](#_Toc458018678)

[Annex 8: Country-level HBsAg prevalence estimates selected from systematic reviews 18](#_Toc458018679)

[Annex 9 Chronic Hepatitis B burden - country tables: 50 largest migrant populations per EU/EEA country 25](#_Toc458018680)

[9.1 Austria 26](#_Toc458018681)

[9.2 Belguim 27](#_Toc458018682)

[9.3 Bulgaria 28](#_Toc458018683)

[9.4 Croatia 29](#_Toc458018684)

[9.5 Cyprus 30](#_Toc458018685)

[9.6 Czech Republic 31](#_Toc458018686)

[9.7 Denmark 32](#_Toc458018687)

[9.8 Germany 33](#_Toc458018688)

[9.9 Finland 34](#_Toc458018689)

[9.10 Estonia 36](#_Toc458018690)

[9.11 France 37](#_Toc458018691)

[9.12 Greece 38](#_Toc458018692)

[9.13 Hungary 39](#_Toc458018693)

[9.14 Republic of Ireland 40](#_Toc458018694)

[9.15 Iceland 41](#_Toc458018695)

[9.16 Italy 42](#_Toc458018696)

[9.17 Lichtenstein 43](#_Toc458018697)

[9.18 Lithuania 44](#_Toc458018698)

[9.19 Luxembourg 45](#_Toc458018699)

[9.20 Latvia 46](#_Toc458018700)

[9.21 Malta 47](#_Toc458018701)

[9.22 The Netherlands 48](#_Toc458018702)

[9.23 Norway 50](#_Toc458018703)

[9.24 Poland 51](#_Toc458018704)

[9.25 Portugal 52](#_Toc458018705)

[9.26 Romania 53](#_Toc458018706)

[9.27 Slovakia 54](#_Toc458018707)

[9.28 Slovenia 55](#_Toc458018708)

[9.29 Spain 56](#_Toc458018709)

[9.30 Sweden 57](#_Toc458018710)

[9.31 United Kingdom 58](#_Toc458018711)

# Annex 1: Search strategy for systematic reviews and meta-analyses of global/worldwide prevalence studies

The search was structured to include terms from four topic areas, i.e. the infection, the outcome, the population and the study design. Separate search terms are included, as well as controlled vocabulary search terms (EMTree, comparable to MeSH terms in Medline). The search terms included per topic area in the EMBASE search are described below. The full search strategy for systematic reviews in each database is also presented below.

The infection: hepatitis B or C
in abstract or title:
hepatitis B, hepatitis B virus, hepatitis C, hepatitis C virus [EMtree]
hbv, hcv

The outcome: prevalence
in abstract or title:
prevalence, seroprevalence;
serology [EMTree]; prevalen*, seroprevalen*, serolog*, marker*, seroepidemiol*
hepatitis rapid test, hepatitis B rapid test, hepatitis C rapid test [EMTree]
hepatitis B antibody [EMTree]
hepatitis B antigen, hepatitis B surface antigen, HBsAg, HBs-Ag, HB-s-Ag
hepatitis C antigen [EMTree]
hepatitis C antibody [EMTree]

The population: general population worldwide/global:
in abstract or title:
Health survey [EMTree]; 'residual sera', survey*, surveillan*,
Population, Population group, Population research [EMTree]; population*, communit*,
Geographic names, Geographic distribution [EMTree]; geograph*, worldwide, world-wide, global, europe*, asia*, america*, africa*, australia*, countr*
Population and population related phenomena [EMTree];

Study design
in abstract or title:
Systematic review [EMTree]; ‘systematic* AND review*’,
comprehensiv*, exhaustiv* NEAR/3 literature*
Meta analysis [EMTree]; meta NEXT/1 analy*

**Full search strategy for reviews, systematic reviews and meta-analyses on hepatitis B and hepatitis C prevalence worldwide at country level (run by Erasmus MC Medical Library on 21 January 2015)**

**Embase.com 87**((('hepatitis B'/exp OR 'Hepatitis B virus'/exp OR 'hepatitis C'/exp OR 'Hepatitis C virus'/exp OR ((hepatitis NEXT/3 (B OR C)) OR hbv OR hcv):ab,ti) AND (prevalence/de OR seroprevalence/de OR serology/exp OR epidemiology/exp OR (seroprevalen* OR prevalen* OR serolog* OR seroepidemiol* OR epidemiolog*):ab,ti)) OR ('hepatitis rapid test'/exp OR 'hepatitis B rapid test'/exp OR 'hepatitis C rapid test'/exp OR 'hepatitis B antigen'/de OR 'hepatitis C antigen'/exp OR 'hepatitis C antibody'/exp OR 'hepatitis B antibody'/exp OR 'hepatitis B surface antigen'/de OR (HBsAg OR HBs-Ag OR HB-s-Ag OR (('Hepatitis b' OR 'Hepatitis c' OR hbv OR hcv) NEAR/3 ( 'surface antigen' OR 'surface antigens' OR antibod*))):ab,ti)) AND ('health survey'/de OR population/de OR 'population group'/exp OR 'geographic names'/exp OR 'population research'/exp OR 'geographic distribution'/exp OR 'population and population related phenomena'/exp OR (population* OR communit* OR 'residual sera' OR survey* OR surveillan* OR geograph* OR worldwide OR world-wide OR global OR europe* OR asia* OR america* OR africa* OR australia* OR countr*):ab,ti) AND ('systematic review'/exp OR 'meta analysis'/exp OR ((systematic* AND review*) OR ((comprehensiv* OR exhaustiv*) NEAR/3 literature*) OR (meta NEXT/1 analy*)):ab,ti)

**Medline (OvidSP) 326**(((exp hepatitis B/ OR Hepatitis B virus/ OR hepatitis C/ OR Hepacivirus/ OR ((hepatitis ADJ3 (B OR C)) OR hbv OR hcv).ab,ti.) AND (prevalence/ OR Seroepidemiologic Studies/ OR serology/ OR Serologic Tests/ OR (seroprevalen* OR prevalen* OR serolog* OR seroepidemiol* OR epidemiolog*).ab,ti.)) OR (exp hepatitis B antigen/ OR hepatitis C antigen/ OR hepatitis C antibodies/ OR hepatitis B antibodies/ OR (HBsAg OR HBs-Ag OR HB-s-Ag OR ((Hepatitis b OR Hepatitis c OR hbv OR hcv) ADJ3 ( surface antigen OR surface antigens OR antibod*))).ab,ti.)) AND (exp Health Surveys/ OR exp Population Groups/ OR exp Population/ OR exp Geographic Locations/ OR (population* OR communit* OR residual sera OR survey* OR surveillan* OR geograph* OR worldwide OR world-wide OR global OR europe* OR asia* OR america* OR africa* OR australia* OR countr*).ab,ti.) AND (Meta-Analysis.pt. OR ((systematic* AND review*) OR ((comprehensiv* OR exhaustiv*) ADJ3 literature*) OR (meta ADJ analy*)).ab,ti.)

**Cochrane DARE 0**(((((hepatitis NEXT/3 (B OR C)) OR hbv OR hcv):ab,ti) AND ((seroprevalen* OR prevalen* OR serolog* OR seroepidemiol* OR epidemiolog*):ab,ti)) OR ((HBsAg OR HBs-Ag OR HB-s-Ag OR (('Hepatitis b' OR 'Hepatitis c' OR hbv OR hcv) NEAR/3 ( 'surface antigen' OR 'surface antigens' OR antibod*))):ab,ti)) AND ((population* OR communit* OR 'residual sera' OR survey* OR surveillan* OR geograph* OR worldwide OR world-wide OR global OR europe* OR asia* OR america* OR africa* OR australia* OR countr*):ab,ti)

**Web-of-science 366**TS=((((((hepatitis NEAR/3 (B OR C)) OR hbv OR hcv)) AND ((seroprevalen* OR prevalen* OR serolog* OR seroepidemiol* OR epidemiolog*))) OR ((HBsAg OR HBs-Ag OR HB-s-Ag OR (("Hepatitis b" OR "Hepatitis c" OR hbv OR hcv) NEAR/3 ( "surface antigen" OR "surface antigens" OR antibod*))))) AND ((population* OR communit* OR "residual sera" OR survey* OR surveillan* OR geograph* OR worldwide OR world-wide OR global OR europe* OR asia* OR america* OR africa* OR australia* OR countr*)) AND (((systematic* AND review*) OR ((comprehensiv* OR exhaustiv*) NEAR/3 literature*) OR (meta NEAR/1 analy*))))

**PubMed publisher 8**(((hepatitis B[mh] OR Hepatitis B virus[mh] OR hepatitis C[mh] OR Hepacivirus[mh] OR ((hepatitis AND (B OR C)) OR hbv OR hcv)) AND (prevalence[mh] OR Seroepidemiologic Studies[mh] OR serology[mh] OR Serologic Tests[mh] OR (seroprevalen*[tiab] OR prevalen*[tiab] OR serolog*[tiab] OR seroepidemiol*[tiab] OR epidemiolog*[tiab]))) OR (hepatitis B antigen[mh] OR hepatitis C antigen[mh] OR hepatitis C antibodies[mh] OR hepatitis B antibodies[mh] OR (HBsAg OR HBs-Ag OR HB-s-Ag OR ((Hepatitis b OR Hepatitis c OR hbv OR hcv) AND ( surface antigen OR surface antigens OR antibod*[tiab]))))) AND (Health Surveys[mh] OR Population Groups[mh] OR Population[mh] OR Geographic Locations[mh] OR (population*[tiab] OR communit*[tiab] OR residual sera OR survey*[tiab] OR surveillan*[tiab] OR geograph*[tiab] OR worldwide OR world-wide OR global OR europe*[tiab] OR asia*[tiab] OR america*[tiab] OR africa*[tiab] OR australia*[tiab] OR countr*[tiab])) AND (Meta-Analysis[pt] OR ((systematic*[tiab] AND review*[tiab]) OR ((comprehensiv*[tiab] OR exhaustiv*[tiab]) AND literature*[tiab]) OR (meta analy*[tiab]))) AND publisher

**Google Scholar**"hepatitis B|C"|hbv|hcv seroprevalence|prevalence|serology|seroepidemioly|epidemiology population|community|geography|worldwide|"world wide"|global|europe|asia|america|africa|australia|country|countries "systematic review|"meta analysis"

# Annex 2: PRISMA diagram of systematic search for global HBsAg/ANTI-HCV sero-prevalence

Records identified through database searching n = 1772

Records after duplicates removed
n = 1182

Records screened
n = 802

Records excluded based on title/abstract n=757

Full-text articles assessed for eligibility
n = 45

Full-text articles excluded, with reasons n = 27

Studies included in qualitative synthesis

N = 18

Records after exclusion of articles published before 2009 n = 802

| **Full Text Exclusion: Reasons** | **N** |
| --- | --- |
| No full text available | 4 |
| No prevalence estimate | 5 |
| Conference abstract (full paper available or insufficient detail to assess the methodological quality | 6 |
| Unrepresentative for or no data on the general population | 5 |
| Not a systematic review/meta analysis | 3 |
| Poor/limited search | 2 |
| Methodologically vague | 1 |
| Protocol only | 1 |
| Total |  |

# Annex 3: Description of the search strategy and retrievals for chronic hepatitis B/C prevalence studies among migrants

The search was structured to include terms from four topic areas, i.e. the infection, the outcome, the population and geographical area. Separate search terms are included, as well as controlled vocabulary search terms (EMTree, comparable to MeSH terms in Medline). The search terms included per topic area in the EMBASE search are described below. The full search strategy for migrant studies in each database is also presented below.

The infection: hepatitis B or C
in abstract or title:
hepatitis B, Hepatitis B virus, hepatitis C, hepatitis C virus [EMtree]
hbv, hcv

The outcome: prevalence
in abstract or title:
prevalence, seroprevalence;
serology [EMTree]; prevalen*, seroprevalen*, serolog*, marker*, seroepidemiol*
hepatitis rapid test, hepatitis B rapid test, hepatitis C rapid test [EMTree]
hepatitis B antibody [EMTree]
hepatitis B antigen, hepatitis B surface antigen, HBsAg, HBs-Ag, HB-s-Ag
hepatitis C antigen [EMTree]
hepatitis C antibody [EMTree]

The population: migrants:
in abstract or title:
migrant, migration, minority group, ethnic group, ethnicity [EMTree]
migrant*, emigrant*, immigrant*, migrat*, emigrat*, immigrat*, asylum seeker*, refugee*, minorit*, ethnic*
(countr*, africa*, asia*, racial*, eastern europe, endemic areas, endemic area, foreign*) NEAR (origin*, born*, nationalit*)

Geographical area: EU/EEA countries
in abstract or title or journal title or country of journal or country of author or author address:
Europe, European [EMTree]
European Union, europ*, eu
Austria*, Belgium, belgian, Bulgaria*, Croatia*, Cyprus, cypriot*, Czech*, Denmark, danish, Estonia*, Finland, finnish, finn, France, french*, German*, Greece, greek, Hungar*, Ireland, irish, Italy, italian*, Latvia*, Lithuania*, Luxemburg*, Malta, maltese, Netherlands, dutch, Poland, polish*, Portug*, Romania*, Slovak*, Slovenia*, Spain, spanish, spaniard*, Sweden, swedish, "united kingdom", "great britain", uk, british*, England, english, Scotland, scottish, Wales, welsh, Norway, norweg*, Iceland*

**Full search strategy: Hepatitis B and C among migrants in the EU (run by Erasmus MC Medical Library on 17 November 2014)**

**Embase.com 1210 (1204 unique records)**((('hepatitis B'/exp OR 'Hepatitis B virus'/exp OR 'hepatitis C'/exp OR 'Hepatitis C virus'/exp OR ((hepatitis NEAR/3 (B OR C)) OR hbv OR hcv):ab,ti) AND (prevalence/de OR seroprevalence/de OR serology/exp OR (seroprevalen* OR prevalen* OR marker* OR serolog* OR seroepidemiol*):ab,ti)) OR ('hepatitis rapid test'/exp OR 'hepatitis B rapid test'/exp OR 'hepatitis C rapid test'/exp OR 'hepatitis B antigen'/de OR 'hepatitis C antigen'/exp OR 'hepatitis C antibody'/exp OR 'hepatitis B antibody'/exp OR 'hepatitis B surface antigen'/de OR (HBsAg OR HBs-Ag OR HB-s-Ag OR (('Hepatitis b' OR 'Hepatitis c' OR hbv OR hcv) NEAR/3 ( 'surface antigen' OR 'surface antigens' OR antibod*))):ab,ti)) AND (migrant/exp OR migration/exp OR 'minority group'/exp OR 'ethnic group'/exp OR ethnicity/exp OR (migrant* OR emigrant* OR immigrant* OR migrat* OR emigrat* OR immigrat* OR (asylum NEXT/1 seeker*) OR refugee* OR minorit* OR ethnic* OR ((countr* OR africa* OR asia* OR racial* OR 'eastern europe' OR 'endemic areas' OR 'endemic area' OR foreign* ) NEAR/3 (origin* OR born* OR nationalit*))):ab,ti) AND (europe/exp OR European/exp OR 'European Union'/de OR (europ* OR eu OR iceland* OR norway OR norweg* OR sweden OR swedish OR finland OR finnish OR finn OR denmark OR danish OR "great britain" OR "united kingdom" OR uk OR british* OR England OR english OR Scotland OR scottish OR Wales OR welsh OR Ireland OR irish OR Netherlands OR dutch OR Belgium OR belgian OR France OR french* OR Luxemburg* OR Spain OR spanish OR spaniard* OR Portug* OR Italy OR italian* OR Switzerland OR swiss* OR Austria* OR German* OR Poland OR polish* OR Hungar* OR Czech* OR Croatia* OR Slovak* OR Slovenia* OR Romania* OR Bulgaria* OR Lithuania* OR Latvia* OR Estonia* OR Greece OR greek OR Turkey OR turkish OR Macedonia* OR Cyprus OR cypriot* OR Malta OR maltese):ab,ti,ca,ta,cy,ad) AND [english]/lim NOT ([Conference Abstract]/lim OR [Letter]/lim OR [Note]/lim OR [Conference Paper]/lim OR [Editorial]/lim)

**Medline (OvidSP) 989 (191 unique records)**(((exp hepatitis B/ OR Hepatitis B virus/ OR hepatitis C/ OR Hepacivirus/ OR ((hepatitis ADJ3 (B OR C)) OR hbv OR hcv).ab,ti.) AND (prevalence/ OR Seroepidemiologic Studies/ OR serology/ OR Serologic Tests/ OR (seroprevalen* OR prevalen* OR marker* OR serolog* OR seroepidemiol*).ab,ti.)) OR (exp hepatitis B antigen/ OR hepatitis C antigen/ OR hepatitis C antibodies/ OR hepatitis B antibodies/ OR (HBsAg OR HBs-Ag OR HB-s-Ag OR ((Hepatitis b OR Hepatitis c OR hbv OR hcv) ADJ3 (surface antigen* OR antibod*))).ab,ti.)) AND (Transients and Migrants/ OR Human Migration/ OR minority group/ OR ethnic group/ OR Emigrants and Immigrants/ OR Emigration and Immigration/ OR (migrant* OR emigrant* OR immigrant* OR migrat* OR emigrat* OR immigrat* OR asylum seeker* OR refugee* OR minorit* OR ethnic* OR ((countr* OR africa* OR asia* OR racial* OR eastern europe OR endemic areas OR endemic area OR foreign* ) ADJ3 (origin* OR born* OR nationalit*))).ab,ti.) AND (exp europe/ OR (europ* OR eu OR iceland* OR norway OR norweg* OR sweden OR swedish OR finland OR finnish OR finn OR denmark OR danish OR "great britain" OR "united kingdom" OR uk OR british* OR England OR english OR Scotland OR scottish OR Wales OR welsh OR Ireland OR irish OR Netherlands OR dutch OR Belgium OR belgian OR France OR french* OR Luxemburg* OR Spain OR spanish OR spaniard* OR Portug* OR Italy OR italian* OR Switzerland OR swiss* OR Austria* OR German* OR Poland OR polish* OR Hungar* OR Czech* OR Croatia* OR Slovak* OR Slovenia* OR Romania* OR Bulgaria* OR Lithuania* OR Latvia* OR Estonia* OR Greece OR greek OR Turkey OR turkish OR Macedonia* OR Cyprus OR cypriot* OR Malta OR maltese).ab,ti,jn,cp,in.) AND english.la. NOT (letter OR news OR comment OR editorial OR congresses OR abstracts).pt.

**Cochrane 8 (3 unique records)**(((((hepatitis NEAR/3 (B OR C)) OR hbv OR hcv):ab,ti) AND ((seroprevalen* OR prevalen* OR marker* OR serolog* OR seroepidemiol*):ab,ti)) OR ((HBsAg OR HBs-Ag OR HB-s-Ag OR (('Hepatitis b' OR 'Hepatitis c' OR hbv OR hcv) NEAR/3 ( 'surface antigen' OR 'surface antigens' OR antibod*))):ab,ti)) AND ((migrant* OR emigrant* OR immigrant* OR migrat* OR emigrat* OR immigrat* OR (asylum NEXT/1 seeker*) OR refugee* OR minorit* OR ethnic* OR ((countr* OR africa* OR asia* OR racial* OR 'eastern europe' OR 'endemic areas' OR 'endemic area' OR foreign* ) NEAR/3 (origin* OR born* OR nationalit*))):ab,ti) AND ((europ* OR eu OR iceland* OR norway OR norweg* OR sweden OR swedish OR finland OR finnish OR finn OR denmark OR danish OR "great britain" OR "united kingdom" OR uk OR british* OR England OR english OR Scotland OR scottish OR Wales OR welsh OR Ireland OR irish OR Netherlands OR dutch OR Belgium OR belgian OR France OR french* OR Luxemburg* OR Spain OR spanish OR spaniard* OR Portug* OR Italy OR italian* OR Switzerland OR swiss* OR Austria* OR German* OR Poland OR polish* OR Hungar* OR Czech* OR Croatia* OR Slovak* OR Slovenia* OR Romania* OR Bulgaria* OR Lithuania* OR Latvia* OR Estonia* OR Greece OR greek OR Turkey OR turkish OR Macedonia* OR Cyprus OR cypriot* OR Malta OR maltese):ab,ti)

**Web-of-science 547 (170 unique records)**TS=((((((hepatitis NEAR/3 (B OR C)) OR hbv OR hcv)) AND ((seroprevalen* OR prevalen* OR marker* OR serolog* OR seroepidemiol*))) OR ((HBsAg OR HBs-Ag OR HB-s-Ag OR (("Hepatitis b" OR "Hepatitis c" OR hbv OR hcv) NEAR/3 ( "surface antigen" OR "surface antigens" OR antibod*))))) AND ((migrant* OR emigrant* OR immigrant* OR migrat* OR emigrat* OR immigrat* OR (asylum NEAR/1 seeker*) OR refugee* OR minorit* OR ethnic* OR ((countr* OR africa* OR asia* OR racial* OR "eastern europe" OR "endemic areas" OR "endemic area" OR foreign* ) NEAR/3 (origin* OR born* OR nationalit*)))) AND ((europ* OR eu OR iceland* OR norway OR norweg* OR sweden OR swedish OR finland OR finnish OR finn OR denmark OR danish OR "great britain" OR "united kingdom" OR uk OR british* OR England OR english OR Scotland OR scottish OR Wales OR welsh OR Ireland OR irish OR Netherlands OR dutch OR Belgium OR belgian OR France OR french* OR Luxemburg* OR Spain OR spanish OR spaniard* OR Portug* OR Italy OR italian* OR Switzerland OR swiss* OR Austria* OR German* OR Poland OR polish* OR Hungar* OR Czech* OR Croatia* OR Slovak* OR Slovenia* OR Romania* OR Bulgaria* OR Lithuania* OR Latvia* OR Estonia* OR Greece OR greek OR Turkey OR turkish OR Macedonia* OR Cyprus OR cypriot* OR Malta OR maltese)) )

**Scopus 1012 (304 unique records)**TITLE-ABS-KEY((((((hepatitis W/3 (B OR C)) OR hbv OR hcv)) AND ((seroprevalen* OR prevalen* OR marker* OR serolog* OR seroepidemiol*))) OR ((HBsAg OR HBs-Ag OR HB-s-Ag OR (("Hepatitis b" OR "Hepatitis c" OR hbv OR hcv) W/3 ( "surface antigen" OR "surface antigens" OR antibod*))))) AND ((migrant* OR emigrant* OR immigrant* OR migrat* OR emigrat* OR immigrat* OR (asylum W/1 seeker*) OR refugee* OR minorit* OR ethnic* OR ((countr* OR africa* OR asia* OR racial* OR "eastern europe" OR "endemic areas" OR "endemic area" OR foreign* ) W/3 (origin* OR born* OR nationalit*)))) AND ((europ* OR eu OR iceland* OR norway OR norweg* OR sweden OR swedish OR finland OR finnish OR finn OR denmark OR danish OR "great britain" OR "united kingdom" OR uk OR british* OR England OR english OR Scotland OR scottish OR Wales OR welsh OR Ireland OR irish OR Netherlands OR dutch OR Belgium OR belgian OR France OR french* OR Luxemburg* OR Spain OR spanish OR spaniard* OR Portug* OR Italy OR italian* OR Switzerland OR swiss* OR Austria* OR German* OR Poland OR polish* OR Hungar* OR Czech* OR Croatia* OR Slovak* OR Slovenia* OR Romania* OR Bulgaria* OR Lithuania* OR Latvia* OR Estonia* OR Greece OR greek OR Turkey OR turkish OR Macedonia* OR Cyprus OR cypriot* OR Malta OR maltese)) )

**PubMed publisher 37 (27 unique records)**(((hepatitis B*[tiab] OR hepatitis C*[tiab] OR hbv[tiab] OR hcv[tiab]) AND ((seroprevalen*[tiab] OR prevalen*[tiab] OR marker*[tiab] OR serolog*[tiab] OR seroepidemiol*[tiab] OR surface antigen*[tiab] OR antibod*[tiab]))) OR ((HBsAg[tiab] OR HBs-Ag[tiab] OR HB-s-Ag[tiab] ))) AND ((migrant*[tiab] OR emigrant*[tiab] OR immigrant*[tiab] OR migrat*[tiab] OR emigrat*[tiab] OR immigrat*[tiab] OR asylum seeker*[tiab] OR refugee*[tiab] OR minorit*[tiab] OR ethnic*[tiab] OR ((countr*[tiab] OR africa*[tiab] OR asia*[tiab] OR racial*[tiab] OR eastern europe*[tiab] OR endemic area*[tiab] OR foreign*[tiab] ) AND (origin*[tiab] OR born*[tiab] OR nationalit*[tiab])))) AND (europ* OR eu OR iceland* OR norway OR norweg* OR sweden OR swedish OR finland OR finnish OR finn OR denmark OR danish OR "great britain" OR "united kingdom" OR uk OR british* OR England OR english OR Scotland OR scottish OR Wales OR welsh OR Ireland OR irish OR Netherlands OR dutch OR Belgium OR belgian OR France OR french* OR Luxemburg* OR Spain OR spanish OR spaniard* OR Portug* OR Italy OR italian* OR Switzerland OR swiss* OR Austria* OR German* OR Poland OR polish* OR Hungar* OR Czech* OR Croatia* OR Slovak* OR Slovenia* OR Romania* OR Bulgaria* OR Lithuania* OR Latvia* OR Estonia* OR Greece OR greek OR Turkey OR turkish OR Macedonia* OR Cyprus OR cypriot* OR Malta OR maltese) AND english[la] AND publisher

**Google Scholar 200 (155 unique records)**"hepatitis B|C" prevalence|seroprevalence|antigen|antibody|antigens|antibodies
migrant|migrants|minority|minorities|ethnic|ethnicity|immigrants|immigrant|foreigners europe|European

# Annex 4: PRISMA flow diagram of systematic search for HBsAg/ANTI-HCV sero-prevalence in migrants in the EU/EEA

Records excluded based on title/abstract

n = 1365

Records retrieved via systematic search
n = 4003

Records after duplicates removed
n = 2054

Records after exclusion of articles published before 2000

n = 1443

Records screened
n = 1443

Full-text articles assessed for eligibility

n = 78

Records excluded based on full-text (reasons documented)

n =22

Studies included in qualitative synthesis

n = 56

| Reason for exclusion | N |
| --- | --- |
| Not in English | 14 |
| Commentary only | 1 |
| Modelled data only | 1 |
| Unspecified virological markers | 1 |
| No data on migrants | 4 |
| Unreliable/unknown denominator | 1 |
| Total | 22 |

# Annex 5: Inclusion/exclusion criteria for systematic reviews/meta analyses reporting the prevalence worldwide/in various continents/regions

Studies retrieved by the search for systematic reviews/meta analyses reporting the prevalence worldwide/in various continents/regions will be assessed by the following criteria:

Inclusion criteria:

- Studies must be of a systematic review and/or meta analytic methodological design
- Studies must include primary research reporting HBsAg/anti-HCV (and/or HBV DNA / HCV RNA) prevalence in humans measured via (laboratory) testing of samples of bodily fluid(s)

Exclude:

- Guidelines, opinion pieces or commentaries that are not systematic reviews/meta analyses of prevalence estimates
- Self-reported HBsAg/anti-HCV status
- Studies with unspecified virological markers of hepatitis B / C infection
- Modelling studies with no original data
- Environmental studies, technology assessments (studies on diagnostic and/or laboratory methods)

# Annex 6: Inclusion/exclusion criteria for studies retrieved by the search for articles reporting the prevalence among migrants

Studies retrieved by the search for articles reporting the prevalence among migrants will be assessed by the following criteria:

Inclusion criteria:

- Studies must suggest/report HBsAg/anti-HCV (and/or HBV DNA/HCV RNA) prevalence in humans measured via (laboratory) testing of samples of bodily fluid(s)
- Studies must be among (first or second generation) migrants of any age to any of the 31 EU/EEA countries
- Studies must report prevalence by country of birth/ethnicity/region
- Studies can be among any migrant sub-population such as refugees, asylum seekers and undocumented migrants
- Studies can be among adults, pregnant women and/or children/adolescents

Exclude:

- Guidelines, opinion pieces or commentaries that are not original research of prevalence estimates
- Studies in Roma populations unless explicitly about Roma groups that are migrants (i.e. with a country of birth outside the ‘host’ country)
- Self-reported HBsAg/anti-HCV status
- Studies with unspecified virological markers of hepatitis B/C infection
- Modelling studies with no original data
- Environmental studies, technology assessments (studies on diagnostic and/or laboratory methods)

# Annex 7: Comparison of HBsAg estimates from SYSTEMATIC REVIEWS AND META-ANALYSES and rationale for estimate selected for analysis

Kowdley included studies published between 1980 and July 2010. Both studies in the general populations of countries of origin of migrants (1,797 surveys in 98 countries, including >17 million persons) as well as 256 surveys in emigrants from 52 countries (including 689,078 persons) were included. Kowdley analysed if a time trend was present over the period before 1990, 1990-2000 and after 2000. If the meta regression analysis did not report a significant time trend we took the overall prevalence estimate. When a significant time trend was observed we took the estimate from the period after 2000. For the following 11 countries a significant decrease in prevalence over time was observed and therefore the estimate from the period after 2000 was taken: China, Egypt, Ethiopia, Greece, Italy, Romania, Saudi Arabia, South Korea, Spain, Thailand and Turkey.

Where Kowdley reports a regional estimate for a country we checked if other reviews providing country estimates are available and whether the reported estimates from other studies are considered more reliable. Studies compared:

Ali, S. A., R. M. J. Donahue, et al. (2009). "Hepatitis B and hepatitis C in Pakistan: prevalence and risk factors." Int J Infect Dis **13**(1): 9-19.

Ali, M., M. Idrees, et al. (2011). "Hepatitis B virus in Pakistan: A systematic review of prevalence, risk factors, awareness status and genotypes." Virol J **8**.

Batham, A., M. A. Gupta, et al. (2009). "Calculating prevalence of hepatitis B in India: Using population weights to look for publication bias in conventional meta-analysis." Indian J Pediatr **76**(12): 1247-1257.

Ezzikouri, S., P. Pineau, et al. (2013). "Hepatitis B virus in the Maghreb Region: From epidemiology to prospective research." Liver Int **33**(6): 811-819.

Hahne, S. J. M., I. K. Veldhuijzen, et al. (2013). "Infection with hepatitis B and C virus in Europe: A systematic review of prevalence and cost-effectiveness of screening." BMC Infect Dis **13**(1).

Hope, V. D., I. Eramova, et al. (2014). "Prevalence and estimation of hepatitis B and C infections in the WHO European Region: A review of data focusing on the countries outside the European Union and the European Free Trade Association." Epidemiol Infect **142**(2): 270-286.

Lehman, E. M. and M. L. Wilson (2009). "Epidemiology of hepatitis viruses among hepatocellular carcinoma cases and healthy people in Egypt: A systematic review and meta-analysis." Int J Cancer **124**(3): 690-697.

Albania
HBsAg prevalence reported in Hope is 9.0% HBsAg (95% CI 8.1-9.8 (back calculated using sample size and prevalence - the Fisher exact CI).and derived from three studies (two national studies and one city-specific study). Kowdley includes eight general population and ten immigrant studies and reports a prevalence of 12.39% (95% CI 9.75 - 5.03). The studies in immigrants are dominated by refugees, therefore we feel that the Hope estimate is a more accurate estimate.

Maghreb region (Tunisia, Algeria, Libya, Morocco, Mauritania)
Estimates from Ezzikouri (2013) are generally lower than Kowdley (where they are available). These estimates include studies in French and unpublished national report data. We therefore think they are a more accurate estimate of HBsAg prevalence. However, a CI is not provided. As there are no estimates in Kowdley for Tunisia and Libya, we would have to use the region estimate from North Africa which includes Sudan and is therefore an overestimate for these countries.

For Algeria and Morocco we take Ezzikouri’s point estimate and keep Kowdleys CI.

For Libya and Tunisia we take Ezzikouri’s point estimate and the CI of Morocco as we feel this CI is more representative of these countries than the regional CI for North Africa which includes Sudan.

The Ezzikouri estimate for Mauritania is higher (18.5%) than the Kowdley regional estimate (13.2%). As the Ezzikouri point estimate falls outside the regional CI we keep regional estimate for West Africa from Kowdley.

Egypt
Lehman et al is based on 12 studies all pre-2007 in populations including voluntary blood donors, antenatal screening, military recruits. This is not considered a better estimate than the Kowdley study estimate from studies after 2000. We prefer the estimated prevalence of 4.2% in Kowdley compared to 8% in Lehman.

Germany
The Hahné et al review reports the same prevalence as Kowdley. No difference so we keep Kowdley.

Greece
The estimate from the Hahné review (2.1%) was based on one survey and is comparable to the recent Kowdley estimate (2.3%). As the recent Kowdley estimate was based on nine sero-surveys we prefer this estimate.

India
One study (Batham) is based on 54 studies all pre-2009 in populations including voluntary and replacement blood donors. We prefer the Kowdley study which included 74 in country estimates. The estimates are not too different (3.2% in Kowdley compared to 3.1% in Batham).

Republic of Ireland
Kowdley reports 0.35%, based on a study in 16,000 pregnant women in Dublin 1998-2000. The Hahné et al review estimate of 0.1% based on one study of 2,500 residual sera collected in 2003. This last study is nationwide so we prefer this estimate over Kowdley.

Italy
The Hahné review does not report an overall country estimate but estimates for Italian regions. We keep the Kowdley national estimate.

Kazakhstan
Kowdley reports 4.95%, Hope 3.8%. Prefer Kowdley as this is based on 4 studies while Hope is based on one study only.

Netherlands
Kowdley reports 0.41%, based on a study from Amsterdam in 2004. Hahné review reports 0.1% based on a nationwide study in 1995-1996. This last study is nationwide so we prefer this estimate over Kowdley.

Pakistan
Two studies (Ali, 2009 and Ali, 2011) estimated prevalence in Pakistan. Ali (2009) included 13 publications estimating the prevalence in the general (non-blood donor) population to arrive at 3.8% (95% CI 1.4 -11). Ali 2011 included 17 studies. Kowdley included 32 in country estimates, most of which (24) were published after 2000. We therefore deem Kowdley to be the most reliable estimate.

Romania
Kowdley reports 5.5%. Hahné et al report 5.6%. Since the estimates are similar and Hahné et al was based on one study only, we prefer the most recent Kowdley recent estimate.

Russia
Kowdley reports 2.89%, Hope reports 1.5%. Hope based on one conference paper from 2008. Kowdley based on 19 estimates. We therefore prefer the Kowdley estimate.

Spain
Recent Kowdley of estimate 0.66% based on 8 surveys. Hahné 1.0% but based on one study from Catalonia only.

Turkey
Hope and Toy estimate the prevalence in Turkey. Hope included nine studies and estimated a prevalence of 3.4%. Toy included 30 studies from 1999-2009 and estimates 4.8%. Kowdley included 69 in country studies with an overall estimate of 5.3%. We prefer the >2000 Kowdley estimate.

Ukraine
Kowdley reports 2.2% whereas Hope reports 1.3%. Both based on 3 surveys but Hope includes 2 surveys in pregnant women so we do not consider this a better estimate than Kowdley.

Uzbekistan
Kowdley reports 6.34%, based on 10 studies. Hope reports 13%, based on one study. We therefore prefer Kowdley estimate.

# Annex 8: Country-level HBsAg prevalence estimates selected from systematic reviews

| **Country** | **HBsAg Prevalence** | **LL 95%CI** | **UL 95%CI** | **Endemicity** | **Level of data** | **GBD Region** | **Data Source** |
| --- | --- | --- | --- | --- | --- | --- | --- |
| Afghanistan | 10.5 | 5.9 | 15.1 | High | Country | South Asia | Kowdley et.al. |
| Albania | 9.0 | 8.1 | 9.8 | High | Country | Central Europe | Hope et. al. |
| Algeria | 2.6 | 0.0 | 12.2 | High-Intermediate | Country | North Africa/Middle East | Ezzikouri et. al. |
| Angola | 11.4 | 8.5 | 14.4 | High | Regional | Middle Africa | Kowdley et.al. |
| Argentina | 1.0 | 0.4 | 1.6 | Low | Country | Southern Latin America | Kowdley et.al. |
| Armenia | 0.6 | 0.2 | 0.9 | Very Low | Country | Central Asia | Kowdley et.al. |
| Australia | 0.9 | 0.4 | 1.4 | Very Low | Country | Australasia | Kowdley et.al. |
| Austria | 0.6 | 0.3 | 0.7 | Very Low | Regional | Western Europe | Kowdley et.al. |
| Azerbaijan | 3.1 | 1.4 | 4.8 | Low-Intermediate | Country | Central Asia | Kowdley et.al. |
| Bangladesh | 4.8 | 4.0 | 5.6 | High-Intermediate | Country | South Asia | Kowdley et.al. |
| Barbados | 0.4 | 0.2 | 0.7 | Very Low | Country | Caribbean | Kowdley et.al. |
| Belarus | 3.2 | 1.7 | 4.7 | Low-Intermediate | Country | Eastern Europe | Kowdley et.al. |
| Belgium | 0.7 | 0.4 | 1.2 | Very Low | Country | Western Europe | Hahné et al. |
| Belize | 1.3 | 0.7 | 1.8 | Low | Country | Caribbean | Kowdley et.al. |
| Benin | 13.2 | 10.2 | 16.2 | High | Regional | West Sub-Saharan Africa | Kowdley et.al. |
| Bolivia | 3.0 | 0.1 | 6.0 | Low-Intermediate | Country | Andean Latin America | Kowdley et.al. |
| Bosnia and Herzegovina | 3.6 | 2.3 | 5.0 | Low-Intermediate | Country | Central Europe | Kowdley et.al. |
| Brazil | 1.8 | 1.5 | 2.0 | Low | Country | Tropical Latin America | Kowdley et.al. |
| Bulgaria | 4.3 | 2.8 | 5.7 | High-Intermediate | Country | Central Europe | Kowdley et.al. |
| Burundi | 9.7 | 7.1 | 12.3 | High | Regional | Eastern Sub-Saharan Africa | Kowdley et.al. |
| Cambodia | 10.3 | 7.0 | 13.5 | High | Country | Southeast Asia | Kowdley et.al. |
| Cameroon | 11.4 | 8.5 | 14.4 | High | Country | West Africa | Kowdley et.al. |
| Canada | 0.7 | 0.6 | 0.9 | Very Low | Country | High Income North America | Kowdley et.al. |
| Cape Verde | 5.7 | 0.2 | 11.1 | High-Intermediate | Country | West Africa | Kowdley et.al. |
| Chad | 11.4 | 8.5 | 14.4 | High | Regional | Middle Africa | Kowdley et.al. |
| Chile | 0.4 | 0.1 | 0.7 | Very Low | Country | Southern Latin America | Kowdley et.al. |
| China | 10.2 | 9.4 | 11.1 | High | Country^β^ | East Asia | Kowdley et.al. |
| Colombia | 1.2 | 0.3 | 2.1 | Low | Country | Central Latin America | Kowdley et.al. |
| Comoros | 9.7 | 7.1 | 12.3 | High | Regional | Eastern Africa | Kowdley et.al. |
| Congo | 11.4 | 8.5 | 14.4 | High | Regional | Middle Africa | Kowdley et.al. |
| Costa Rica | 0.6 | 0.0 | 1.2 | Very Low | Country | Central Latin America | Kowdley et.al. |
| Croatia | 1.5 | 0.8 | 2.1 | Low | Country | Central Europe | Kowdley et.al. |
| Cuba | 0.9 | 0.6 | 1.1 | Very Low | Country | Caribbean | Kowdley et.al. |
| Cyprus | 0.9 | 0.3 | 2.0 | Very Low | Country | Western Europe | Hahné et. al. |
| Czech Republic | 0.7 | 0.4 | 1.0 | Very Low | Country | Central Europe | Kowdley et.al. |
| Czech Republic and Slovakia | 0.7 | 0.4 | 1.0 | Very Low | Country | Central Europe | Kowdley et.al. |
| Democratic Republic of the Congo | 11.4 | 8.5 | 14.4 | High | Regional | Middle Africa | Kowdley et.al. |
| Denmark | 0.6 | 0.3 | 0.7 | Very Low | Regional | Western Europe | Kowdley et.al. |
| Dominican Republic | 10.7 | 5.9 | 15.5 | High | Country | Caribbean | Kowdley et.al. |
| Ecuador | 0.5 | 0.4 | 0.5 | Very Low | Country | Andean Latin America | Kowdley et.al. |
| Egypt | 4.2 | 1.9 | 6.5 | High-Intermediate | Country^β^ | North Africa/Middle East | Kowdley et.al. |
| El Salvador | 0.4 | 0.3 | 0.6 | Very Low | Country | Central Latin America | Kowdley et.al. |
| Equatorial Guinea | 11.4 | 8.5 | 14.4 | High | Regional | Middle Africa | Kowdley et.al. |
| Eritrea | 15.5 | 2.0 | 29.0 | High | Country | Eastern Sub-Saharan Africa | Kowdley et.al. |
| Estonia | 0.6 | 0.4 | 0.7 | Very Low | Country | Eastern Europe | Kowdley et.al. |
| Ethiopia | 5.5 | 2.6 | 8.4 | High | Country^β^ | Eastern Sub-Saharan Africa | Kowdley et.al. |
| Faeroe Islands (DK) | 0.6 | 0.3 | 0.7 | Very Low | Regional | Western Europe | Kowdley et.al. |
| Fiji | 5.8 | 3.7 | 7.9 | High-Intermediate | Country | Oceania | Kowdley et.al. |
| Finland | 0.2 | 0.1 | 0.4 | Very Low | Country | High Income North America | Hahné et. al. |
| Former Netherlands Antilles | 4.5 | 2.5 | 6.6 | High-Intermediate | Regional | Caribbean | Kowdley et.al. |
| Former Serbia and Montenegro | 3.3 | 2.3 | 4.2 | Low-Intermediate | Regional | Eastern Europe | Kowdley et.al. |
| Former USSR | 3.8 | 2.7 | 4.9 | Low-Intermediate | Country | Eastern Europe | Kowdley et.al. |
| France | 0.7 | 0.4 | 1.1 | Very Low | Country | Western Europe | Kowdley et.al. |
| Gabon | 11.4 | 8.5 | 14.4 | High | Regional | Central Sub-Saharan Africa | Kowdley et.al. |
| Gambia | 3.4 | 2.4 | 4.5 | Low-Intermediate | Regional | West Africa | Kowdley et.al. |
| Georgia | 3.9 | 1.3 | 6.5 | Low-Intermediate | Country | Central Asia | Kowdley et.al. |
| Germany | 0.6 | 0.4 | 0.8 | Very Low | Country | Western Europe | Kowdley et.al. |
| Ghana | 13.4 | 10.5 | 16.4 | High | Country | West Africa | Kowdley et.al. |
| Greece | 2.3 | 1.5 | 3.1 | Low-Intermediate | Country^β^ | Western Europe | Kowdley et.al. |
| Greenland (DK) | 0.6 | 0.3 | 0.7 | Very Low | Regional | Western Europe | Kowdley et.al. |
| Guatemala | 3.7 | 1.4 | 6.1 | Low-Intermediate | Country | Central Latin America | Kowdley et.al. |
| Guinea | 16.3 | 14.6 | 18.1 | High | Country | West Africa | Kowdley et.al. |
| Guinea-Bissau | 13.2 | 10.2 | 16.2 | High | Regional | Western Africa | Kowdley et.al. |
| Guyana | 1.3 | 0.7 | 1.9 | Low | Country | Caribbean | Kowdley et.al. |
| Haiti | 4.8 | 3.9 | 5.7 | High-Intermediate | Country | Caribbean | Kowdley et.al. |
| Honduras | 0.6 | 0.4 | 0.7 | Very Low | Country | Central Latin America | Kowdley et.al. |
| Hungary | 1.1 | 0.0 | 2.1 | Low | Country | Central Europe | Kowdley et.al. |
| Iceland | 0.6 | 0.3 | 0.7 | Very Low | Regional | Western Europe | Kowdley et.al. |
| India | 3.2 | 2.9 | 3.6 | Low-Intermediate | Country | South Asia | Kowdley et.al. |
| Indonesia | 3.9 | 3.1 | 4.8 | Low-Intermediate | Country | Southeast Asia | Kowdley et.al. |
| Iran | 3.1 | 2.7 | 3.5 | Low-Intermediate | Country | North Africa/Middle East | Kowdley et.al. |
| Iraq | 1.3 | 0.0 | 2.9 | Low | Country | North Africa/Middle East | Kowdley et.al. |
| Ireland | 0.1 | 0.0 | 0.3 | Very Low | Country | Western Europe | Hahné et. al. |
| Israel | 1.3 | 1.0 | 1.6 | Low | Country | Western Europe | Kowdley et.al. |
| Italy | 1.9 | 1.3 | 2.5 | Low-Intermediate | Country^β^ | Western Europe | Kowdley et.al. |
| Ivory Coast | 13.2 | 10.2 | 16.2 | High | Regional | Western Africa | Kowdley et.al. |
| Jamaica | 3.9 | 0.8 | 7.1 | Low-Intermediate | Country | Caribbean | Kowdley et.al. |
| Japan | 0.6 | 0.6 | 0.7 | Very Low | Country | Asia Pacific High Income | Kowdley et.al. |
| Jordan | 6.4 | 3.7 | 9.0 | High-Intermediate | Country | North Africa/Middle East | Kowdley et.al. |
| Kazakhstan | 5.0 | 3.3 | 6.6 | High-Intermediate | Country | Central Asia | Kowdley et.al. |
| Kenya | 5.7 | 4.2 | 7.2 | High-Intermediate | Country | Eastern Sub-Saharan Africa | Kowdley et.al. |
| Kosovo | 3.3 | 2.3 | 4.2 | Low-Intermediate | Regional | Central Europe | Kowdley et.al. |
| Kuwait | 3.9 | 2.0 | 5.7 | Low-Intermediate | Country | North Africa/Middle East | Kowdley et.al. |
| Kyrgyzstan | 3.6 | 3.1 | 4.2 | Low-Intermediate | Regional | South Central Asia | Kowdley et.al. |
| Laos | 13.6 | 11.6 | 15.6 | High | Country | Southeast Asia | Kowdley et.al. |
| Latvia | 1.4 | 1.1 | 1.7 | Low | Country | Eastern Europe | Kowdley et.al. |
| Lebanon | 1.7 | 0.9 | 2.5 | Low | Country | North Africa/Middle East | Kowdley et.al. |
| Liberia | 16.5 | 11.6 | 21.5 | High | Country | West Africa | Kowdley et.al. |
| Libya | 2.2 | 1.5 | 5.9 | High-Intermediate | Country | Northern Africa | Ezzikouri et. al. |
| Liechtenstein | 0.6 | 0.3 | 0.7 | Very Low | Regional | Western Europe | Kowdley et.al. |
| Lithuania | 2.0 | 1.4 | 2.7 | Low-Intermediate | Country | Eastern Europe | Kowdley et.al. |
| Luxembourg | 0.6 | 0.3 | 0.7 | Very Low | Regional | Western Europe | Kowdley et.al. |
| Macedonia | 3.3 | 2.3 | 4.2 | Low-Intermediate | Country | Central Europe | Kowdley et.al. |
| Madagascar | 9.7 | 7.1 | 12.3 | High | Regional | Eastern Africa | Kowdley et.al. |
| Malaysia | 5.6 | 4.3 | 6.9 | High-Intermediate | Country | Southeast Asia | Kowdley et.al. |
| Mali | 13.2 | 10.2 | 16.2 | High | Regional | Western Africa | Kowdley et.al. |
| Malta | 0.6 | 0.3 | 0.7 | Very Low | Regional | Western Europe | Kowdley et.al. |
| Mauritania | 13.2 | 10.2 | 16.2 | High | Regional | West Africa | Kowdley et.al. |
| Mauritius | 9.3 | 8.2 | 10.5 | High | Regional | Southeast Asia | Kowdley et.al. |
| Mexico | 0.5 | 0.3 | 0.7 | Very Low | Country | Central Latin America | Kowdley et.al. |
| Micronesia | 15.1 | 11.2 | 19.0 | High | Country | Oceania | Kowdley et.al. |
| Moldova | 9.6 | 6.9 | 12.3 | High | Country | Eastern Europe | Kowdley et.al. |
| Monaco | 0.6 | 0.3 | 0.7 | Very Low | Regional | Western Europe | Kowdley et.al. |
| Mongolia | 9.0 | 8.5 | 9.5 | High | Regional | East Asia | Kowdley et.al. |
| Montenegro | 3.3 | 2.3 | 4.2 | Low-Intermediate | Regional | Eastern Europe | Kowdley et.al. |
| Morocco | 1.8 | 1.5 | 5.9 | Low-Intermediate | Country | North Africa/Middle East | Ezzikouri et. al. |
| Mozambique | 9.7 | 7.1 | 12.3 | High | Regional | Eastern Africa | Kowdley et.al. |
| Myanmar | 11.6 | 9.5 | 13.7 | High | Country | Southeast Asia | Kowdley et.al. |
| Nepal | 2.3 | 1.7 | 2.9 | Low-Intermediate | Country | South Asia | Kowdley et.al. |
| Netherlands | 0.1 | 0.0 | 0.2 | Very Low | Country | Western Europe | Hahné et. al. |
| New Zealand | 0.5 | 0.4 | 0.6 | Very Low | Country | Australasia | Kowdley et.al. |
| Nicaragua | 0.7 | 0.0 | 1.8 | Very Low | Country | Central Latin America | Kowdley et.al. |
| Nigeria | 13.3 | 11.6 | 15.1 | High | Country | West Africa | Kowdley et.al. |
| Northern America | 0.7 | 0.6 | 0.9 | Very Low | Country | High Income North America | Kowdley et.al. |
| Norway | 0.6 | 0.3 | 0.7 | Very Low | Regional | Western Europe | Kowdley et.al. |
| Pakistan | 4.2 | 3.6 | 4.8 | High-Intermediate | Country | South Asia | Kowdley et.al. |
| Palestine | 6.6 | 4.6 | 8.6 | High-Intermediate | Regional | Northern Africa | Kowdley et.al. |
| Panama | 0.9 | 0.5 | 1.3 | Very Low | Country | Central Latin America | Kowdley et.al. |
| Paraguay | 0.8 | 0.5 | 1.1 | Very Low | Country | Tropical Latin America | Kowdley et.al. |
| Peru | 1.9 | 1.3 | 2.4 | Low | Country | Andean Latin America | Kowdley et.al. |
| Philippines | 7.4 | 6.3 | 8.4 | High-Intermediate | Country | Southeast Asia | Kowdley et.al. |
| Poland | 1.4 | 1.2 | 1.7 | Low | Country | Central Europe | Kowdley et.al. |
| Portugal | 1.4 | 0.7 | 2.0 | Low | Country | Western Europe | Kowdley et.al. |
| Republic of Korea | 4.3 | 3.9 | 4.7 | High-Intermediate | Country^β^ | East Asia | Kowdley et. al. |
| Romania | 5.5 | 5.2 | 5.7 | High-Intermediate | Country^β^ | Central Europe | Kowdley et. al. |
| Russia | 2.9 | 2.2 | 3.6 | Low-Intermediate | Country | Eastern Europe | Kowdley et.al. |
| Rwanda | 9.7 | 7.1 | 12.3 | High | Regional | Eastern Africa | Kowdley et.al. |
| Samoa | 4.3 | 2.5 | 6.1 | High-Intermediate | Country | Oceania | Kowdley et.al. |
| Sao Tome and Principe | 13.2 | 10.2 | 16.2 | High | Regional | Western Africa | Kowdley et.al. |
| Saudi Arabia | 5.3 | 3.8 | 6.9 | High-Intermediate | Country^β^ | North Africa/Middle East | Kowdley et.al. |
| Senegal | 12.7 | 10.1 | 15.2 | High | Country | West Africa | Kowdley et.al. |
| Serbia | 3.3 | 2.3 | 4.2 | Low-Intermediate | Regional | Eastern Europe | Kowdley et.al. |
| Sierra Leone | 11.9 | 6.5 | 17.3 | High | Country | West Africa | Kowdley et.al. |
| Singapore | 6.0 | 5.0 | 7.0 | High-Intermediate | Country | Asia Pacific High Income | Kowdley et.al. |
| Slovakia | 0.7 | 0.4 | 1.0 | Very Low | Country | Central Europe | Kowdley et.al. |
| Slovenia | 3.3 | 2.3 | 4.2 | Low-Intermediate | Regional | Eastern Europe | Kowdley et.al. |
| Somalia | 12.4 | 8.9 | 15.9 | High | Country | Eastern Sub-Saharan Africa | Kowdley et.al. |
| South Africa | 6.2 | 4.7 | 7.7 | High-Intermediate | Country | Southern Sub-Saharan Africa | Kowdley et.al. |
| South Korea | 4.3 | 3.9 | 4.7 | High-Intermediate | Country^β^ | Asia Pacific High Income | Kowdley et.al. |
| Spain | 0.7 | 0.3 | 1.0 | Low | Country^β^ | Western Europe | Kowdley et.al. |
| Sri Lanka | 2.4 | 0.0 | 5.5 | Low-Intermediate | Country | Southeast Asia | Kowdley et.al. |
| Sudan | 18.6 | 14.2 | 23.0 | High | Country | Eastern Sub-Saharan Africa | Kowdley et.al. |
| Suriname | 4.5 | 2.5 | 6.6 | High-Intermediate | Regional | Caribbean | Kowdley et.al. |
| Sweden | 0.2 | 0.1 | 0.4 | Very Low | Country | Western Europe | Hahné et al. |
| Switzerland | 0.6 | 0.3 | 0.7 | Very Low | Regional | Western Europe | Kowdley et.al. |
| Syria | 5.6 | 4.8 | 6.4 | High-Intermediate | Country | North Africa/Middle East | Kowdley et.al. |
| Taiwan | 10.2 | 9.4 | 11.1 | High | Country^β^ | East Asia | Kowdley et.al. |
| Tajikistan | 3.6 | 3.1 | 4.2 | Low-Intermediate | Regional | South Central Asia | Kowdley et.al. |
| Tanzania | 5.7 | 4.4 | 6.9 | High-Intermediate | Country | Eastern Sub-Saharan Africa | Kowdley et.al. |
| Timor-Leste | 3.9 | 3.1 | 4.8 | Low-Intermediate | Country^α^ | Southeast Asia | Kowdley et.al. |
| Thailand | 5.5 | 4.6 | 6.4 | High-Intermediate | Country^β^ | Southeast Asia | Kowdley et.al. |
| Togo | 13.2 | 10.2 | 16.2 | High | Regional | West Africa | Kowdley et.al. |
| Tonga | 13.0 | 7.6 | 18.4 | High | Country | Oceania | Kowdley et.al. |
| Trinidad and Tobago | 0.7 | 0.5 | 0.9 | Very Low | Country | Caribbean | Kowdley et.al. |
| Tunisia | 4.9 | 1.5 | 5.9 | High-Intermediate | Country | Northern Africa | Ezzikouri et. al. |
| Turkey | 4.3 | 3.7 | 4.9 | High-Intermediate | Country^β^ | North Africa/Middle East | Kowdley et.al. |
| Turkmenistan | 3.6 | 3.1 | 4.2 | Low-Intermediate | Regional | South Central Asia | Kowdley et.al. |
| Uganda | 10.3 | 8.5 | 12.0 | High | Country | Eastern Sub-Saharan Africa | Kowdley et.al. |
| Ukraine | 2.2 | 1.2 | 3.2 | Low-Intermediate | Country | Eastern Europe | Kowdley et.al. |
| United Kingdom | 0.5 | 0.3 | 0.6 | Very Low | Country | Western Europe | Kowdley et.al. |
| United States* | 0.3 | 0.2 | 0.3 | Very Low | Country | High Income North America | Ioannou et. al. |
| Uruguay | 0.4 | 0.3 | 0.5 | Very Low | Country | Southern Latin America | Kowdley et.al. |
| Uzbekistan | 6.3 | 4.2 | 8.5 | High-Intermediate | Country | Central Asia | Kowdley et.al. |
| Venezuela | 1.9 | 1.1 | 2.7 | Low | Country | Central Latin America | Kowdley et.al. |
| Vietnam | 12.5 | 11.5 | 13.5 | High | Country | Southeast Asia | Kowdley et.al. |
| Yemen | 13.2 | 10.4 | 16.1 | High | Country | North Africa/Middle East | Kowdley et.al. |
| Yugoslavia | 4.0 | 1.3 | 6.6 | Low-Intermediate | Country | Central Europe | Kowdley et.al. |
| Zambia^#^ | 9.7 | 7.1 | 12.3 | High | Regional | Eastern Africa | Kowdley et.al. |
| Zimbabwe | 13.9 | 10.7 | 17.1 | High | Country | Southern Sub-Saharan Africa | Kowdley et.al. |

Notes: All estimates are all ages aside from that marked with * denoting an age profile of sample >6 years old or ^#^ denoting an unknown age distribution. ^α^ Denotes an estimate for Indonesia given the geographical proximity. ^β^ Denotes a statistically significant decline over time and the consequent selection of the estimate from the period of 2000 onwards

# Annex 9 Chronic Hepatitis B burden - country tables: 50 largest migrant populations per EU/EEA country

The following country tables list the estimated number of Chronic Hepatitis B (CHB) cases among the 50 largest migrant populations residing in the individual EU/EEA host countries. The tables are sorted in descending order by the absolute number of CHB cases, and first list populations from high endemic countries (HBsAg prevalence ≥2%), followed by populations from low endemic countries.

Notes on dissolved states/former nation states listed:

China includes the territory of Hong Kong

Kosovo refers to the territory as per UN SCR 1244/99

Former Czechoslovakia refers to the nation state area of Czechoslovakia prior to 1993

Former Serbia and

Former Soviet Union refers to the nation state of the USSR prior to 1991

Former Yugoslavia refers to the nation state of Yugoslavia prior to 1992

Former Yugoslav Republic refers to the nation state (now Serbia and Montenegro) established between 1992 to 2006 (https://en.wikipedia.org/wiki/Serbia_and_Montenegro)

## Austria

| **Country** | **Population** | **HBsAg endemicity** | **HBsAg Prevalence** | | | **Estimated number of CHB cases** | | |
| --- | --- | --- | --- | --- | --- | --- | --- | --- |
|  |  |  | **%** | **LL 95%CI** | **UL 95%CI** | **Cases** | **Lower** | **Upper** |
| **Turkey** | 159,386 | int-high | 4.29 | 3.7 | 4.88 | 6,838 | 5,897 | 7,778 |
| **Bosnia and Herzegovina** | 151,580 | int-high | 3.63 | 2.26 | 5 | 5,502 | 3,426 | 7,579 |
| **Serbia** | 130,828 | int-high | 3.29 | 2.33 | 4.24 | 4,304 | 3,048 | 5,547 |
| **Romania** | 74,110 | int-high | 5.49 | 5.24 | 5.73 | 4,069 | 3,883 | 4,247 |
| **China** | 14,347 | int-high | 10.23 | 9.35 | 11.11 | 1,468 | 1,341 | 1,594 |
| **Afghanistan** | 13,150 | int-high | 10.46 | 5.85 | 15.07 | 1,375 | 769 | 1,982 |
| **Kosovo** | 28,033 | int-high | 3.29 | 2.33 | 4.24 | 922 | 653 | 1,189 |
| **Nigeria** | 6,800 | int-high | 13.31 | 11.57 | 15.06 | 905 | 787 | 1,024 |
| **Philippines** | 12,255 | int-high | 7.36 | 6.32 | 8.39 | 902 | 775 | 1,028 |
| **Russia** | 29,264 | int-high | 2.89 | 2.16 | 3.62 | 846 | 632 | 1,059 |
| **Bulgaria** | 17,106 | int-high | 4.25 | 2.8 | 5.7 | 727 | 479 | 975 |
| **FYR Macedonia** | 21,695 | int-high | 3.29 | 2.33 | 4.24 | 714 | 505 | 920 |
| **Slovenia** | 18,777 | int-high | 3.29 | 2.33 | 4.24 | 618 | 438 | 796 |
| **Egypt** | 12,891 | int-high | 4.18 | 1.85 | 6.51 | 539 | 238 | 839 |
| **Iran** | 14,640 | int-high | 3.1 | 2.69 | 3.5 | 454 | 394 | 512 |
| **India** | 12,242 | int-high | 3.23 | 2.92 | 3.55 | 395 | 357 | 435 |
| **Vietnam** | 2,866 | int-high | 12.48 | 11.46 | 13.5 | 358 | 328 | 387 |
| **Dominican Republic** | 2,859 | int-high | 10.68 | 5.89 | 15.46 | 305 | 168 | 442 |
| **Thailand** | 5,267 | int-high | 5.54 | 4.64 | 6.43 | 292 | 244 | 339 |
| **Ghana** | 2,057 | int-high | 13.44 | 10.5 | 16.38 | 276 | 216 | 337 |
| **Albania** | 2,964 | int-high | 9 | 8.1 | 9.8 | 267 | 240 | 290 |
| **Syria** | 4,050 | int-high | 5.62 | 4.82 | 6.42 | 228 | 195 | 260 |
| **Pakistan** | 5,178 | int-high | 4.17 | 3.59 | 4.75 | 216 | 186 | 246 |
| **Ukraine** | 8,769 | int-high | 2.2 | 1.15 | 3.24 | 193 | 101 | 284 |
| **Tunisia** | 3,437 | int-high | 4.9 | 1.51 | 5.89 | 168 | 52 | 202 |
| **South Africa** | 2,359 | int-high | 6.2 | 4.68 | 7.71 | 146 | 110 | 182 |
| **Bangladesh** | 2,504 | int-high | 4.83 | 4.02 | 5.64 | 121 | 101 | 141 |
| **Georgia** | 2,839 | int-high | 3.89 | 1.25 | 6.54 | 110 | 35 | 186 |
| **South Korea** | 2,289 | int-high | 4.33 | 3.94 | 4.73 | 99 | 90 | 108 |
| **Greece** | 4,231 | int-high | 2.33 | 1.54 | 3.11 | 99 | 65 | 132 |
| **Germany** | 205,986 | low | 0.6 | 0.4 | 0.8 | 1,236 | 824 | 1,648 |
| **Poland** | 62,888 | low | 1.44 | 1.16 | 1.72 | 906 | 730 | 1,082 |
| **Croatia** | 39,037 | low | 1.47 | 0.84 | 2.1 | 574 | 328 | 820 |
| **Hungary** | 47,551 | low | 1.08 | 0.04 | 2.11 | 514 | 19 | 1,003 |
| **Italy** | 26,117 | low | 1.89 | 1.26 | 2.52 | 494 | 329 | 658 |
| **Czech Republic** | 41,679 | low | 0.7 | 0.43 | 0.98 | 292 | 179 | 408 |
| **Slovakia** | 29,828 | low | 0.7 | 0.43 | 0.98 | 209 | 128 | 292 |
| **Brazil** | 4,617 | low | 1.78 | 1.54 | 2.02 | 82 | 71 | 93 |
| **Switzerland** | 14,039 | low | 0.55 | 0.34 | 0.71 | 77 | 48 | 100 |
| **Iraq** | 5,314 | low | 1.31 | 0 | 2.87 | 70 | 0 | 153 |
| **France** | 7,865 | low | 0.68 | 0.44 | 1.05 | 53 | 35 | 83 |
| **United Kingdom** | 9,735 | low | 0.54 | 0.3 | 0.6 | 53 | 29 | 58 |
| **Spain** | 4,728 | low | 0.66 | 0.34 | 0.97 | 31 | 16 | 46 |
| **United States** | 10,066 | low | 0.27 | 0.2 | 0.34 | 27 | 20 | 34 |
| **Belgium** | 2,455 | low | 0.7 | 0.4 | 1.2 | 17 | 10 | 29 |
| **Japan** | 2,530 | low | 0.63 | 0.6 | 0.7 | 16 | 15 | 18 |
| **Canada** | 2,242 | low | 0.7 | 0.6 | 0.9 | 16 | 13 | 20 |
| **Armenia** | 2,753 | low | 0.57 | 0.19 | 0.94 | 16 | 5 | 26 |
| **Netherlands** | 7,826 | low | 0.1 | 0 | 0.2 | 8 | 0 | 16 |
| **Sweden** | 2,916 | low | 0.2 | 0.1 | 0.4 | 6 | 3 | 12 |

## Belgium

| **Country** | **Population** | **HBsAg endemicity** | **HBsAg Prevalence** | | | **Estimated number of CHB cases** | | |
| --- | --- | --- | --- | --- | --- | --- | --- | --- |
|  |  |  | **%** | **LL 95%CI** | **UL 95%CI** | **Cases** | **Lower** | **Upper** |
| **DRC Congo** | 84,278 | int-high | 11.44 | 8.46 | 14.43 | 9,641 | 7,130 | 12,161 |
| **Turkey** | 99,011 | int-high | 4.29 | 3.7 | 4.88 | 4,248 | 3,663 | 4,832 |
| **Romania** | 53,087 | int-high | 5.49 | 5.24 | 5.73 | 2,914 | 2,782 | 3,042 |
| **Former Soviet Union** | 54,604 | int-high | 3.83 | 2.74 | 4.91 | 2,091 | 1,496 | 2,681 |
| **Guinea** | 12,702 | int-high | 16.33 | 14.61 | 18.05 | 2,074 | 1,856 | 2,293 |
| **Former Yugoslavia** | 47,923 | int-high | 3.98 | 1.32 | 6.64 | 1,907 | 633 | 3,182 |
| **China** | 18,421 | int-high | 10.23 | 9.35 | 11.11 | 1,884 | 1,722 | 2,047 |
| **Cameroon** | 14,760 | int-high | 11.44 | 8.46 | 14.43 | 1,689 | 1,249 | 2,130 |
| **Rwanda** | 14,027 | int-high | 9.66 | 7.06 | 12.26 | 1,355 | 990 | 1,720 |
| **Afghanistan** | 12,256 | int-high | 10.46 | 5.85 | 15.07 | 1,282 | 717 | 1,847 |
| **Ghana** | 8,203 | int-high | 13.44 | 10.5 | 16.38 | 1,102 | 861 | 1,344 |
| **Bulgaria** | 24,208 | int-high | 4.25 | 2.8 | 5.7 | 1,029 | 678 | 1,380 |
| **Vietnam** | 8,177 | int-high | 12.48 | 11.46 | 13.5 | 1,020 | 937 | 1,104 |
| **Albania** | 9,470 | int-high | 9 | 8.1 | 9.8 | 852 | 767 | 928 |
| **Côte d'Ivoire** | 5,588 | int-high | 13.17 | 10.18 | 16.17 | 736 | 569 | 904 |
| **Angola** | 6,426 | int-high | 11.44 | 8.46 | 14.43 | 735 | 544 | 927 |
| **Philippines** | 9,819 | int-high | 7.36 | 6.32 | 8.39 | 723 | 621 | 824 |
| **Togo** | 5,455 | int-high | 13.17 | 10.18 | 16.17 | 718 | 555 | 882 |
| **Nigeria** | 5,307 | int-high | 13.31 | 11.57 | 15.06 | 706 | 614 | 799 |
| **Algeria** | 25,719 | int-high | 2.6 | 0 | 12.19 | 669 | 0 | 3,135 |
| **Burundi** | 6,836 | int-high | 9.66 | 7.06 | 12.26 | 660 | 483 | 838 |
| **Tunisia** | 13,309 | int-high | 4.9 | 1.51 | 5.89 | 652 | 201 | 784 |
| **Senegal** | 5,104 | int-high | 12.66 | 10.14 | 15.18 | 646 | 518 | 775 |
| **India** | 18,561 | int-high | 3.23 | 2.92 | 3.55 | 600 | 542 | 659 |
| **Pakistan** | 12,703 | int-high | 4.17 | 3.59 | 4.75 | 530 | 456 | 603 |
| **Congo** | 4,337 | int-high | 11.44 | 8.46 | 14.43 | 496 | 367 | 626 |
| **Thailand** | 8,278 | int-high | 5.54 | 4.64 | 6.43 | 459 | 384 | 532 |
| **Syria** | 7,341 | int-high | 5.62 | 4.82 | 6.42 | 413 | 354 | 471 |
| **Greece** | 15,356 | int-high | 2.33 | 1.54 | 3.11 | 358 | 236 | 478 |
| **Iran** | 10,940 | int-high | 3.1 | 2.69 | 3.5 | 339 | 294 | 383 |
| **Morocco** | 201,921 | low | 1.8 | 1.51 | 5.89 | 3,635 | 3,049 | 11,893 |
| **Italy** | 119,714 | low | 1.89 | 1.26 | 2.52 | 2,263 | 1,508 | 3,017 |
| **France** | 180,429 | low | 0.68 | 0.44 | 1.05 | 1,227 | 794 | 1,895 |
| **Poland** | 68,021 | low | 1.44 | 1.16 | 1.72 | 980 | 789 | 1,170 |
| **Germany** | 83,368 | low | 0.6 | 0.4 | 0.8 | 500 | 333 | 667 |
| **Portugal** | 31,564 | low | 1.35 | 0.66 | 2.04 | 426 | 208 | 644 |
| **Spain** | 42,892 | low | 0.66 | 0.34 | 0.97 | 283 | 146 | 416 |
| **Brazil** | 12,006 | low | 1.78 | 1.54 | 2.02 | 214 | 185 | 243 |
| **Iraq** | 10,951 | low | 1.31 | 0 | 2.87 | 143 | 0 | 314 |
| **Netherlands** | 127,562 | low | 0.1 | 0 | 0.2 | 128 | 0 | 255 |
| **United Kingdom** | 23,138 | low | 0.54 | 0.3 | 0.6 | 125 | 69 | 139 |
| **Lebanon** | 6,272 | low | 1.66 | 0.86 | 2.46 | 104 | 54 | 154 |
| **Hungary** | 7,904 | low | 1.08 | 0.04 | 2.11 | 85 | 3 | 167 |
| **Luxembourg** | 12,982 | low | 0.55 | 0.34 | 0.71 | 71 | 44 | 92 |
| **Colombia** | 5,799 | low | 1.2 | 0.27 | 2.13 | 70 | 16 | 124 |
| **Former Czechoslovakia** | 8,221 | low | 0.7 | 0.43 | 0.98 | 58 | 35 | 81 |
| **United States** | 15,999 | low | 0.27 | 0.2 | 0.34 | 43 | 32 | 54 |
| **Canada** | 4,957 | low | 0.7 | 0.6 | 0.9 | 35 | 30 | 45 |
| **Switzerland** | 5,042 | low | 0.55 | 0.34 | 0.71 | 28 | 17 | 36 |
| **Ecuador** | 5,900 | low | 0.47 | 0.42 | 0.51 | 28 | 25 | 30 |

## Bulgaria

| **Country** | **Population** | **HBsAg endemicity** | **HBsAg Prevalence** | | | **Estimated number of CHB cases** | | |
| --- | --- | --- | --- | --- | --- | --- | --- | --- |
|  |  |  | **%** | **LL 95%CI** | **UL 95%CI** | **Cases** | **Lower** | **Upper** |
| **Russia** | 19,533 | int-high | 2.89 | 2.16 | 3.62 | 565 | 422 | 707 |
| **Romania** | 5,380 | int-high | 5.49 | 5.24 | 5.73 | 295 | 282 | 308 |
| **Turkey** | 6,227 | int-high | 4.29 | 3.7 | 4.88 | 267 | 230 | 304 |
| **Moldova** | 1,996 | int-high | 9.61 | 6.92 | 12.29 | 192 | 138 | 245 |
| **Greece** | 7,377 | int-high | 2.33 | 1.54 | 3.11 | 172 | 114 | 229 |
| **Ukraine** | 6,084 | int-high | 2.2 | 1.15 | 3.24 | 134 | 70 | 197 |
| **Albania** | 1,078 | int-high | 9 | 8.1 | 9.8 | 97 | 87 | 106 |
| **China** | 929 | int-high | 10.23 | 9.35 | 11.11 | 95 | 87 | 103 |
| **FYR Macedonia** | 2,384 | int-high | 3.29 | 2.33 | 4.24 | 78 | 56 | 101 |
| **Vietnam** | 596 | int-high | 12.48 | 11.46 | 13.5 | 74 | 68 | 80 |
| **Serbia** | 2,246 | int-high | 3.29 | 2.33 | 4.24 | 74 | 52 | 95 |
| **Syria** | 1,298 | int-high | 5.62 | 4.82 | 6.42 | 73 | 63 | 83 |
| **Azerbaijan** | 1,871 | int-high | 3.11 | 1.39 | 4.84 | 58 | 26 | 91 |
| **Kazakhstan** | 1,067 | int-high | 4.95 | 3.34 | 6.56 | 53 | 36 | 70 |
| **Uzbekistan** | 717 | int-high | 6.34 | 4.22 | 8.46 | 45 | 30 | 61 |
| **Afghanistan** | 306 | int-high | 10.46 | 5.85 | 15.07 | 32 | 18 | 46 |
| **Bangladesh** | 547 | int-high | 4.83 | 4.02 | 5.64 | 26 | 22 | 31 |
| **Belarus** | 672 | int-high | 3.19 | 1.72 | 4.65 | 21 | 12 | 31 |
| **Kosovo** | 399 | int-high | 3.29 | 2.33 | 4.24 | 13 | 9 | 17 |
| **Georgia** | 319 | int-high | 3.89 | 1.25 | 6.54 | 12 | 4 | 21 |
| **Jordan** | 183 | int-high | 6.36 | 3.72 | 9 | 12 | 7 | 16 |
| **Turkmenistan** | 269 | int-high | 3.61 | 3.05 | 4.19 | 10 | 8 | 11 |
| **Algeria** | 351 | int-high | 2.6 | 0 | 12.19 | 9 | 0 | 43 |
| **Iran** | 268 | int-high | 3.1 | 2.69 | 3.5 | 8 | 7 | 9 |
| **Egypt** | 153 | int-high | 4.18 | 1.85 | 6.51 | 6 | 3 | 10 |
| **India** | 178 | int-high | 3.23 | 2.92 | 3.55 | 6 | 5 | 6 |
| **Libya** | 196 | int-high | 2.2 | 1.51 | 5.89 | 4 | 3 | 12 |
| **Lithuania** | 131 | int-high | 2.03 | 1.37 | 2.69 | 3 | 2 | 4 |
| **Italy** | 2,261 | low | 1.89 | 1.26 | 2.52 | 43 | 28 | 57 |
| **United Kingdom** | 5,066 | low | 0.54 | 0.3 | 0.6 | 27 | 15 | 30 |
| **Spain** | 4,065 | low | 0.66 | 0.34 | 0.97 | 27 | 14 | 39 |
| **Germany** | 3,638 | low | 0.6 | 0.4 | 0.8 | 22 | 15 | 29 |
| **Poland** | 1,443 | low | 1.44 | 1.16 | 1.72 | 21 | 17 | 25 |
| **Iraq** | 825 | low | 1.31 | 0 | 2.87 | 11 | 0 | 24 |
| **Lebanon** | 637 | low | 1.66 | 0.86 | 2.46 | 11 | 5 | 16 |
| **France** | 1,255 | low | 0.68 | 0.44 | 1.05 | 9 | 6 | 13 |
| **Armenia** | 1,422 | low | 0.57 | 0.19 | 0.94 | 8 | 3 | 13 |
| **Czech Republic** | 1,028 | low | 0.7 | 0.43 | 0.98 | 7 | 4 | 10 |
| **Belgium** | 1,009 | low | 0.7 | 0.4 | 1.2 | 7 | 4 | 12 |
| **Cyprus** | 679 | low | 0.9 | 0.3 | 2 | 6 | 2 | 14 |
| **United States** | 2,023 | low | 0.27 | 0.2 | 0.34 | 5 | 4 | 7 |
| **Israel** | 413 | low | 1.26 | 0.97 | 1.55 | 5 | 4 | 6 |
| **Hungary** | 348 | low | 1.08 | 0.04 | 2.11 | 4 | 0 | 7 |
| **Austria** | 493 | low | 0.55 | 0.34 | 0.71 | 3 | 2 | 4 |
| **Cuba** | 257 | low | 0.86 | 0.58 | 1.14 | 2 | 1 | 3 |
| **Latvia** | 151 | low | 1.39 | 1.1 | 1.67 | 2 | 2 | 3 |
| **Slovakia** | 299 | low | 0.7 | 0.43 | 0.98 | 2 | 1 | 3 |
| **Australia** | 188 | low | 0.87 | 0.39 | 1.35 | 2 | 1 | 3 |
| **Netherlands** | 735 | low | 0.1 | 0 | 0.2 | 1 | 0 | 1 |

## Croatia

| **Country** | **Population** | **HBsAg endemicity** | **HBsAg Prevalence** | | | **Estimated number of CHB cases** | | |
| --- | --- | --- | --- | --- | --- | --- | --- | --- |
|  |  |  | **%** | **LL 95%CI** | **UL 95%CI** | **Cases** | **Lower** | **Upper** |
| **Bosnia and Herzegovina** | 409,357 | int-high | 3.63 | 2.26 | 5 | 14,860 | 9,251 | 20,468 |
| **Serbia** | 52,763 | int-high | 3.29 | 2.33 | 4.24 | 1,736 | 1,229 | 2,237 |
| **Kosovo** | 20,347 | int-high | 3.29 | 2.33 | 4.24 | 669 | 474 | 863 |
| **Slovenia** | 19,803 | int-high | 3.29 | 2.33 | 4.24 | 652 | 461 | 840 |
| **FYR Macedonia** | 10,167 | int-high | 3.29 | 2.33 | 4.24 | 334 | 237 | 431 |
| **Montenegro** | 6,249 | int-high | 3.29 | 2.33 | 4.24 | 206 | 146 | 265 |
| **China** | 451 | int-high | 10.23 | 9.35 | 11.11 | 46 | 42 | 50 |
| **Russian Federation** | 1,326 | int-high | 2.89 | 2.16 | 3.62 | 38 | 29 | 48 |
| **Romania** | 505 | int-high | 5.49 | 5.24 | 5.73 | 28 | 26 | 29 |
| **Ukraine** | 766 | int-high | 2.2 | 1.15 | 3.24 | 17 | 9 | 25 |
| **Albania** | 175 | int-high | 9 | 8.1 | 9.8 | 16 | 14 | 17 |
| **Bulgaria** | 308 | int-high | 4.25 | 2.8 | 5.7 | 13 | 9 | 18 |
| **South Africa** | 206 | int-high | 6.2 | 4.68 | 7.71 | 13 | 10 | 16 |
| **Moldova** | 115 | int-high | 9.61 | 6.92 | 12.29 | 11 | 8 | 14 |
| **Egypt** | 249 | int-high | 4.18 | 1.85 | 6.51 | 10 | 5 | 16 |
| **Philippines** | 76 | int-high | 7.36 | 6.32 | 8.39 | 6 | 5 | 6 |
| **Turkey** | 86 | int-high | 4.29 | 3.7 | 4.88 | 4 | 3 | 4 |
| **Greece** | 149 | int-high | 2.33 | 1.54 | 3.11 | 3 | 2 | 5 |
| **Kazakhstan** | 68 | int-high | 4.95 | 3.34 | 6.56 | 3 | 2 | 4 |
| **Libya** | 152 | int-high | 2.2 | 1.51 | 5.89 | 3 | 2 | 9 |
| **Belarus** | 95 | int-high | 3.19 | 1.72 | 4.65 | 3 | 2 | 4 |
| **Algeria** | 57 | int-high | 2.6 | 0 | 12.19 | 1 | 0 | 7 |
| **Germany** | 34,148 | low | 0.6 | 0.4 | 0.8 | 205 | 137 | 273 |
| **Italy** | 3,175 | low | 1.89 | 1.26 | 2.52 | 60 | 40 | 80 |
| **Austria** | 5,204 | low | 0.55 | 0.34 | 0.71 | 29 | 18 | 37 |
| **Switzerland** | 3,042 | low | 0.55 | 0.34 | 0.71 | 17 | 10 | 22 |
| **Australia** | 1,613 | low | 0.87 | 0.39 | 1.35 | 14 | 6 | 22 |
| **France** | 1,780 | low | 0.68 | 0.44 | 1.05 | 12 | 8 | 19 |
| **Hungary** | 1,055 | low | 1.08 | 0.04 | 2.11 | 11 | 0 | 22 |
| **Poland** | 745 | low | 1.44 | 1.16 | 1.72 | 11 | 9 | 13 |
| **Canada** | 1,015 | low | 0.7 | 0.6 | 0.9 | 7 | 6 | 9 |
| **Czech Republic** | 959 | low | 0.7 | 0.43 | 0.98 | 7 | 4 | 9 |
| **Slovakia** | 651 | low | 0.7 | 0.43 | 0.98 | 5 | 3 | 6 |
| **United States of America** | 1,635 | low | 0.27 | 0.2 | 0.34 | 4 | 3 | 6 |
| **United Kingdom** | 678 | low | 0.54 | 0.3 | 0.6 | 4 | 2 | 4 |
| **Brazil** | 195 | low | 1.78 | 1.54 | 2.02 | 3 | 3 | 4 |
| **Belgium** | 380 | low | 0.7 | 0.4 | 1.2 | 3 | 2 | 5 |
| **Argentina** | 202 | low | 1 | 0.39 | 1.62 | 2 | 1 | 3 |
| **Peru** | 86 | low | 1.85 | 1.29 | 2.42 | 2 | 1 | 2 |
| **Sweden** | 635 | low | 0.2 | 0.1 | 0.4 | 1 | 1 | 3 |
| **Israel** | 100 | low | 1.26 | 0.97 | 1.55 | 1 | 1 | 2 |
| **Spain** | 190 | low | 0.66 | 0.34 | 0.97 | 1 | 1 | 2 |
| **Lebanon** | 66 | low | 1.66 | 0.86 | 2.46 | 1 | 1 | 2 |
| **Iraq** | 82 | low | 1.31 | 0 | 2.87 | 1 | 0 | 2 |
| **Netherlands** | 818 | low | 0.1 | 0 | 0.2 | 1 | 0 | 2 |
| **New Zealand** | 117 | low | 0.5 | 0.42 | 0.56 | 1 | 0 | 1 |
| **Denmark** | 103 | low | 0.55 | 0.34 | 0.71 | 1 | 0 | 1 |
| **Chile** | 127 | low | 0.4 | 0.11 | 0.69 | 1 | 0 | 1 |

## Cyprus

| **Country** | **Population** | **HBsAg endemicity** | **HBsAg Prevalence** | | | **Estimated number of CHB cases** | | |
| --- | --- | --- | --- | --- | --- | --- | --- | --- |
|  |  |  | **%** | **LL 95%CI** | **UL 95%CI** | **Cases** | **Lower** | **Upper** |
| **Romania** | 24,532 | int-high | 5.49 | 5.24 | 5.73 | 1,347 | 1,285 | 1,406 |
| **Vietnam** | 7,016 | int-high | 12.48 | 11.46 | 13.5 | 876 | 804 | 947 |
| **Bulgaria** | 19,284 | int-high | 4.25 | 2.8 | 5.7 | 820 | 540 | 1,099 |
| **Philippines** | 10,009 | int-high | 7.36 | 6.32 | 8.39 | 737 | 633 | 840 |
| **Georgia** | 11,814 | int-high | 3.89 | 1.25 | 6.54 | 460 | 148 | 773 |
| **Greece** | 18,788 | int-high | 2.33 | 1.54 | 3.11 | 438 | 289 | 584 |
| **Russia** | 10,520 | int-high | 2.89 | 2.16 | 3.62 | 304 | 227 | 381 |
| **Moldova** | 2,348 | int-high | 9.61 | 6.92 | 12.29 | 226 | 162 | 289 |
| **Syria** | 3,272 | int-high | 5.62 | 4.82 | 6.42 | 184 | 158 | 210 |
| **Sri Lanka** | 7,327 | int-high | 2.41 | 0 | 5.53 | 177 | 0 | 405 |
| **South Africa** | 2,509 | int-high | 6.2 | 4.68 | 7.71 | 156 | 117 | 193 |
| **Egypt** | 3,292 | int-high | 4.18 | 1.85 | 6.51 | 138 | 61 | 214 |
| **China** | 1,099 | int-high | 10.23 | 9.35 | 11.11 | 112 | 103 | 122 |
| **India** | 2,996 | int-high | 3.23 | 2.92 | 3.55 | 97 | 87 | 106 |
| **Bangladesh** | 1,831 | int-high | 4.83 | 4.02 | 5.64 | 88 | 74 | 103 |
| **Ukraine** | 3,794 | int-high | 2.2 | 1.15 | 3.24 | 83 | 44 | 123 |
| **Pakistan** | 1,533 | int-high | 4.17 | 3.59 | 4.75 | 64 | 55 | 73 |
| **Nigeria** | 417 | int-high | 13.31 | 11.57 | 15.06 | 56 | 48 | 63 |
| **Sudan** | 294 | int-high | 18.59 | 14.22 | 22.96 | 55 | 42 | 68 |
| **Zimbabwe** | 343 | int-high | 13.91 | 10.7 | 17.11 | 48 | 37 | 59 |
| **Palestinian Territory** | 664 | int-high | 6.62 | 4.6 | 8.64 | 44 | 31 | 57 |
| **Iran** | 1,364 | int-high | 3.1 | 2.69 | 3.5 | 42 | 37 | 48 |
| **Congo** | 320 | int-high | 11.44 | 8.46 | 14.43 | 37 | 27 | 46 |
| **Serbia** | 1,009 | int-high | 3.29 | 2.33 | 4.24 | 33 | 24 | 43 |
| **Cameroon** | 287 | int-high | 11.44 | 8.46 | 14.43 | 33 | 24 | 41 |
| **Jordan** | 411 | int-high | 6.36 | 3.72 | 9 | 26 | 15 | 37 |
| **Albania** | 275 | int-high | 9 | 8.1 | 9.8 | 25 | 22 | 27 |
| **Belarus** | 675 | int-high | 3.19 | 1.72 | 4.65 | 22 | 12 | 31 |
| **Turkey** | 501 | int-high | 4.29 | 3.7 | 4.88 | 21 | 19 | 24 |
| **Lithuania** | 693 | int-high | 2.03 | 1.37 | 2.69 | 14 | 9 | 19 |
| **Nepal** | 472 | int-high | 2.32 | 1.71 | 2.93 | 11 | 8 | 14 |
| **United Kingdom** | 31,495 | low | 0.54 | 0.3 | 0.6 | 170 | 94 | 189 |
| **Poland** | 2,922 | low | 1.44 | 1.16 | 1.72 | 42 | 34 | 50 |
| **Lebanon** | 1,597 | low | 1.66 | 0.86 | 2.46 | 27 | 14 | 39 |
| **Iraq** | 1,946 | low | 1.31 | 0 | 2.87 | 25 | 0 | 56 |
| **Australia** | 1,576 | low | 0.87 | 0.39 | 1.35 | 14 | 6 | 21 |
| **Latvia** | 916 | low | 1.39 | 1.1 | 1.67 | 13 | 10 | 15 |
| **Germany** | 1,895 | low | 0.6 | 0.4 | 0.8 | 11 | 8 | 15 |
| **Italy** | 405 | low | 1.89 | 1.26 | 2.52 | 8 | 5 | 10 |
| **Slovakia** | 973 | low | 0.7 | 0.43 | 0.98 | 7 | 4 | 10 |
| **Hungary** | 620 | low | 1.08 | 0.04 | 2.11 | 7 | 0 | 13 |
| **Armenia** | 1,143 | low | 0.57 | 0.19 | 0.94 | 7 | 2 | 11 |
| **United States** | 2,021 | low | 0.27 | 0.2 | 0.34 | 5 | 4 | 7 |
| **Canada** | 777 | low | 0.7 | 0.6 | 0.9 | 5 | 5 | 7 |
| **France** | 644 | low | 0.68 | 0.44 | 1.05 | 4 | 3 | 7 |
| **Czech Republic** | 457 | low | 0.7 | 0.43 | 0.98 | 3 | 2 | 4 |
| **Sweden** | 571 | low | 0.2 | 0.1 | 0.4 | 1 | 1 | 2 |
| **Ireland** | 542 | low | 0.1 | 0 | 0.3 | 1 | 0 | 2 |
| **Netherlands** | 379 | low | 0.1 | 0 | 0.2 | 0 | 0 | 1 |

## Czech Republic

| **Country** | **Population** | **HBsAg endemicity** | **HBsAg Prevalence** | | | **Estimated number of CHB cases** | | |
| --- | --- | --- | --- | --- | --- | --- | --- | --- |
|  |  |  | **%** | **LL 95%CI** | **UL 95%CI** | **Cases** | **Lower** | **Upper** |
| **Vietnam** | 46,200 | int-high | 12.48 | 11.46 | 13.5 | 5,766 | 5,295 | 6,237 |
| **Ukraine** | 99,835 | int-high | 2.2 | 1.15 | 3.24 | 2,196 | 1,148 | 3,235 |
| **Russia** | 29,204 | int-high | 2.89 | 2.16 | 3.62 | 844 | 631 | 1,057 |
| **Moldova** | 6,285 | int-high | 9.61 | 6.92 | 12.29 | 604 | 435 | 772 |
| **China** | 4,512 | int-high | 10.23 | 9.35 | 11.11 | 462 | 422 | 501 |
| **Mongolia** | 4,383 | int-high | 8.97 | 8.47 | 9.48 | 393 | 371 | 416 |
| **Bulgaria** | 7,686 | int-high | 4.25 | 2.8 | 5.7 | 327 | 215 | 438 |
| **Kazakhstan** | 5,652 | int-high | 4.95 | 3.34 | 6.56 | 280 | 189 | 371 |
| **Romania** | 5,018 | int-high | 5.49 | 5.24 | 5.73 | 275 | 263 | 288 |
| **Uzbekistan** | 1,983 | int-high | 6.34 | 4.22 | 8.46 | 126 | 84 | 168 |
| **Belarus** | 3,895 | int-high | 3.19 | 1.72 | 4.65 | 124 | 67 | 181 |
| **Bosnia and Herzegovina** | 2,467 | int-high | 3.63 | 2.26 | 5 | 90 | 56 | 123 |
| **Former Yugoslavia** | 2,054 | int-high | 3.98 | 1.32 | 6.64 | 82 | 27 | 136 |
| **Nigeria** | 568 | int-high | 13.31 | 11.57 | 15.06 | 76 | 66 | 86 |
| **FYR Macedonia** | 1,712 | int-high | 3.29 | 2.33 | 4.24 | 56 | 40 | 73 |
| **South Korea** | 1,120 | int-high | 4.33 | 3.94 | 4.73 | 48 | 44 | 53 |
| **Turkey** | 1,114 | int-high | 4.29 | 3.7 | 4.88 | 48 | 41 | 54 |
| **India** | 1,184 | int-high | 3.23 | 2.92 | 3.55 | 38 | 35 | 42 |
| **Georgia** | 976 | int-high | 3.89 | 1.25 | 6.54 | 38 | 12 | 64 |
| **Thailand** | 678 | int-high | 5.54 | 4.64 | 6.43 | 38 | 31 | 44 |
| **Syria** | 658 | int-high | 5.62 | 4.82 | 6.42 | 37 | 32 | 42 |
| **Serbia** | 1,060 | int-high | 3.29 | 2.33 | 4.24 | 35 | 25 | 45 |
| **Tunisia** | 689 | int-high | 4.9 | 1.51 | 5.89 | 34 | 10 | 41 |
| **Azerbaijan** | 954 | int-high | 3.11 | 1.39 | 4.84 | 30 | 13 | 46 |
| **Kyrgyzstan** | 738 | int-high | 3.61 | 3.05 | 4.19 | 27 | 23 | 31 |
| **Former Yugoslav Republic** | 785 | int-high | 3.29 | 2.33 | 4.24 | 26 | 18 | 33 |
| **Egypt** | 602 | int-high | 4.18 | 1.85 | 6.51 | 25 | 11 | 39 |
| **Pakistan** | 477 | int-high | 4.17 | 3.59 | 4.75 | 20 | 17 | 23 |
| **Algeria** | 659 | int-high | 2.6 | 0 | 12.19 | 17 | 0 | 80 |
| **Greece** | 586 | int-high | 2.33 | 1.54 | 3.11 | 14 | 9 | 18 |
| **Lithuania** | 557 | int-high | 2.03 | 1.37 | 2.69 | 11 | 8 | 15 |
| **Slovakia** | 80,266 | low | 0.7 | 0.43 | 0.98 | 562 | 345 | 787 |
| **Poland** | 18,379 | low | 1.44 | 1.16 | 1.72 | 265 | 213 | 316 |
| **Germany** | 13,635 | low | 0.6 | 0.4 | 0.8 | 82 | 55 | 109 |
| **Italy** | 2,828 | low | 1.89 | 1.26 | 2.52 | 53 | 36 | 71 |
| **UK** | 4,508 | low | 0.54 | 0.3 | 0.6 | 24 | 14 | 27 |
| **Croatia** | 1,390 | low | 1.47 | 0.84 | 2.1 | 20 | 12 | 29 |
| **France** | 2,539 | low | 0.68 | 0.44 | 1.05 | 17 | 11 | 27 |
| **Austria** | 2,500 | low | 0.55 | 0.34 | 0.71 | 14 | 9 | 18 |
| **Armenia** | 1,939 | low | 0.57 | 0.19 | 0.94 | 11 | 4 | 18 |
| **Hungary** | 1,017 | low | 1.08 | 0.04 | 2.11 | 11 | 0 | 21 |
| **United States** | 3,840 | low | 0.27 | 0.2 | 0.34 | 10 | 8 | 13 |
| **Japan** | 1,266 | low | 0.63 | 0.6 | 0.7 | 8 | 8 | 9 |
| **Iraq** | 474 | low | 1.31 | 0 | 2.87 | 6 | 0 | 14 |
| **Spain** | 761 | low | 0.66 | 0.34 | 0.97 | 5 | 3 | 7 |
| **Canada** | 629 | low | 0.7 | 0.6 | 0.9 | 4 | 4 | 6 |
| **Belgium** | 554 | low | 0.7 | 0.4 | 1.2 | 4 | 2 | 7 |
| **Switzerland** | 490 | low | 0.55 | 0.34 | 0.71 | 3 | 2 | 3 |
| **Netherlands** | 2,451 | low | 0.1 | 0 | 0.2 | 2 | 0 | 5 |
| **Ireland** | 539 | low | 0.1 | 0 | 0.3 | 1 | 0 | 2 |

## Denmark

| **Country** | **Population** | **HBsAg endemicity** | **HBsAg Prevalence** | | | **Estimated number of CHB cases** | | |
| --- | --- | --- | --- | --- | --- | --- | --- | --- |
|  |  |  | **%** | **LL 95%CI** | **UL 95%CI** | **Cases** | **Lower** | **Upper** |
| **Turkey** | 32,066 | int-high | 4.29 | 3.7 | 4.88 | 1,376 | 1,186 | 1,565 |
| **Somalia** | 9,963 | int-high | 12.4 | 8.89 | 15.92 | 1,235 | 886 | 1,586 |
| **Vietnam** | 9,716 | int-high | 12.48 | 11.46 | 13.5 | 1,213 | 1,113 | 1,312 |
| **Afghanistan** | 11,435 | int-high | 10.46 | 5.85 | 15.07 | 1,196 | 669 | 1,723 |
| **China** | 10,877 | int-high | 10.23 | 9.35 | 11.11 | 1,113 | 1,017 | 1,208 |
| **Philippines** | 10,151 | int-high | 7.36 | 6.32 | 8.39 | 747 | 642 | 852 |
| **Romania** | 13,057 | int-high | 5.49 | 5.24 | 5.73 | 717 | 684 | 748 |
| **Thailand** | 11,551 | int-high | 5.54 | 4.64 | 6.43 | 640 | 536 | 743 |
| **Bosnia and Herzegovina** | 17,474 | int-high | 3.63 | 2.26 | 5 | 634 | 395 | 874 |
| **Pakistan** | 12,166 | int-high | 4.17 | 3.59 | 4.75 | 507 | 437 | 578 |
| **Iran** | 13,164 | int-high | 3.1 | 2.69 | 3.5 | 408 | 354 | 461 |
| **Former Yugoslavia** | 9,704 | int-high | 3.98 | 1.32 | 6.64 | 386 | 128 | 644 |
| **South Korea** | 8,558 | int-high | 4.33 | 3.94 | 4.73 | 371 | 337 | 405 |
| **India** | 8,960 | int-high | 3.23 | 2.92 | 3.55 | 289 | 262 | 318 |
| **Ghana** | 1,768 | int-high | 13.44 | 10.5 | 16.38 | 238 | 186 | 290 |
| **Syria** | 4,031 | int-high | 5.62 | 4.82 | 6.42 | 227 | 194 | 259 |
| **Bulgaria** | 5,233 | int-high | 4.25 | 2.8 | 5.7 | 222 | 147 | 298 |
| **Sri Lanka** | 7,703 | int-high | 2.41 | 0 | 5.53 | 186 | 0 | 426 |
| **Lithuania** | 8,311 | int-high | 2.03 | 1.37 | 2.69 | 169 | 114 | 224 |
| **Russia** | 5,377 | int-high | 2.89 | 2.16 | 3.62 | 155 | 116 | 195 |
| **Ukraine** | 6,408 | int-high | 2.2 | 1.15 | 3.24 | 141 | 74 | 208 |
| **Macedonia** | 2,789 | int-high | 3.29 | 2.33 | 4.24 | 92 | 65 | 118 |
| **Greece** | 2,011 | int-high | 2.33 | 1.54 | 3.11 | 47 | 31 | 63 |
| **Nepal** | 1,911 | int-high | 2.32 | 1.71 | 2.93 | 44 | 33 | 56 |
| **Poland** | 30,245 | low | 1.44 | 1.16 | 1.72 | 436 | 351 | 520 |
| **Iraq** | 21,074 | low | 1.31 | 0 | 2.87 | 276 | 0 | 605 |
| **Germany** | 34,451 | low | 0.6 | 0.4 | 0.8 | 207 | 138 | 276 |
| **Lebanon** | 12,183 | low | 1.66 | 0.86 | 2.46 | 202 | 105 | 300 |
| **Italy** | 5,940 | low | 1.89 | 1.26 | 2.52 | 112 | 75 | 150 |
| **Norway** | 18,957 | low | 0.55 | 0.34 | 0.71 | 104 | 64 | 135 |
| **Morocco** | 5,253 | low | 1.8 | 1.51 | 5.89 | 95 | 79 | 309 |
| **United Kingdom** | 17,307 | low | 0.54 | 0.3 | 0.6 | 93 | 52 | 104 |
| **Greenland (DK)** | 15,332 | low | 0.55 | 0.34 | 0.71 | 84 | 52 | 109 |
| **Faeroe Islands (DK)** | 11,494 | low | 0.55 | 0.34 | 0.71 | 63 | 39 | 82 |
| **Latvia** | 4,144 | low | 1.39 | 1.1 | 1.67 | 58 | 46 | 69 |
| **Brazil** | 3,109 | low | 1.78 | 1.54 | 2.02 | 55 | 48 | 63 |
| **France** | 6,638 | low | 0.68 | 0.44 | 1.05 | 45 | 29 | 70 |
| **Iceland** | 7,863 | low | 0.55 | 0.34 | 0.71 | 43 | 27 | 56 |
| **Sweden** | 21,087 | low | 0.2 | 0.1 | 0.4 | 42 | 21 | 84 |
| **Hungary** | 3,703 | low | 1.08 | 0.04 | 2.11 | 40 | 1 | 78 |
| **Colombia** | 3,050 | low | 1.2 | 0.27 | 2.13 | 37 | 8 | 65 |
| **Spain** | 5,497 | low | 0.66 | 0.34 | 0.97 | 36 | 19 | 53 |
| **United States** | 11,536 | low | 0.27 | 0.2 | 0.34 | 31 | 23 | 39 |
| **Canada** | 3,547 | low | 0.7 | 0.6 | 0.9 | 25 | 21 | 32 |
| **Australia** | 2,815 | low | 0.87 | 0.39 | 1.35 | 24 | 11 | 38 |
| **Switzerland** | 2,508 | low | 0.55 | 0.34 | 0.71 | 14 | 9 | 18 |
| **Japan** | 1,792 | low | 0.63 | 0.6 | 0.7 | 11 | 11 | 13 |
| **Finland** | 3,565 | low | 0.2 | 0.1 | 0.4 | 7 | 4 | 14 |
| **Netherlands** | 6,665 | low | 0.1 | 0 | 0.2 | 7 | 0 | 13 |

## Germany

| **Country** | **Population** | **HBsAg endemicity** | **HBsAg Prevalence** | | | **Estimated number of CHB cases** | | |
| --- | --- | --- | --- | --- | --- | --- | --- | --- |
|  |  |  | **%** | **LL 95%CI** | **UL 95%CI** | **Cases** | **Lower** | **Upper** |
| **Turkey** | 1,318,420 | int-high | 4.29 | 3.7 | 4.88 | 56,560 | 48,782 | 64,339 |
| **Kazakhstan** | 800,500 | int-high | 4.95 | 3.34 | 6.56 | 39,625 | 26,737 | 52,513 |
| **Russian Federation** | 975,500 | int-high | 2.89 | 2.16 | 3.62 | 28,192 | 21,071 | 35,313 |
| **Romania** | 449,920 | int-high | 5.49 | 5.24 | 5.73 | 24,701 | 23,576 | 25,780 |
| **Vietnam** | 85,430 | int-high | 12.48 | 11.46 | 13.5 | 10,662 | 9,790 | 11,533 |
| **China** | 77,620 | int-high | 10.23 | 9.35 | 11.11 | 7,941 | 7,257 | 8,624 |
| **Afghanistan** | 75,320 | int-high | 10.46 | 5.85 | 15.07 | 7,878 | 4,406 | 11,351 |
| **Yugoslavia** | 175,260 | int-high | 3.98 | 1.32 | 6.64 | 6,975 | 2,313 | 11,637 |
| **Bosnia and Herzegovina** | 159,380 | int-high | 3.63 | 2.26 | 5 | 5,785 | 3,602 | 7,969 |
| **Ukraine** | 205,970 | int-high | 2.2 | 1.15 | 3.24 | 4,531 | 2,369 | 6,673 |
| **Kosovo** | 134,940 | int-high | 3.29 | 2.33 | 4.24 | 4,440 | 3,144 | 5,721 |
| **Greece** | 160,390 | int-high | 2.33 | 1.54 | 3.11 | 3,737 | 2,470 | 4,988 |
| **Thailand** | 58,210 | int-high | 5.54 | 4.64 | 6.43 | 3,225 | 2,701 | 3,743 |
| **Ghana** | 23,680 | int-high | 13.44 | 10.5 | 16.38 | 3,183 | 2,486 | 3,879 |
| **Iran** | 101,660 | int-high | 3.1 | 2.69 | 3.5 | 3,151 | 2,735 | 3,558 |
| **Bulgaria** | 71,250 | int-high | 4.25 | 2.8 | 5.7 | 3,028 | 1,995 | 4,061 |
| **Philippines** | 39,240 | int-high | 7.36 | 6.32 | 8.39 | 2,888 | 2,480 | 3,292 |
| **Kyrgyzstan** | 68,310 | int-high | 3.61 | 3.05 | 4.19 | 2,466 | 2,083 | 2,862 |
| **Syria** | 39,200 | int-high | 5.62 | 4.82 | 6.42 | 2,203 | 1,889 | 2,517 |
| **Uzbekistan** | 33,110 | int-high | 6.34 | 4.22 | 8.46 | 2,099 | 1,397 | 2,801 |
| **FYR Macedonia** | 54,370 | int-high | 3.29 | 2.33 | 4.24 | 1,789 | 1,267 | 2,305 |
| **Pakistan** | 40,200 | int-high | 4.17 | 3.59 | 4.75 | 1,676 | 1,443 | 1,910 |
| **India** | 50,790 | int-high | 3.23 | 2.92 | 3.55 | 1,641 | 1,483 | 1,803 |
| **Tunisia** | 33,160 | int-high | 4.9 | 1.51 | 5.89 | 1,625 | 501 | 1,953 |
| **Serbia** | 40,750 | int-high | 3.29 | 2.33 | 4.24 | 1,341 | 949 | 1,728 |
| **Slovenia** | 29,990 | int-high | 3.29 | 2.33 | 4.24 | 987 | 699 | 1,272 |
| **Tajikistan** | 24,030 | int-high | 3.61 | 3.05 | 4.19 | 867 | 733 | 1,007 |
| **Sri Lanka** | 35,000 | int-high | 2.41 | 0 | 5.53 | 844 | 0 | 1,936 |
| **Lithuania** | 37,100 | int-high | 2.03 | 1.37 | 2.69 | 753 | 508 | 998 |
| **Poland** | 2,749,670 | low | 1.44 | 1.16 | 1.72 | 39,595 | 31,896 | 47,294 |
| **Italy** | 330,730 | low | 1.89 | 1.26 | 2.52 | 6,251 | 4,167 | 8,334 |
| **Czech Republic** | 441,640 | low | 0.7 | 0.43 | 0.98 | 3,091 | 1,899 | 4,328 |
| **Croatia** | 162,800 | low | 1.47 | 0.84 | 2.1 | 2,393 | 1,368 | 3,419 |
| **Morocco** | 91,270 | low | 1.8 | 1.51 | 5.89 | 1,643 | 1,378 | 5,376 |
| **Hungary** | 125,520 | low | 1.08 | 0.04 | 2.11 | 1,356 | 50 | 2,648 |
| **Austria** | 205,050 | low | 0.55 | 0.34 | 0.71 | 1,128 | 697 | 1,456 |
| **Iraq** | 85,130 | low | 1.31 | 0 | 2.87 | 1,115 | 0 | 2,443 |
| **Lebanon** | 61,960 | low | 1.66 | 0.86 | 2.46 | 1,029 | 533 | 1,524 |
| **Portugal** | 75,110 | low | 1.35 | 0.66 | 2.04 | 1,014 | 496 | 1,532 |
| **Brazil** | 47,590 | low | 1.78 | 1.54 | 2.02 | 847 | 733 | 961 |
| **France** | 109,260 | low | 0.68 | 0.44 | 1.05 | 743 | 481 | 1,147 |
| **Spain** | 75,670 | low | 0.66 | 0.34 | 0.97 | 499 | 257 | 734 |
| **United Kingdom** | 80,290 | low | 0.54 | 0.3 | 0.6 | 434 | 241 | 482 |
| **Switzerland** | 64,930 | low | 0.55 | 0.34 | 0.71 | 357 | 221 | 461 |
| **United States** | 97,580 | low | 0.27 | 0.2 | 0.34 | 263 | 195 | 332 |
| **Slovakia** | 32,640 | low | 0.7 | 0.43 | 0.98 | 228 | 140 | 320 |
| **Belgium** | 26,240 | low | 0.7 | 0.4 | 1.2 | 184 | 105 | 315 |
| **Japan** | 28,320 | low | 0.63 | 0.6 | 0.7 | 178 | 170 | 198 |
| **Czechoslovakia** | 24,400 | low | 0.7 | 0.43 | 0.98 | 171 | 105 | 239 |
| **Netherlands** | 112,360 | low | 0.1 | 0 | 0.2 | 112 | 0 | 225 |

## Finland

| **Country** | **Population** | **HBsAg endemicity** | **HBsAg Prevalence** | | | **Estimated number of CHB cases** | | |
| --- | --- | --- | --- | --- | --- | --- | --- | --- |
|  |  |  | **%** | **LL 95%CI** | **UL 95%CI** | **Cases** | **Lower** | **Upper** |
| **Former Soviet Union** | 52,339 | int-high | 3,83 | 2,74 | 4,91 | 2,005 | 1,434 | 2,570 |
| **Somalia** | 9,079 | int-high | 12.4 | 8.89 | 15.92 | 1,126 | 807 | 1,445 |
| **China** | 8,272 | int-high | 10.23 | 9.35 | 11.11 | 846 | 773 | 919 |
| **Vietnam** | 5,176 | int-high | 12.48 | 11.46 | 13.5 | 646 | 593 | 699 |
| **Thailand** | 8,050 | int-high | 5.54 | 4.64 | 6.43 | 446 | 374 | 518 |
| **Afghanistan** | 3,288 | int-high | 10.46 | 5.85 | 15.07 | 344 | 192 | 496 |
| **Russia** | 10,020 | int-high | 2.89 | 2.16 | 3.62 | 290 | 216 | 363 |
| **Former Yugoslavia** | 6,515 | int-high | 3.98 | 1.32 | 6.64 | 259 | 86 | 433 |
| **Turkey** | 5,736 | int-high | 4.29 | 3.7 | 4.88 | 246 | 212 | 280 |
| **Nigeria** | 1,759 | int-high | 13.31 | 11.57 | 15.06 | 234 | 204 | 265 |
| **Philippines** | 2,385 | int-high | 7.36 | 6.32 | 8.39 | 176 | 151 | 200 |
| **Sudan** | 829 | int-high | 18.59 | 14.22 | 22.96 | 154 | 118 | 190 |
| **Iran** | 4,864 | int-high | 3.1 | 2.69 | 3.5 | 151 | 131 | 170 |
| **Ghana** | 1,119 | int-high | 13.44 | 10.5 | 16.38 | 150 | 117 | 183 |
| **DRC Congo** | 1,312 | int-high | 11.44 | 8.46 | 14.43 | 150 | 111 | 189 |
| **India** | 4,556 | int-high | 3.23 | 2.92 | 3.55 | 147 | 133 | 162 |
| **Myanmar (Burma)** | 1,089 | int-high | 11.63 | 9.53 | 13.73 | 127 | 104 | 150 |
| **Romania** | 2,117 | int-high | 5.49 | 5.24 | 5.73 | 116 | 111 | 121 |
| **Ethiopia** | 1,729 | int-high | 5.47 | 2.55 | 8.39 | 95 | 44 | 145 |
| **Bangladesh** | 1,658 | int-high | 4.83 | 4.02 | 5.64 | 80 | 67 | 94 |
| **Pakistan** | 1,775 | int-high | 4.17 | 3.59 | 4.75 | 74 | 64 | 84 |
| **Bulgaria** | 1,535 | int-high | 4.25 | 2.8 | 5.7 | 65 | 43 | 87 |
| **Kenya** | 1,084 | int-high | 5.7 | 4.21 | 7.2 | 62 | 46 | 78 |
| **Nepal** | 1,757 | int-high | 2.32 | 1.71 | 2.93 | 41 | 30 | 51 |
| **Egypt** | 931 | int-high | 4.18 | 1.85 | 6.51 | 39 | 17 | 61 |
| **Ukraine** | 1,236 | int-high | 2.2 | 1.15 | 3.24 | 27 | 14 | 40 |
| **Greece** | 902 | int-high | 2.33 | 1.54 | 3.11 | 21 | 14 | 28 |
| **Sri Lanka** | 841 | int-high | 2.41 | 0 | 5.53 | 20 | 0 | 47 |
| **Estonia** | 34,984 | low | 0.58 | 0.42 | 0.74 | 203 | 147 | 259 |
| **Iraq** | 8,404 | low | 1.31 | 0 | 2.87 | 110 | 0 | 241 |
| **Sweden** | 31,601 | low | 0.2 | 0.1 | 0.4 | 63 | 32 | 126 |
| **Poland** | 3,399 | low | 1.44 | 1.16 | 1.72 | 49 | 39 | 58 |
| **Italy** | 2,068 | low | 1.89 | 1.26 | 2.52 | 39 | 26 | 52 |
| **Germany** | 6,219 | low | 0.6 | 0.4 | 0.8 | 37 | 25 | 50 |
| **Morocco** | 1,814 | low | 1.8 | 1.51 | 5.89 | 33 | 27 | 107 |
| **United Kingdom** | 5,115 | low | 0.54 | 0.3 | 0.6 | 28 | 15 | 31 |
| **Hungary** | 1,889 | low | 1.08 | 0.04 | 2.11 | 20 | 1 | 40 |
| **Brazil** | 1,119 | low | 1.78 | 1.54 | 2.02 | 20 | 17 | 23 |
| **Latvia** | 1,361 | low | 1.39 | 1.1 | 1.67 | 19 | 15 | 23 |
| **France** | 2,135 | low | 0.68 | 0.44 | 1.05 | 15 | 9 | 22 |
| **Spain** | 2,012 | low | 0.66 | 0.34 | 0.97 | 13 | 7 | 20 |
| **United States** | 4,383 | low | 0.27 | 0.2 | 0.34 | 12 | 9 | 15 |
| **Canada** | 1,515 | low | 0.7 | 0.6 | 0.9 | 11 | 9 | 14 |
| **Norway** | 1,587 | low | 0.55 | 0.34 | 0.71 | 9 | 5 | 11 |
| **Australia** | 993 | low | 0.87 | 0.39 | 1.35 | 9 | 4 | 13 |
| **Japan** | 1,237 | low | 0.63 | 0.6 | 0.7 | 8 | 7 | 9 |
| **Switzerland** | 987 | low | 0.55 | 0.34 | 0.71 | 5 | 3 | 7 |
| **Denmark** | 916 | low | 0.55 | 0.34 | 0.71 | 5 | 3 | 7 |

## Estonia

| **Country** | **Population** | **HBsAg endemicity** | **HBsAg Prevalence** | | | **Estimated number of CHB cases** | | |
| --- | --- | --- | --- | --- | --- | --- | --- | --- |
|  |  |  | **%** | **LL 95%CI** | **UL 95%CI** | **Cases** | **Lower** | **Upper** |
| **Russia** | 138,501 | int-high | 2.89 | 2.16 | 3.62 | 4,003 | 2,992 | 5,014 |
| **Ukraine** | 21,743 | int-high | 2.2 | 1.15 | 3.24 | 478 | 250 | 704 |
| **Belarus** | 11,616 | int-high | 3.19 | 1.72 | 4.65 | 371 | 200 | 540 |
| **Kazakhstan** | 3,802 | int-high | 4.95 | 3.34 | 6.56 | 188 | 127 | 249 |
| **Moldova** | 842 | int-high | 9.61 | 6.92 | 12.29 | 81 | 58 | 103 |
| **Uzbekistan** | 1,090 | int-high | 6.34 | 4.22 | 8.46 | 69 | 46 | 92 |
| **Georgia** | 1,528 | int-high | 3.89 | 1.25 | 6.54 | 59 | 19 | 100 |
| **Azerbaijan** | 1,501 | int-high | 3.11 | 1.39 | 4.84 | 47 | 21 | 73 |
| **Lithuania** | 1,886 | int-high | 2.03 | 1.37 | 2.69 | 38 | 26 | 51 |
| **China** | 228 | int-high | 10.23 | 9.35 | 11.11 | 23 | 21 | 25 |
| **Kyrgyzstan** | 550 | int-high | 3.61 | 3.05 | 4.19 | 20 | 17 | 23 |
| **Tajikistan** | 369 | int-high | 3.61 | 3.05 | 4.19 | 13 | 11 | 15 |
| **Turkmenistan** | 340 | int-high | 3.61 | 3.05 | 4.19 | 12 | 10 | 14 |
| **India** | 169 | int-high | 3.23 | 2.92 | 3.55 | 5 | 5 | 6 |
| **Turkey** | 120 | int-high | 4.29 | 3.7 | 4.88 | 5 | 4 | 6 |
| **Mongolia** | 50 | int-high | 8.97 | 8.47 | 9.48 | 4 | 4 | 5 |
| **Bulgaria** | 89 | int-high | 4.25 | 2.8 | 5.7 | 4 | 2 | 5 |
| **Romania** | 63 | int-high | 5.49 | 5.24 | 5.73 | 3 | 3 | 4 |
| **Egypt** | 49 | int-high | 4.18 | 1.85 | 6.51 | 2 | 1 | 3 |
| **Thailand** | 31 | int-high | 5.54 | 4.64 | 6.43 | 2 | 1 | 2 |
| **Pakistan** | 41 | int-high | 4.17 | 3.59 | 4.75 | 2 | 1 | 2 |
| **Iran** | 34 | int-high | 3.1 | 2.69 | 3.5 | 1 | 1 | 1 |
| **Latvia** | 4,129 | low | 1.39 | 1.1 | 1.67 | 57 | 45 | 69 |
| **Poland** | 563 | low | 1.44 | 1.16 | 1.72 | 8 | 7 | 10 |
| **Germany** | 1,306 | low | 0.6 | 0.4 | 0.8 | 8 | 5 | 10 |
| **Italy** | 327 | low | 1.89 | 1.26 | 2.52 | 6 | 4 | 8 |
| **Finland** | 2,444 | low | 0.2 | 0.1 | 0.4 | 5 | 2 | 10 |
| **Armenia** | 663 | low | 0.57 | 0.19 | 0.94 | 4 | 1 | 6 |
| **United Kingdom** | 592 | low | 0.54 | 0.3 | 0.6 | 3 | 2 | 4 |
| **United States** | 697 | low | 0.27 | 0.2 | 0.34 | 2 | 1 | 2 |
| **France** | 225 | low | 0.68 | 0.44 | 1.05 | 2 | 1 | 2 |
| **Spain** | 184 | low | 0.66 | 0.34 | 0.97 | 1 | 1 | 2 |
| **Hungary** | 112 | low | 1.08 | 0.04 | 2.11 | 1 | 0 | 2 |
| **Brazil** | 62 | low | 1.78 | 1.54 | 2.02 | 1 | 1 | 1 |
| **Sweden** | 489 | low | 0.2 | 0.1 | 0.4 | 1 | 0 | 2 |
| **Norway** | 166 | low | 0.55 | 0.34 | 0.71 | 1 | 1 | 1 |
| **Belgium** | 125 | low | 0.7 | 0.4 | 1.2 | 1 | 1 | 2 |
| **Canada** | 119 | low | 0.7 | 0.6 | 0.9 | 1 | 1 | 1 |
| **Denmark** | 147 | low | 0.55 | 0.34 | 0.71 | 1 | 0 | 1 |
| **Israel** | 56 | low | 1.26 | 0.97 | 1.55 | 1 | 1 | 1 |
| **Australia** | 78 | low | 0.87 | 0.39 | 1.35 | 1 | 0 | 1 |
| **Portugal** | 42 | low | 1.35 | 0.66 | 2.04 | 1 | 0 | 1 |
| **Czech Republic** | 64 | low | 0.7 | 0.43 | 0.98 | 0 | 0 | 1 |
| **Japan** | 61 | low | 0.63 | 0.6 | 0.7 | 0 | 0 | 0 |
| **Austria** | 64 | low | 0.55 | 0.34 | 0.71 | 0 | 0 | 0 |
| **Switzerland** | 51 | low | 0.55 | 0.34 | 0.71 | 0 | 0 | 0 |
| **Mexico** | 37 | low | 0.49 | 0.34 | 0.65 | 0 | 0 | 0 |
| **Ireland** | 168 | low | 0.1 | 0 | 0.3 | 0 | 0 | 1 |
| **Netherlands** | 131 | low | 0.1 | 0 | 0.2 | 0 | 0 | 0 |

## France

| **Country** | **Population** | **HBsAg endemicity** | **HBsAg Prevalence** | | | **Estimated number of CHB cases** | | |
| --- | --- | --- | --- | --- | --- | --- | --- | --- |
|  |  |  | **%** | **LL 95%CI** | **UL 95%CI** | **Cases** | **Lower** | **Upper** |
| **Algeria** | 1,370,617 | int-high | 2.6 | 0 | 12.19 | 35,636 | 0 | 167,078 |
| **Tunisia** | 376,254 | int-high | 4.9 | 1.51 | 5.89 | 18,436 | 5,681 | 22,161 |
| **Vietnam** | 119,606 | int-high | 12.48 | 11.46 | 13.5 | 14,927 | 13,707 | 16,147 |
| **Senegal** | 114,405 | int-high | 12.66 | 10.14 | 15.18 | 14,484 | 11,601 | 17,367 |
| **Ivory Coast** | 89,324 | int-high | 13.17 | 10.18 | 16.17 | 11,764 | 9,093 | 14,444 |
| **Madagascar** | 116,405 | int-high | 9.66 | 7.06 | 12.26 | 11,245 | 8,218 | 14,271 |
| **Turkey** | 256,409 | int-high | 4.29 | 3.7 | 4.88 | 11,000 | 9,487 | 12,513 |
| **China** | 96,301 | int-high | 10.23 | 9.35 | 11.11 | 9,852 | 9,004 | 10,699 |
| **Cameroon** | 79,587 | int-high | 11.44 | 8.46 | 14.43 | 9,105 | 6,733 | 11,484 |
| **Mali** | 68,882 | int-high | 13.17 | 10.18 | 16.17 | 9,072 | 7,012 | 11,138 |
| **Congo** | 67,908 | int-high | 11.44 | 8.46 | 14.43 | 7,769 | 5,745 | 9,799 |
| **DRC Congo** | 64,893 | int-high | 11.44 | 8.46 | 14.43 | 7,424 | 5,490 | 9,364 |
| **Cambodia** | 60,539 | int-high | 10.27 | 7.01 | 13.53 | 6,217 | 4,244 | 8,191 |
| **Laos** | 40,713 | int-high | 13.61 | 11.58 | 15.64 | 5,541 | 4,715 | 6,368 |
| **Guinea** | 29,619 | int-high | 16.33 | 14.61 | 18.05 | 4,837 | 4,327 | 5,346 |
| **Romania** | 71,751 | int-high | 5.49 | 5.24 | 5.73 | 3,939 | 3,760 | 4,111 |
| **Mauritius** | 42,082 | int-high | 9.32 | 8.15 | 10.48 | 3,922 | 3,430 | 4,410 |
| **Haiti** | 74,042 | int-high | 4.81 | 3.93 | 5.68 | 3,561 | 2,910 | 4,206 |
| **Comoros** | 36,508 | int-high | 9.66 | 7.06 | 12.26 | 3,527 | 2,577 | 4,476 |
| **Togo** | 22,883 | int-high | 13.17 | 10.18 | 16.17 | 3,014 | 2,329 | 3,700 |
| **Benin** | 19,568 | int-high | 13.17 | 10.18 | 16.17 | 2,577 | 1,992 | 3,164 |
| **Serbia** | 73,953 | int-high | 3.29 | 2.33 | 4.24 | 2,433 | 1,723 | 3,136 |
| **Gabon** | 19,514 | int-high | 11.44 | 8.46 | 14.43 | 2,232 | 1,651 | 2,816 |
| **India** | 47,499 | int-high | 3.23 | 2.92 | 3.55 | 1,534 | 1,387 | 1,686 |
| **Russian Federation** | 52,462 | int-high | 2.89 | 2.16 | 3.62 | 1,516 | 1,133 | 1,899 |
| **Cape Verde** | 22,308 | int-high | 5.65 | 0.16 | 11.14 | 1,260 | 36 | 2,485 |
| **Egypt** | 29,990 | int-high | 4.18 | 1.85 | 6.51 | 1,254 | 555 | 1,952 |
| **Suriname** | 24,361 | int-high | 4.52 | 2.47 | 6.57 | 1,101 | 602 | 1,601 |
| **Sri Lanka** | 40,633 | int-high | 2.41 | 0 | 5.53 | 979 | 0 | 2,247 |
| **South Korea** | 19,959 | int-high | 4.33 | 3.94 | 4.73 | 864 | 786 | 944 |
| **Pakistan** | 19,893 | int-high | 4.17 | 3.59 | 4.75 | 830 | 714 | 945 |
| **Iran** | 22,134 | int-high | 3.1 | 2.69 | 3.5 | 686 | 595 | 775 |
| **Morocco** | 892,035 | low | 1.8 | 1.51 | 5.89 | 16,057 | 13,470 | 52,541 |
| **Portugal** | 617,235 | low | 1.35 | 0.66 | 2.04 | 8,333 | 4,074 | 12,592 |
| **Italy** | 345,038 | low | 1.89 | 1.26 | 2.52 | 6,521 | 4,347 | 8,695 |
| **Spain** | 288,168 | low | 0.66 | 0.34 | 0.97 | 1,902 | 980 | 2,795 |
| **Poland** | 102,740 | low | 1.44 | 1.16 | 1.72 | 1,479 | 1,192 | 1,767 |
| **Germany** | 219,966 | low | 0.6 | 0.4 | 0.8 | 1,320 | 880 | 1,760 |
| **Belgium** | 147,650 | low | 0.7 | 0.4 | 1.2 | 1,034 | 591 | 1,772 |
| **Brazil** | 56,712 | low | 1.78 | 1.54 | 2.02 | 1,009 | 873 | 1,146 |
| **United Kingdom** | 169,945 | low | 0.54 | 0.3 | 0.6 | 918 | 510 | 1,020 |
| **Lebanon** | 44,578 | low | 1.66 | 0.86 | 2.46 | 740 | 383 | 1,097 |
| **Switzerland** | 92,605 | low | 0.55 | 0.34 | 0.71 | 509 | 315 | 657 |
| **Colombia** | 26,679 | low | 1.2 | 0.27 | 2.13 | 320 | 72 | 568 |
| **Canada** | 25,203 | low | 0.7 | 0.6 | 0.9 | 176 | 151 | 227 |
| **United States of America** | 52,053 | low | 0.27 | 0.2 | 0.34 | 141 | 104 | 177 |
| **Monaco** | 23,745 | low | 0.55 | 0.34 | 0.71 | 131 | 81 | 169 |
| **Japan** | 19,708 | low | 0.63 | 0.6 | 0.7 | 124 | 118 | 138 |
| **Armenia** | 20,270 | low | 0.57 | 0.19 | 0.94 | 116 | 39 | 191 |
| **Netherlands** | 40,616 | low | 0.1 | 0 | 0.2 | 41 | 0 | 81 |

## Greece

| **Country** | **Population** | **HBsAg endemicity** | **HBsAg Prevalence** | | | **Estimated number of CHB cases** | | |
| --- | --- | --- | --- | --- | --- | --- | --- | --- |
|  |  |  | **%** | **LL 95%CI** | **UL 95%CI** | **Cases** | **Lower** | **Upper** |
| **Albania** | 357,103 | int-high | 9 | 8.1 | 9.8 | 32,139 | 28,925 | 34,996 |
| **Georgia** | 54,192 | int-high | 3.89 | 1.25 | 6.54 | 2,108 | 677 | 3,544 |
| **Romania** | 32,717 | int-high | 5.49 | 5.24 | 5.73 | 1,796 | 1,714 | 1,875 |
| **Bulgaria** | 35,037 | int-high | 4.25 | 2.8 | 5.7 | 1,489 | 981 | 1,997 |
| **Russia** | 37,762 | int-high | 2.89 | 2.16 | 3.62 | 1,091 | 816 | 1,367 |
| **Pakistan** | 24,038 | int-high | 4.17 | 3.59 | 4.75 | 1,002 | 863 | 1,142 |
| **Syria** | 10,036 | int-high | 5.62 | 4.82 | 6.42 | 564 | 484 | 644 |
| **Philippines** | 6,603 | int-high | 7.36 | 6.32 | 8.39 | 486 | 417 | 554 |
| **Egypt** | 11,378 | int-high | 4.18 | 1.85 | 6.51 | 476 | 210 | 741 |
| **Turkey** | 9,388 | int-high | 4.29 | 3.7 | 4.88 | 403 | 347 | 458 |
| **Bangladesh** | 7,525 | int-high | 4.83 | 4.02 | 5.64 | 363 | 303 | 424 |
| **Ukraine** | 11,476 | int-high | 2.2 | 1.15 | 3.24 | 252 | 132 | 372 |
| **Moldova** | 1,784 | int-high | 9.61 | 6.92 | 12.29 | 171 | 123 | 219 |
| **China** | 1,582 | int-high | 10.23 | 9.35 | 11.11 | 162 | 148 | 176 |
| **India** | 4,062 | int-high | 3.23 | 2.92 | 3.55 | 131 | 119 | 144 |
| **South Africa** | 1,985 | int-high | 6.2 | 4.68 | 7.71 | 123 | 93 | 153 |
| **Sudan** | 604 | int-high | 18.59 | 14.22 | 22.96 | 112 | 86 | 139 |
| **Serbia** | 2,394 | int-high | 3.29 | 2.33 | 4.24 | 79 | 56 | 102 |
| **Indonesia** | 1,515 | int-high | 3.93 | 3.08 | 4.77 | 60 | 47 | 72 |
| **Thailand** | 630 | int-high | 5.54 | 4.64 | 6.43 | 35 | 29 | 41 |
| **Iran** | 809 | int-high | 3.1 | 2.69 | 3.5 | 25 | 22 | 28 |
| **Macedonia** | 709 | int-high | 3.29 | 2.33 | 4.24 | 23 | 17 | 30 |
| **Tunisia** | 436 | int-high | 4.9 | 1.51 | 5.89 | 21 | 7 | 26 |
| **Sri Lanka** | 782 | int-high | 2.41 | 0 | 5.53 | 19 | 0 | 43 |
| **Lithuania** | 807 | int-high | 2.03 | 1.37 | 2.69 | 16 | 11 | 22 |
| **Libya** | 632 | int-high | 2.2 | 1.51 | 5.89 | 14 | 10 | 37 |
| **Poland** | 9,392 | low | 1.44 | 1.16 | 1.72 | 135 | 109 | 162 |
| **Germany** | 21,194 | low | 0.6 | 0.4 | 0.8 | 127 | 85 | 170 |
| **Cyprus** | 10,256 | low | 0.9 | 0.3 | 2 | 92 | 31 | 205 |
| **Italy** | 3,425 | low | 1.89 | 1.26 | 2.52 | 65 | 43 | 86 |
| **Morocco** | 3,308 | low | 1.8 | 1.51 | 5.89 | 60 | 50 | 195 |
| **Armenia** | 9,563 | low | 0.57 | 0.19 | 0.94 | 55 | 18 | 90 |
| **United Kingdom** | 8,766 | low | 0.54 | 0.3 | 0.6 | 47 | 26 | 53 |
| **Canada** | 5,305 | low | 0.7 | 0.6 | 0.9 | 37 | 32 | 48 |
| **Australia** | 2,895 | low | 0.87 | 0.39 | 1.35 | 25 | 11 | 39 |
| **France** | 3,630 | low | 0.68 | 0.44 | 1.05 | 25 | 16 | 38 |
| **Venezuela** | 1,159 | low | 1.93 | 1.13 | 2.73 | 22 | 13 | 32 |
| **United States** | 7,379 | low | 0.27 | 0.2 | 0.34 | 20 | 15 | 25 |
| **Iraq** | 1,147 | low | 1.31 | 0 | 2.87 | 15 | 0 | 33 |
| **Czech Republic** | 1,821 | low | 0.7 | 0.43 | 0.98 | 13 | 8 | 18 |
| **Lebanon** | 537 | low | 1.66 | 0.86 | 2.46 | 9 | 5 | 13 |
| **Belgium** | 1,261 | low | 0.7 | 0.4 | 1.2 | 9 | 5 | 15 |
| **Brazil** | 495 | low | 1.78 | 1.54 | 2.02 | 9 | 8 | 10 |
| **Hungary** | 633 | low | 1.08 | 0.04 | 2.11 | 7 | 0 | 13 |
| **Switzerland** | 1,041 | low | 0.55 | 0.34 | 0.71 | 6 | 4 | 7 |
| **Israel** | 397 | low | 1.26 | 0.97 | 1.55 | 5 | 4 | 6 |
| **Austria** | 748 | low | 0.55 | 0.34 | 0.71 | 4 | 3 | 5 |
| **Finland** | 1,885 | low | 0.2 | 0.1 | 0.4 | 4 | 2 | 8 |
| **Sweden** | 691 | low | 0.2 | 0.1 | 0.4 | 1 | 1 | 3 |
| **Ireland** | 557 | low | 0.1 | 0 | 0.3 | 1 | 0 | 2 |

## Hungary

| **Country** | **Population** | **HBsAg endemicity** | **HBsAg Prevalence** | | | **Estimated number of CHB cases** | | |
| --- | --- | --- | --- | --- | --- | --- | --- | --- |
|  |  |  | **%** | **LL 95%CI** | **UL 95%CI** | **Cases** | **Lower** | **Upper** |
| **Romania** | 190,942 | int-high | 5.49 | 5.24 | 5.73 | 10,483 | 10,005 | 10,941 |
| **Serbia** | 35,944 | int-high | 3.29 | 2.33 | 4.24 | 1,183 | 837 | 1,524 |
| **China** | 9,890 | int-high | 10.23 | 9.35 | 11.11 | 1,012 | 925 | 1,099 |
| **Ukraine** | 28,779 | int-high | 2.2 | 1.15 | 3.24 | 633 | 331 | 932 |
| **Former Soviet Union** | 14,070 | int-high | 3.83 | 2.74 | 4.91 | 539 | 386 | 691 |
| **Vietnam** | 3,166 | int-high | 12.48 | 11.46 | 13.5 | 395 | 363 | 427 |
| **Nigeria** | 1,302 | int-high | 13.31 | 11.57 | 15.06 | 173 | 151 | 196 |
| **Afghanistan** | 1,152 | int-high | 10.46 | 5.85 | 15.07 | 120 | 67 | 174 |
| **Russia** | 3,155 | int-high | 2.89 | 2.16 | 3.62 | 91 | 68 | 114 |
| **Mongolia** | 1,010 | int-high | 8.97 | 8.47 | 9.48 | 91 | 86 | 96 |
| **Turkey** | 2,023 | int-high | 4.29 | 3.7 | 4.88 | 87 | 75 | 99 |
| **Syria** | 1,165 | int-high | 5.62 | 4.82 | 6.42 | 65 | 56 | 75 |
| **Iran** | 2,083 | int-high | 3.1 | 2.69 | 3.5 | 65 | 56 | 73 |
| **Somalia** | 476 | int-high | 12.4 | 8.89 | 15.92 | 59 | 42 | 76 |
| **Bulgaria** | 1,338 | int-high | 4.25 | 2.8 | 5.7 | 57 | 37 | 76 |
| **South Korea** | 1,088 | int-high | 4.33 | 3.94 | 4.73 | 47 | 43 | 51 |
| **Thailand** | 724 | int-high | 5.54 | 4.64 | 6.43 | 40 | 34 | 47 |
| **Egypt** | 794 | int-high | 4.18 | 1.85 | 6.51 | 33 | 15 | 52 |
| **India** | 1,011 | int-high | 3.23 | 2.92 | 3.55 | 33 | 30 | 36 |
| **Greece** | 1,149 | int-high | 2.33 | 1.54 | 3.11 | 27 | 18 | 36 |
| **Kazakhstan** | 477 | int-high | 4.95 | 3.34 | 6.56 | 24 | 16 | 31 |
| **Algeria** | 637 | int-high | 2.6 | 0 | 12.19 | 17 | 0 | 78 |
| **Slovenia** | 406 | int-high | 3.29 | 2.33 | 4.24 | 13 | 9 | 17 |
| **Germany** | 27,320 | low | 0.6 | 0.4 | 0.8 | 164 | 109 | 219 |
| **Slovakia** | 21,258 | low | 0.7 | 0.43 | 0.98 | 149 | 91 | 208 |
| **Italy** | 3,872 | low | 1.89 | 1.26 | 2.52 | 73 | 49 | 98 |
| **Austria** | 8,079 | low | 0.55 | 0.34 | 0.71 | 44 | 27 | 57 |
| **Poland** | 2,851 | low | 1.44 | 1.16 | 1.72 | 41 | 33 | 49 |
| **Former Czechoslovakia** | 5,803 | low | 0.7 | 0.43 | 0.98 | 41 | 25 | 57 |
| **United Kingdom** | 5,595 | low | 0.54 | 0.3 | 0.6 | 30 | 17 | 34 |
| **Croatia** | 2,017 | low | 1.47 | 0.84 | 2.1 | 30 | 17 | 42 |
| **France** | 3,665 | low | 0.68 | 0.44 | 1.05 | 25 | 16 | 38 |
| **Israel** | 1,646 | low | 1.26 | 0.97 | 1.55 | 21 | 16 | 26 |
| **United States** | 7,249 | low | 0.27 | 0.2 | 0.34 | 20 | 14 | 25 |
| **Canada** | 2,094 | low | 0.7 | 0.6 | 0.9 | 15 | 13 | 19 |
| **Switzerland** | 2,166 | low | 0.55 | 0.34 | 0.71 | 12 | 7 | 15 |
| **Belgium** | 1,641 | low | 0.7 | 0.4 | 1.2 | 11 | 7 | 20 |
| **Czech Republic** | 1,549 | low | 0.7 | 0.43 | 0.98 | 11 | 7 | 15 |
| **Iraq** | 813 | low | 1.31 | 0 | 2.87 | 11 | 0 | 23 |
| **Brazil** | 561 | low | 1.78 | 1.54 | 2.02 | 10 | 9 | 11 |
| **Spain** | 1,342 | low | 0.66 | 0.34 | 0.97 | 9 | 5 | 13 |
| **Australia** | 912 | low | 0.87 | 0.39 | 1.35 | 8 | 4 | 12 |
| **Japan** | 1,241 | low | 0.63 | 0.6 | 0.7 | 8 | 7 | 9 |
| **Norway** | 1,074 | low | 0.55 | 0.34 | 0.71 | 6 | 4 | 8 |
| **Cuba** | 528 | low | 0.86 | 0.58 | 1.14 | 5 | 3 | 6 |
| **Sweden** | 1,477 | low | 0.2 | 0.1 | 0.4 | 3 | 1 | 6 |
| **Netherlands** | 2,745 | low | 0.1 | 0 | 0.2 | 3 | 0 | 5 |
| **Mexico** | 443 | low | 0.49 | 0.34 | 0.65 | 2 | 2 | 3 |
| **Finland** | 681 | low | 0.2 | 0.1 | 0.4 | 1 | 1 | 3 |

## Republic of Ireland

| **Country** | **Population** | **HBsAg endemicity** | **HBsAg Prevalence** | | | **Estimated number of CHB cases** | | |
| --- | --- | --- | --- | --- | --- | --- | --- | --- |
|  |  |  | **%** | **LL 95%CI** | **UL 95%CI** | **CHB Cases** | **Lower** | **Upper** |
| **Nigeria** | 24,938 | int-high | 13.31 | 11.57 | 15.06 | 3,319 | 2,885 | 3,756 |
| **China** | 13,798 | int-high | 10.23 | 9.35 | 11.11 | 1,412 | 1,290 | 1,533 |
| **Philippines** | 17,800 | int-high | 7.36 | 6.32 | 8.39 | 1,310 | 1,125 | 1,493 |
| **Romania** | 16,788 | int-high | 5.49 | 5.24 | 5.73 | 922 | 880 | 962 |
| **India** | 22,222 | int-high | 3.23 | 2.92 | 3.55 | 718 | 649 | 789 |
| **Lithuania** | 32,639 | int-high | 2.03 | 1.37 | 2.69 | 663 | 447 | 878 |
| **South Africa** | 10,260 | int-high | 6.2 | 4.68 | 7.71 | 636 | 480 | 791 |
| **Zimbabwe** | 3,510 | int-high | 13.91 | 10.7 | 17.11 | 488 | 376 | 601 |
| **Pakistan** | 10,192 | int-high | 4.17 | 3.59 | 4.75 | 425 | 366 | 484 |
| **Moldova** | 4,240 | int-high | 9.61 | 6.92 | 12.29 | 407 | 293 | 521 |
| **Sudan** | 1,965 | int-high | 18.59 | 14.22 | 22.96 | 365 | 279 | 451 |
| **Mauritius** | 3,502 | int-high | 9.32 | 8.15 | 10.48 | 326 | 285 | 367 |
| **Congo** | 2,833 | int-high | 11.44 | 8.46 | 14.43 | 324 | 240 | 409 |
| **Malaysia** | 4,665 | int-high | 5.58 | 4.27 | 6.88 | 260 | 199 | 321 |
| **Ghana** | 1,699 | int-high | 13.44 | 10.5 | 16.38 | 228 | 178 | 278 |
| **Somalia** | 1,806 | int-high | 12.4 | 8.89 | 15.92 | 224 | 161 | 288 |
| **Russia** | 7,536 | int-high | 2.89 | 2.16 | 3.62 | 218 | 163 | 273 |
| **Bangladesh** | 3,534 | int-high | 4.83 | 4.02 | 5.64 | 171 | 142 | 199 |
| **Thailand** | 2,371 | int-high | 5.54 | 4.64 | 6.43 | 131 | 110 | 152 |
| **Saudi Arabia** | 2,438 | int-high | 5.34 | 3.78 | 6.91 | 130 | 92 | 168 |
| **Ukraine** | 5,198 | int-high | 2.2 | 1.15 | 3.24 | 114 | 60 | 168 |
| **Egypt** | 1,765 | int-high | 4.18 | 1.85 | 6.51 | 74 | 33 | 115 |
| **Bulgaria** | 1,704 | int-high | 4.25 | 2.8 | 5.7 | 72 | 48 | 97 |
| **Turkey** | 1,588 | int-high | 4.29 | 3.7 | 4.88 | 68 | 59 | 77 |
| **Bosnia and Herzegovina** | 1,503 | int-high | 3.63 | 2.26 | 5 | 55 | 34 | 75 |
| **Belarus** | 1,631 | int-high | 3.19 | 1.72 | 4.65 | 52 | 28 | 76 |
| **Algeria** | 1,696 | int-high | 2.6 | 0 | 12.19 | 44 | 0 | 207 |
| **Iran** | 1,250 | int-high | 3.1 | 2.69 | 3.5 | 39 | 34 | 44 |
| **Poland** | 107,627 | low | 1.44 | 1.16 | 1.72 | 1,550 | 1,248 | 1,851 |
| **United Kingdom** | 234,194 | low | 0.54 | 0.3 | 0.6 | 1,265 | 703 | 1,405 |
| **Latvia** | 18,836 | low | 1.39 | 1.1 | 1.67 | 262 | 207 | 315 |
| **Brazil** | 11,252 | low | 1.78 | 1.54 | 2.02 | 200 | 173 | 227 |
| **Italy** | 6,765 | low | 1.89 | 1.26 | 2.52 | 128 | 85 | 170 |
| **Hungary** | 6,956 | low | 1.08 | 0.04 | 2.11 | 75 | 3 | 147 |
| **Germany** | 12,493 | low | 0.6 | 0.4 | 0.8 | 75 | 50 | 100 |
| **Slovakia** | 9,920 | low | 0.7 | 0.43 | 0.98 | 69 | 43 | 97 |
| **Australia** | 7,524 | low | 0.87 | 0.39 | 1.35 | 65 | 29 | 102 |
| **France** | 9,476 | low | 0.68 | 0.44 | 1.05 | 64 | 42 | 99 |
| **United States** | 23,001 | low | 0.27 | 0.2 | 0.34 | 62 | 46 | 78 |
| **Spain** | 6,594 | low | 0.66 | 0.34 | 0.97 | 44 | 22 | 64 |
| **Canada** | 6,085 | low | 0.7 | 0.6 | 0.9 | 43 | 37 | 55 |
| **Czech Republic** | 5,135 | low | 0.7 | 0.43 | 0.98 | 36 | 22 | 50 |
| **Portugal** | 2,130 | low | 1.35 | 0.66 | 2.04 | 29 | 14 | 43 |
| **Iraq** | 2,151 | low | 1.31 | 0 | 2.87 | 28 | 0 | 62 |
| **Estonia** | 2,323 | low | 0.58 | 0.42 | 0.74 | 13 | 10 | 17 |
| **New Zealand** | 2,597 | low | 0.5 | 0.42 | 0.56 | 13 | 11 | 15 |
| **Switzerland** | 1,390 | low | 0.55 | 0.34 | 0.71 | 8 | 5 | 10 |
| **Netherlands** | 4,357 | low | 0.1 | 0 | 0.2 | 4 | 0 | 9 |
| **Sweden** | 1,585 | low | 0.2 | 0.1 | 0.4 | 3 | 2 | 6 |

## Iceland

| **Country** | **Population** | **HBsAg endemicity** | **HBsAg Prevalence** | | | **Estimated number of CHB cases** | | |
| --- | --- | --- | --- | --- | --- | --- | --- | --- |
|  |  |  | **%** | **LL 95%CI** | **UL 95%CI** | **Cases** | **Lower** | **Upper** |
| **Philippines** | 1,487 | int-high | 7.36 | 6.32 | 8.39 | 109 | 94 | 125 |
| **Vietnam** | 555 | int-high | 12.48 | 11.46 | 13.5 | 69 | 64 | 75 |
| **Thailand** | 1,132 | int-high | 5.54 | 4.64 | 6.43 | 63 | 53 | 73 |
| **China** | 548 | int-high | 10.23 | 9.35 | 11.11 | 56 | 51 | 61 |
| **Lithuania** | 1,408 | int-high | 2.03 | 1.37 | 2.69 | 29 | 19 | 38 |
| **Romania** | 273 | int-high | 5.49 | 5.24 | 5.73 | 15 | 14 | 16 |
| **Serbia** | 325 | int-high | 3.29 | 2.33 | 4.24 | 11 | 8 | 14 |
| **Russia** | 361 | int-high | 2.89 | 2.16 | 3.62 | 10 | 8 | 13 |
| **India** | 295 | int-high | 3.23 | 2.92 | 3.55 | 10 | 9 | 10 |
| **Bulgaria** | 151 | int-high | 4.25 | 2.8 | 5.7 | 6 | 4 | 9 |
| **Kosovo** | 171 | int-high | 3.29 | 2.33 | 4.24 | 6 | 4 | 7 |
| **Ukraine** | 255 | int-high | 2.2 | 1.15 | 3.24 | 6 | 3 | 8 |
| **Bosnia and Herzegovina** | 140 | int-high | 3.63 | 2.26 | 5 | 5 | 3 | 7 |
| **Kenya** | 87 | int-high | 5.7 | 4.21 | 7.2 | 5 | 4 | 6 |
| **Indonesia** | 120 | int-high | 3.93 | 3.08 | 4.77 | 5 | 4 | 6 |
| **Former Soviet Union** | 112 | int-high | 3.83 | 2.74 | 4.91 | 4 | 3 | 5 |
| **Sri Lanka** | 162 | int-high | 2.41 | 0 | 5.53 | 4 | 0 | 9 |
| **Yugoslavia** | 85 | int-high | 3.98 | 1.32 | 6.64 | 3 | 1 | 6 |
| **Former Serbia &Montenegro** | 93 | int-high | 3.29 | 2.33 | 4.24 | 3 | 2 | 4 |
| **Nepal** | 97 | int-high | 2.32 | 1.71 | 2.93 | 2 | 2 | 3 |
| **Poland** | 9,404 | low | 1.44 | 1.16 | 1.72 | 135 | 109 | 162 |
| **Denmark** | 3,147 | low | 0.55 | 0.34 | 0.71 | 17 | 11 | 22 |
| **Latvia** | 668 | low | 1.39 | 1.1 | 1.67 | 9 | 7 | 11 |
| **Germany** | 1,512 | low | 0.6 | 0.4 | 0.8 | 9 | 6 | 12 |
| **United Kingdom** | 1,200 | low | 0.54 | 0.3 | 0.6 | 6 | 4 | 7 |
| **Portugal** | 463 | low | 1.35 | 0.66 | 2.04 | 6 | 3 | 9 |
| **Norway** | 972 | low | 0.55 | 0.34 | 0.71 | 5 | 3 | 7 |
| **United States** | 1,967 | low | 0.27 | 0.2 | 0.34 | 5 | 4 | 7 |
| **Italy** | 223 | low | 1.89 | 1.26 | 2.52 | 4 | 3 | 6 |
| **Sweden** | 1,869 | low | 0.2 | 0.1 | 0.4 | 4 | 2 | 7 |
| **France** | 448 | low | 0.68 | 0.44 | 1.05 | 3 | 2 | 5 |
| **Morocco** | 152 | low | 1.8 | 1.51 | 5.89 | 3 | 2 | 9 |
| **Spain** | 335 | low | 0.66 | 0.34 | 0.97 | 2 | 1 | 3 |
| **Croatia** | 150 | low | 1.47 | 0.84 | 2.1 | 2 | 1 | 3 |
| **Brazil** | 122 | low | 1.78 | 1.54 | 2.02 | 2 | 2 | 2 |
| **Colombia** | 171 | low | 1.2 | 0.27 | 2.13 | 2 | 0 | 4 |
| **Canada** | 276 | low | 0.7 | 0.6 | 0.9 | 2 | 2 | 2 |
| **Hungary** | 166 | low | 1.08 | 0.04 | 2.11 | 2 | 0 | 4 |
| **Faeroe Islands (DK)** | 315 | low | 0.55 | 0.34 | 0.71 | 2 | 1 | 2 |
| **Slovakia** | 170 | low | 0.7 | 0.43 | 0.98 | 1 | 1 | 2 |
| **Czech Republic** | 152 | low | 0.7 | 0.43 | 0.98 | 1 | 1 | 1 |
| **Australia** | 110 | low | 0.87 | 0.39 | 1.35 | 1 | 0 | 1 |
| **Switzerland** | 145 | low | 0.55 | 0.34 | 0.71 | 1 | 0 | 1 |
| **Estonia** | 125 | low | 0.58 | 0.42 | 0.74 | 1 | 1 | 1 |
| **Luxembourg** | 130 | low | 0.55 | 0.34 | 0.71 | 1 | 0 | 1 |
| **Austria** | 118 | low | 0.55 | 0.34 | 0.71 | 1 | 0 | 1 |
| **Japan** | 96 | low | 0.63 | 0.6 | 0.7 | 1 | 1 | 1 |
| **Finland** | 166 | low | 0.2 | 0.1 | 0.4 | 0 | 0 | 1 |
| **Netherlands** | 281 | low | 0.1 | 0 | 0.2 | 0 | 0 | 1 |

## Italy

| **Country** | **Population** | **HBsAg endemicity** | **HBsAg Prevalence** | | | **Estimated number of CHB cases** | | |
| --- | --- | --- | --- | --- | --- | --- | --- | --- |
|  |  |  | **%** | **LL 95%CI** | **UL 95%CI** | **Cases** | **Lower** | **Upper** |
| **Romania** | 1,000,111 | int-high | 5.49 | 5.24 | 5.73 | 54,906 | 52,406 | 57,306 |
| **Albania** | 432,706 | int-high | 9 | 8.1 | 9.8 | 38,944 | 35,049 | 42,405 |
| **China** | 191,272 | int-high | 10.23 | 9.35 | 11.11 | 19,567 | 17,884 | 21,250 |
| **Moldova** | 157,145 | int-high | 9.61 | 6.92 | 12.29 | 15,102 | 10,874 | 19,313 |
| **Senegal** | 79,193 | int-high | 12.66 | 10.14 | 15.18 | 10,026 | 8,030 | 12,021 |
| **Philippines** | 135,364 | int-high | 7.36 | 6.32 | 8.39 | 9,963 | 8,555 | 11,357 |
| **Nigeria** | 52,152 | int-high | 13.31 | 11.57 | 15.06 | 6,941 | 6,034 | 7,854 |
| **Ghana** | 43,824 | int-high | 13.44 | 10.5 | 16.38 | 5,890 | 4,602 | 7,178 |
| **Tunisia** | 110,706 | int-high | 4.9 | 1.51 | 5.89 | 5,425 | 1,672 | 6,521 |
| **Ukraine** | 209,992 | int-high | 2.2 | 1.15 | 3.24 | 4,620 | 2,415 | 6,804 |
| **Egypt** | 105,481 | int-high | 4.18 | 1.85 | 6.51 | 4,409 | 1,951 | 6,867 |
| **Dominican Republic** | 39,834 | int-high | 10.68 | 5.89 | 15.46 | 4,254 | 2,346 | 6,158 |
| **Bangladesh** | 87,545 | int-high | 4.83 | 4.02 | 5.64 | 4,228 | 3,519 | 4,938 |
| **India** | 126,804 | int-high | 3.23 | 2.92 | 3.55 | 4,096 | 3,703 | 4,502 |
| **Pakistan** | 77,067 | int-high | 4.17 | 3.59 | 4.75 | 3,214 | 2,767 | 3,661 |
| **Ivory Coast** | 24,148 | int-high | 13.17 | 10.18 | 16.17 | 3,180 | 2,458 | 3,905 |
| **Bulgaria** | 57,553 | int-high | 4.25 | 2.8 | 5.7 | 2,446 | 1,611 | 3,281 |
| **FYR Macedonia** | 72,938 | int-high | 3.29 | 2.33 | 4.24 | 2,400 | 1,699 | 3,093 |
| **Russia** | 78,553 | int-high | 2.89 | 2.16 | 3.62 | 2,270 | 1,697 | 2,844 |
| **Sri Lanka** | 82,279 | int-high | 2.41 | 0 | 5.53 | 1,983 | 0 | 4,550 |
| **Ethiopia** | 30,596 | int-high | 5.47 | 2.55 | 8.39 | 1,674 | 780 | 2,567 |
| **Serbia** | 45,290 | int-high | 3.29 | 2.33 | 4.24 | 1,490 | 1,055 | 1,920 |
| **Kosovo** | 41,233 | int-high | 3.29 | 2.33 | 4.24 | 1,357 | 961 | 1,748 |
| **Belarus** | 32,589 | int-high | 3.19 | 1.72 | 4.65 | 1,040 | 561 | 1,515 |
| **Turkey** | 20,557 | int-high | 4.29 | 3.7 | 4.88 | 882 | 761 | 1,003 |
| **Libya** | 35,928 | int-high | 2.2 | 1.51 | 5.89 | 790 | 543 | 2,116 |
| **Algeria** | 23,305 | int-high | 2.6 | 0 | 12.19 | 606 | 0 | 2,841 |
| **Greece** | 20,003 | int-high | 2.33 | 1.54 | 3.11 | 466 | 308 | 622 |
| **Bolivia** | 15,143 | int-high | 3.03 | 0.08 | 5.98 | 459 | 12 | 906 |
| **Iran** | 14,098 | int-high | 3.1 | 2.69 | 3.5 | 437 | 379 | 493 |
| **Morocco** | 409,641 | low | 1.8 | 1.51 | 5.89 | 7,374 | 6,186 | 24,128 |
| **Peru** | 113,003 | low | 1.85 | 1.29 | 2.42 | 2,091 | 1,458 | 2,735 |
| **Brazil** | 108,885 | low | 1.78 | 1.54 | 2.02 | 1,938 | 1,677 | 2,199 |
| **Poland** | 122,721 | low | 1.44 | 1.16 | 1.72 | 1,767 | 1,424 | 2,111 |
| **Germany** | 219,964 | low | 0.6 | 0.4 | 0.8 | 1,320 | 880 | 1,760 |
| **Switzerland** | 191,493 | low | 0.55 | 0.34 | 0.71 | 1,053 | 651 | 1,360 |
| **France** | 136,727 | low | 0.68 | 0.44 | 1.05 | 930 | 602 | 1,436 |
| **Venezuela** | 47,847 | low | 1.93 | 1.13 | 2.73 | 923 | 541 | 1,306 |
| **Argentina** | 77,298 | low | 1 | 0.39 | 1.62 | 773 | 301 | 1,252 |
| **Colombia** | 38,656 | low | 1.2 | 0.27 | 2.13 | 464 | 104 | 823 |
| **Ecuador** | 88,391 | low | 0.47 | 0.42 | 0.51 | 415 | 371 | 451 |
| **Croatia** | 25,573 | low | 1.47 | 0.84 | 2.1 | 376 | 215 | 537 |
| **United Kingdom** | 67,548 | low | 0.54 | 0.3 | 0.6 | 365 | 203 | 405 |
| **Belgium** | 46,391 | low | 0.7 | 0.4 | 1.2 | 325 | 186 | 557 |
| **Cuba** | 31,848 | low | 0.86 | 0.58 | 1.14 | 274 | 185 | 363 |
| **Spain** | 33,404 | low | 0.66 | 0.34 | 0.97 | 220 | 114 | 324 |
| **Canada** | 25,201 | low | 0.7 | 0.6 | 0.9 | 176 | 151 | 227 |
| **Australia** | 19,825 | low | 0.87 | 0.39 | 1.35 | 172 | 77 | 268 |
| **United States** | 56,865 | low | 0.27 | 0.2 | 0.34 | 154 | 114 | 193 |
| **Austria** | 15,064 | low | 0.55 | 0.34 | 0.71 | 83 | 51 | 107 |

## Lichtenstein

| **Country** | **Population** | **HBsAg endemicity** | **HBsAg Prevalence** | | | **Estimated number of CHB cases** | | |
| --- | --- | --- | --- | --- | --- | --- | --- | --- |
|  |  |  | **%** | **LL 95%CI** | **UL 95%CI** | **Cases** | **Lower** | **Upper** |
| **Turkey** | 602 | int-high | 4.29 | 3.7 | 4.88 | 26 | 22 | 29 |
| **Bosnia and Herzegovina** | 301 | int-high | 3.63 | 2.26 | 5 | 11 | 7 | 15 |
| **Kosovo** | 236 | int-high | 3.29 | 2.33 | 4.24 | 8 | 5 | 10 |
| **China** | 73 | int-high | 10.23 | 9.35 | 11.11 | 7 | 7 | 8 |
| **Dominican Republic** | 67 | int-high | 10.68 | 5.89 | 15.46 | 7 | 4 | 10 |
| **Laos** | 35 | int-high | 13.61 | 11.58 | 15.64 | 5 | 4 | 5 |
| **Serbia** | 138 | int-high | 3.29 | 2.33 | 4.24 | 5 | 3 | 6 |
| **Thailand** | 79 | int-high | 5.54 | 4.64 | 6.43 | 4 | 4 | 5 |
| **Slovenia** | 112 | int-high | 3.29 | 2.33 | 4.24 | 4 | 3 | 5 |
| **Former Yugoslav Republic** | 112 | int-high | 3.29 | 2.33 | 4.24 | 4 | 3 | 5 |
| **Philippines** | 41 | int-high | 7.36 | 6.32 | 8.39 | 3 | 3 | 3 |
| **FYR Macedonia** | 81 | int-high | 3.29 | 2.33 | 4.24 | 3 | 2 | 3 |
| **South Africa** | 30 | int-high | 6.2 | 4.68 | 7.71 | 2 | 1 | 2 |
| **Somalia** | 15 | int-high | 12.4 | 8.89 | 15.92 | 2 | 1 | 2 |
| **Romania** | 31 | int-high | 5.49 | 5.24 | 5.73 | 2 | 2 | 2 |
| **Russia** | 49 | int-high | 2.89 | 2.16 | 3.62 | 1 | 1 | 2 |
| **Greece** | 47 | int-high | 2.33 | 1.54 | 3.11 | 1 | 1 | 1 |
| **Tunisia** | 20 | int-high | 4.9 | 1.51 | 5.89 | 1 | 0 | 1 |
| **India** | 24 | int-high | 3.23 | 2.92 | 3.55 | 1 | 1 | 1 |
| **Ukraine** | 31 | int-high | 2.2 | 1.15 | 3.24 | 1 | 0 | 1 |
| **Egypt** | 16 | int-high | 4.18 | 1.85 | 6.51 | 1 | 0 | 1 |
| **Switzerland** | 12,517 | low | 0.55 | 0.34 | 0.71 | 69 | 43 | 89 |
| **Austria** | 3,752 | low | 0.55 | 0.34 | 0.71 | 21 | 13 | 27 |
| **Italy** | 862 | low | 1.89 | 1.26 | 2.52 | 16 | 11 | 22 |
| **Germany** | 1,729 | low | 0.6 | 0.4 | 0.8 | 10 | 7 | 14 |
| **Portugal** | 404 | low | 1.35 | 0.66 | 2.04 | 5 | 3 | 8 |
| **Brazil** | 167 | low | 1.78 | 1.54 | 2.02 | 3 | 3 | 3 |
| **Croatia** | 128 | low | 1.47 | 0.84 | 2.1 | 2 | 1 | 3 |
| **Spain** | 261 | low | 0.66 | 0.34 | 0.97 | 2 | 1 | 3 |
| **France** | 119 | low | 0.68 | 0.44 | 1.05 | 1 | 1 | 1 |
| **Poland** | 54 | low | 1.44 | 1.16 | 1.72 | 1 | 1 | 1 |
| **Hungary** | 62 | low | 1.08 | 0.04 | 2.11 | 1 | 0 | 1 |
| **Colombia** | 34 | low | 1.2 | 0.27 | 2.13 | 0 | 0 | 1 |
| **Czech Republic** | 54 | low | 0.7 | 0.43 | 0.98 | 0 | 0 | 1 |
| **United States** | 129 | low | 0.27 | 0.2 | 0.34 | 0 | 0 | 0 |
| **United Kingdom** | 64 | low | 0.54 | 0.3 | 0.6 | 0 | 0 | 0 |
| **Peru** | 18 | low | 1.85 | 1.29 | 2.42 | 0 | 0 | 0 |
| **Venezuela** | 15 | low | 1.93 | 1.13 | 2.73 | 0 | 0 | 0 |
| **Belgium** | 40 | low | 0.7 | 0.4 | 1.2 | 0 | 0 | 0 |
| **Slovakia** | 36 | low | 0.7 | 0.43 | 0.98 | 0 | 0 | 0 |
| **Argentina** | 25 | low | 1 | 0.39 | 1.62 | 0 | 0 | 0 |
| **Morocco** | 13 | low | 1.8 | 1.51 | 5.89 | 0 | 0 | 1 |
| **Mexico** | 24 | low | 0.49 | 0.34 | 0.65 | 0 | 0 | 0 |
| **Canada** | 15 | low | 0.7 | 0.6 | 0.9 | 0 | 0 | 0 |
| **Japan** | 14 | low | 0.63 | 0.6 | 0.7 | 0 | 0 | 0 |
| **Denmark** | 16 | low | 0.55 | 0.34 | 0.71 | 0 | 0 | 0 |
| **Netherlands** | 74 | low | 0.1 | 0 | 0.2 | 0 | 0 | 0 |
| **Sweden** | 25 | low | 0.2 | 0.1 | 0.4 | 0 | 0 | 0 |
| **Ireland** | 15 | low | 0.1 | 0 | 0.3 | 0 | 0 | 0 |

## Lithuania

| **Country** | **Population** | **HBsAg endemicity** | **HBsAg Prevalence** | | | **Estimated number of CHB cases** | | |
| --- | --- | --- | --- | --- | --- | --- | --- | --- |
|  |  |  | **%** | **LL 95%CI** | **UL 95%CI** | **Cases** | **Lower** | **Upper** |
| **Russia** | 62,241 | int-high | 2.89 | 2.16 | 3.62 | 1,799 | 1,344 | 2,253 |
| **Belarus** | 37,079 | int-high | 3.19 | 1.72 | 4.65 | 1,183 | 638 | 1,724 |
| **Ukraine** | 13,035 | int-high | 2.2 | 1.15 | 3.24 | 287 | 150 | 422 |
| **Kazakhstan** | 4,710 | int-high | 4.95 | 3.34 | 6.56 | 233 | 157 | 309 |
| **Uzbekistan** | 1,021 | int-high | 6.34 | 4.22 | 8.46 | 65 | 43 | 86 |
| **Moldova** | 637 | int-high | 9.61 | 6.92 | 12.29 | 61 | 44 | 78 |
| **Azerbaijan** | 924 | int-high | 3.11 | 1.39 | 4.84 | 29 | 13 | 45 |
| **Georgia** | 700 | int-high | 3.89 | 1.25 | 6.54 | 27 | 9 | 46 |
| **Kyrgyzstan** | 405 | int-high | 3.61 | 3.05 | 4.19 | 15 | 12 | 17 |
| **China** | 123 | int-high | 10.23 | 9.35 | 11.11 | 13 | 12 | 14 |
| **Tajikistan** | 344 | int-high | 3.61 | 3.05 | 4.19 | 12 | 10 | 14 |
| **Turkmenistan** | 257 | int-high | 3.61 | 3.05 | 4.19 | 9 | 8 | 11 |
| **Romania** | 149 | int-high | 5.49 | 5.24 | 5.73 | 8 | 8 | 9 |
| **Mongolia** | 67 | int-high | 8.97 | 8.47 | 9.48 | 6 | 6 | 6 |
| **Nigeria** | 39 | int-high | 13.31 | 11.57 | 15.06 | 5 | 5 | 6 |
| **Vietnam** | 36 | int-high | 12.48 | 11.46 | 13.5 | 4 | 4 | 5 |
| **Turkey** | 94 | int-high | 4.29 | 3.7 | 4.88 | 4 | 3 | 5 |
| **Bulgaria** | 56 | int-high | 4.25 | 2.8 | 5.7 | 2 | 2 | 3 |
| **Egypt** | 35 | int-high | 4.18 | 1.85 | 6.51 | 1 | 1 | 2 |
| **Greece** | 40 | int-high | 2.33 | 1.54 | 3.11 | 1 | 1 | 1 |
| **Latvia** | 5,765 | low | 1.39 | 1.1 | 1.67 | 80 | 63 | 96 |
| **Poland** | 2,369 | low | 1.44 | 1.16 | 1.72 | 34 | 27 | 41 |
| **United Kingdom** | 2,199 | low | 0.54 | 0.3 | 0.6 | 12 | 7 | 13 |
| **Germany** | 1,429 | low | 0.6 | 0.4 | 0.8 | 9 | 6 | 11 |
| **Estonia** | 832 | low | 0.58 | 0.42 | 0.74 | 5 | 3 | 6 |
| **Italy** | 226 | low | 1.89 | 1.26 | 2.52 | 4 | 3 | 6 |
| **Armenia** | 590 | low | 0.57 | 0.19 | 0.94 | 3 | 1 | 6 |
| **Spain** | 473 | low | 0.66 | 0.34 | 0.97 | 3 | 2 | 5 |
| **Norway** | 535 | low | 0.55 | 0.34 | 0.71 | 3 | 2 | 4 |
| **United States** | 727 | low | 0.27 | 0.2 | 0.34 | 2 | 1 | 2 |
| **France** | 260 | low | 0.68 | 0.44 | 1.05 | 2 | 1 | 3 |
| **Denmark** | 236 | low | 0.55 | 0.34 | 0.71 | 1 | 1 | 2 |
| **Belgium** | 156 | low | 0.7 | 0.4 | 1.2 | 1 | 1 | 2 |
| **Hungary** | 98 | low | 1.08 | 0.04 | 2.11 | 1 | 0 | 2 |
| **Brasil** | 57 | low | 1.78 | 1.54 | 2.02 | 1 | 1 | 1 |
| **Ireland** | 970 | low | 0.1 | 0 | 0.3 | 1 | 0 | 3 |
| **Lebanon** | 47 | low | 1.66 | 0.86 | 2.46 | 1 | 0 | 1 |
| **Argentina** | 61 | low | 1 | 0.39 | 1.62 | 1 | 0 | 1 |
| **Portugal** | 44 | low | 1.35 | 0.66 | 2.04 | 1 | 0 | 1 |
| **Czech Republic** | 70 | low | 0.7 | 0.43 | 0.98 | 0 | 0 | 1 |
| **Israel** | 33 | low | 1.26 | 0.97 | 1.55 | 0 | 0 | 1 |
| **Sweden** | 184 | low | 0.2 | 0.1 | 0.4 | 0 | 0 | 1 |
| **Australia** | 35 | low | 0.87 | 0.39 | 1.35 | 0 | 0 | 0 |
| **Austria** | 49 | low | 0.55 | 0.34 | 0.71 | 0 | 0 | 0 |
| **Canada** | 37 | low | 0.7 | 0.6 | 0.9 | 0 | 0 | 0 |
| **Iceland** | 36 | low | 0.55 | 0.34 | 0.71 | 0 | 0 | 0 |
| **Switzerland** | 33 | low | 0.55 | 0.34 | 0.71 | 0 | 0 | 0 |
| **Finland** | 83 | low | 0.2 | 0.1 | 0.4 | 0 | 0 | 0 |
| **Netherlands** | 86 | low | 0.1 | 0 | 0.2 | 0 | 0 | 0 |

## Luxembourg

| **Country** | **Population** | **HBsAg endemicity** | **HBsAg Prevalence** | | | **Estimated number of CHB cases** | | |
| --- | --- | --- | --- | --- | --- | --- | --- | --- |
|  |  |  | **%** | **LL 95%CI** | **UL 95%CI** | **Cases** | **Lower** | **Upper** |
| **Cape Verde** | 4,622 | int-high | 5.65 | 0.16 | 11.14 | 261 | 7 | 515 |
| **China** | 1,869 | int-high | 10.23 | 9.35 | 11.11 | 191 | 175 | 208 |
| **Romania** | 1,927 | int-high | 5.49 | 5.24 | 5.73 | 106 | 101 | 110 |
| **Montenegro** | 2,847 | int-high | 3.29 | 2.33 | 4.24 | 94 | 66 | 121 |
| **Bosnia and Herzegovina** | 2,232 | int-high | 3.63 | 2.26 | 5 | 81 | 50 | 112 |
| **Angola** | 689 | int-high | 11.44 | 8.46 | 14.43 | 79 | 58 | 99 |
| **Congo** | 635 | int-high | 11.44 | 8.46 | 14.43 | 73 | 54 | 92 |
| **Guinea-Bissau** | 439 | int-high | 13.17 | 10.18 | 16.17 | 58 | 45 | 71 |
| **Serbia** | 1,754 | int-high | 3.29 | 2.33 | 4.24 | 58 | 41 | 74 |
| **Cameroon** | 481 | int-high | 11.44 | 8.46 | 14.43 | 55 | 41 | 69 |
| **Albania** | 480 | int-high | 9 | 8.1 | 9.8 | 43 | 39 | 47 |
| **Russia** | 1,278 | int-high | 2.89 | 2.16 | 3.62 | 37 | 28 | 46 |
| **Philippines** | 498 | int-high | 7.36 | 6.32 | 8.39 | 37 | 31 | 42 |
| **Bulgaria** | 807 | int-high | 4.25 | 2.8 | 5.7 | 34 | 23 | 46 |
| **Korea** | 739 | int-high | 4.33 | 3.94 | 4.73 | 32 | 29 | 35 |
| **Former Yugoslavia** | 770 | int-high | 3.98 | 1.32 | 6.64 | 31 | 10 | 51 |
| **India** | 880 | int-high | 3.23 | 2.92 | 3.55 | 28 | 26 | 31 |
| **Greece** | 1,209 | int-high | 2.33 | 1.54 | 3.11 | 28 | 19 | 38 |
| **Turkey** | 585 | int-high | 4.29 | 3.7 | 4.88 | 25 | 22 | 29 |
| **Tunisia** | 493 | int-high | 4.9 | 1.51 | 5.89 | 24 | 7 | 29 |
| **Iran** | 666 | int-high | 3.1 | 2.69 | 3.5 | 21 | 18 | 23 |
| **Algeria** | 657 | int-high | 2.6 | 0 | 12.19 | 17 | 0 | 80 |
| **FYR Macedonia** | 445 | int-high | 3.29 | 2.33 | 4.24 | 15 | 10 | 19 |
| **Ukraine** | 578 | int-high | 2.2 | 1.15 | 3.24 | 13 | 7 | 19 |
| **Lithuania** | 505 | int-high | 2.03 | 1.37 | 2.69 | 10 | 7 | 14 |
| **Portugal** | 60,897 | low | 1.35 | 0.66 | 2.04 | 822 | 402 | 1,242 |
| **Italy** | 13,223 | low | 1.89 | 1.26 | 2.52 | 250 | 167 | 333 |
| **France** | 28,080 | low | 0.68 | 0.44 | 1.05 | 191 | 124 | 295 |
| **Belgium** | 16,789 | low | 0.7 | 0.4 | 1.2 | 118 | 67 | 201 |
| **Germany** | 14,803 | low | 0.6 | 0.4 | 0.8 | 89 | 59 | 118 |
| **Poland** | 2,882 | low | 1.44 | 1.16 | 1.72 | 42 | 33 | 50 |
| **Brazil** | 1,795 | low | 1.78 | 1.54 | 2.02 | 32 | 28 | 36 |
| **United Kingdom** | 4,223 | low | 0.54 | 0.3 | 0.6 | 23 | 13 | 25 |
| **Morocco** | 1,151 | low | 1.8 | 1.51 | 5.89 | 21 | 17 | 68 |
| **Spain** | 2,914 | low | 0.66 | 0.34 | 0.97 | 19 | 10 | 28 |
| **Hungary** | 971 | low | 1.08 | 0.04 | 2.11 | 10 | 0 | 20 |
| **Denmark** | 1,519 | low | 0.55 | 0.34 | 0.71 | 8 | 5 | 11 |
| **Switzerland** | 968 | low | 0.55 | 0.34 | 0.71 | 5 | 3 | 7 |
| **Austria** | 815 | low | 0.55 | 0.34 | 0.71 | 4 | 3 | 6 |
| **United States** | 1,626 | low | 0.27 | 0.2 | 0.34 | 4 | 3 | 6 |
| **Slovak Republic** | 576 | low | 0.7 | 0.43 | 0.98 | 4 | 2 | 6 |
| **Canada** | 505 | low | 0.7 | 0.6 | 0.9 | 4 | 3 | 5 |
| **Netherlands** | 3,503 | low | 0.1 | 0 | 0.2 | 4 | 0 | 7 |
| **Czech Republic** | 444 | low | 0.7 | 0.43 | 0.98 | 3 | 2 | 4 |
| **Sweden** | 1,374 | low | 0.2 | 0.1 | 0.4 | 3 | 1 | 5 |
| **Estonia** | 436 | low | 0.58 | 0.42 | 0.74 | 3 | 2 | 3 |
| **Iceland** | 434 | low | 0.55 | 0.34 | 0.71 | 2 | 1 | 3 |
| **Finland** | 908 | low | 0.2 | 0.1 | 0.4 | 2 | 1 | 4 |
| **Ireland** | 937 | low | 0.1 | 0 | 0.3 | 1 | 0 | 3 |

## Latvia

| **Country** | **Population** | **HBsAg endemicity** | **HBsAg Prevalence** | | | **Estimated number of CHB cases** | | |
| --- | --- | --- | --- | --- | --- | --- | --- | --- |
|  |  |  | **%** | **LL 95%CI** | **UL 95%CI** | **Cases** | **Lower** | **Upper** |
| **Russia** | 146,280 | int-high | 2.89 | 2.16 | 3.62 | 4,227 | 3,160 | 5,295 |
| **Belarus** | 51,500 | int-high | 3.19 | 1.72 | 4.65 | 1,643 | 886 | 2,395 |
| **Ukraine** | 35,687 | int-high | 2.2 | 1.15 | 3.24 | 785 | 410 | 1,156 |
| **Lithuania** | 17,854 | int-high | 2.03 | 1.37 | 2.69 | 362 | 245 | 480 |
| **Kazakhstan** | 6,202 | int-high | 4.95 | 3.34 | 6.56 | 307 | 207 | 407 |
| **Moldova** | 1,752 | int-high | 9.61 | 6.92 | 12.29 | 168 | 121 | 215 |
| **Uzbekistan** | 2,018 | int-high | 6.34 | 4.22 | 8.46 | 128 | 85 | 171 |
| **Azerbaijan** | 2,012 | int-high | 3.11 | 1.39 | 4.84 | 63 | 28 | 97 |
| **Georgia** | 1,382 | int-high | 3.89 | 1.25 | 6.54 | 54 | 17 | 90 |
| **Kyrgyzstan** | 866 | int-high | 3.61 | 3.05 | 4.19 | 31 | 26 | 36 |
| **Turkmenistan** | 622 | int-high | 3.61 | 3.05 | 4.19 | 22 | 19 | 26 |
| **China** | 212 | int-high | 10.23 | 9.35 | 11.11 | 22 | 20 | 24 |
| **Tajikistan** | 547 | int-high | 3.61 | 3.05 | 4.19 | 20 | 17 | 23 |
| **Mongolia** | 115 | int-high | 8.97 | 8.47 | 9.48 | 10 | 10 | 11 |
| **Bulgaria** | 148 | int-high | 4.25 | 2.8 | 5.7 | 6 | 4 | 8 |
| **Turkey** | 102 | int-high | 4.29 | 3.7 | 4.88 | 4 | 4 | 5 |
| **Romania** | 71 | int-high | 5.49 | 5.24 | 5.73 | 4 | 4 | 4 |
| **India** | 97 | int-high | 3.23 | 2.92 | 3.55 | 3 | 3 | 3 |
| **Egypt** | 28 | int-high | 4.18 | 1.85 | 6.51 | 1 | 1 | 2 |
| **Pakistan** | 22 | int-high | 4.17 | 3.59 | 4.75 | 1 | 1 | 1 |
| **South Korea** | 21 | int-high | 4.33 | 3.94 | 4.73 | 1 | 1 | 1 |
| **Greece** | 34 | int-high | 2.33 | 1.54 | 3.11 | 1 | 1 | 1 |
| **Iran** | 25 | int-high | 3.1 | 2.69 | 3.5 | 1 | 1 | 1 |
| **Nepal** | 20 | int-high | 2.32 | 1.71 | 2.93 | 0 | 0 | 1 |
| **Estonia** | 3,148 | low | 0.58 | 0.42 | 0.74 | 18 | 13 | 23 |
| **Germany** | 2,112 | low | 0.6 | 0.4 | 0.8 | 13 | 8 | 17 |
| **Poland** | 879 | low | 1.44 | 1.16 | 1.72 | 13 | 10 | 15 |
| **United Kingdom** | 1,234 | low | 0.54 | 0.3 | 0.6 | 7 | 4 | 7 |
| **Armenia** | 737 | low | 0.57 | 0.19 | 0.94 | 4 | 1 | 7 |
| **Italy** | 189 | low | 1.89 | 1.26 | 2.52 | 4 | 2 | 5 |
| **Hungary** | 201 | low | 1.08 | 0.04 | 2.11 | 2 | 0 | 4 |
| **France** | 171 | low | 0.68 | 0.44 | 1.05 | 1 | 1 | 2 |
| **United States** | 402 | low | 0.27 | 0.2 | 0.34 | 1 | 1 | 1 |
| **Czech Republic** | 137 | low | 0.7 | 0.43 | 0.98 | 1 | 1 | 1 |
| **Denmark** | 169 | low | 0.55 | 0.34 | 0.71 | 1 | 1 | 1 |
| **Israel** | 70 | low | 1.26 | 0.97 | 1.55 | 1 | 1 | 1 |
| **Australia** | 91 | low | 0.87 | 0.39 | 1.35 | 1 | 0 | 1 |
| **Canada** | 108 | low | 0.7 | 0.6 | 0.9 | 1 | 1 | 1 |
| **Norway** | 130 | low | 0.55 | 0.34 | 0.71 | 1 | 0 | 1 |
| **Spain** | 106 | low | 0.66 | 0.34 | 0.97 | 1 | 0 | 1 |
| **Belgium** | 93 | low | 0.7 | 0.4 | 1.2 | 1 | 0 | 1 |
| **Austria** | 84 | low | 0.55 | 0.34 | 0.71 | 0 | 0 | 1 |
| **Lebanon** | 26 | low | 1.66 | 0.86 | 2.46 | 0 | 0 | 1 |
| **Venezuela** | 22 | low | 1.93 | 1.13 | 2.73 | 0 | 0 | 1 |
| **Cuba** | 47 | low | 0.86 | 0.58 | 1.14 | 0 | 0 | 1 |
| **Sweden** | 193 | low | 0.2 | 0.1 | 0.4 | 0 | 0 | 1 |
| **Finland** | 130 | low | 0.2 | 0.1 | 0.4 | 0 | 0 | 1 |
| **Argentina** | 19 | low | 1 | 0.39 | 1.62 | 0 | 0 | 0 |
| **Slovakia** | 22 | low | 0.7 | 0.43 | 0.98 | 0 | 0 | 0 |
| **Netherlands** | 106 | low | 0.1 | 0 | 0.2 | 0 | 0 | 0 |

## Malta

| **Country** | **Population** | **HBsAg endemicity** | **HBsAg Prevalence** | | | **Estimated number of CHB cases** | | |
| --- | --- | --- | --- | --- | --- | --- | --- | --- |
|  |  |  | **%** | **LL 95%CI** | **UL 95%CI** | **Cases** | **Lower** | **Upper** |
| **Somalia** | 1,003 | int-high | 12.4 | 8.89 | 15.92 | 124 | 89 | 160 |
| **Eritrea** | 507 | int-high | 15.52 | 2.02 | 29.02 | 79 | 10 | 147 |
| **Nigeria** | 330 | int-high | 13.31 | 11.57 | 15.06 | 44 | 38 | 50 |
| **Sudan** | 227 | int-high | 18.59 | 14.22 | 22.96 | 42 | 32 | 52 |
| **China** | 377 | int-high | 10.23 | 9.35 | 11.11 | 39 | 35 | 42 |
| **Bulgaria** | 875 | int-high | 4.25 | 2.8 | 5.7 | 37 | 25 | 50 |
| **Romania** | 656 | int-high | 5.49 | 5.24 | 5.73 | 36 | 34 | 38 |
| **Philippines** | 464 | int-high | 7.36 | 6.32 | 8.39 | 34 | 29 | 39 |
| **Russian Federation** | 918 | int-high | 2.89 | 2.16 | 3.62 | 27 | 20 | 33 |
| **Egypt** | 454 | int-high | 4.18 | 1.85 | 6.51 | 19 | 8 | 30 |
| **Ethiopia** | 332 | int-high | 5.47 | 2.55 | 8.39 | 18 | 8 | 28 |
| **Serbia** | 534 | int-high | 3.29 | 2.33 | 4.24 | 18 | 12 | 23 |
| **Libya** | 776 | int-high | 2.2 | 1.51 | 5.89 | 17 | 12 | 46 |
| **Tunisia** | 312 | int-high | 4.9 | 1.51 | 5.89 | 15 | 5 | 18 |
| **Syria** | 255 | int-high | 5.62 | 4.82 | 6.42 | 14 | 12 | 16 |
| **Ivory Coast** | 93 | int-high | 13.17 | 10.18 | 16.17 | 12 | 9 | 15 |
| **South Africa** | 169 | int-high | 6.2 | 4.68 | 7.71 | 10 | 8 | 13 |
| **Turkey** | 223 | int-high | 4.29 | 3.7 | 4.88 | 10 | 8 | 11 |
| **India** | 255 | int-high | 3.23 | 2.92 | 3.55 | 8 | 7 | 9 |
| **Pakistan** | 177 | int-high | 4.17 | 3.59 | 4.75 | 7 | 6 | 8 |
| **Albania** | 75 | int-high | 9 | 8.1 | 9.8 | 7 | 6 | 7 |
| **Ukraine** | 279 | int-high | 2.2 | 1.15 | 3.24 | 6 | 3 | 9 |
| **Bosnia and Herzegovina** | 159 | int-high | 3.63 | 2.26 | 5 | 6 | 4 | 8 |
| **Thailand** | 104 | int-high | 5.54 | 4.64 | 6.43 | 6 | 5 | 7 |
| **Greece** | 75 | int-high | 2.33 | 1.54 | 3.11 | 2 | 1 | 2 |
| **United Kingdom** | 10,480 | low | 0.54 | 0.3 | 0.6 | 57 | 31 | 63 |
| **Australia** | 4,354 | low | 0.87 | 0.39 | 1.35 | 38 | 17 | 59 |
| **Italy** | 1,511 | low | 1.89 | 1.26 | 2.52 | 29 | 19 | 38 |
| **Canada** | 1,766 | low | 0.7 | 0.6 | 0.9 | 12 | 11 | 16 |
| **Germany** | 951 | low | 0.6 | 0.4 | 0.8 | 6 | 4 | 8 |
| **Morocco** | 217 | low | 1.8 | 1.51 | 5.89 | 4 | 3 | 13 |
| **Poland** | 242 | low | 1.44 | 1.16 | 1.72 | 3 | 3 | 4 |
| **United States** | 1,246 | low | 0.27 | 0.2 | 0.34 | 3 | 2 | 4 |
| **France** | 417 | low | 0.68 | 0.44 | 1.05 | 3 | 2 | 4 |
| **Brazil** | 94 | low | 1.78 | 1.54 | 2.02 | 2 | 1 | 2 |
| **Hungary** | 140 | low | 1.08 | 0.04 | 2.11 | 2 | 0 | 3 |
| **Belgium** | 162 | low | 0.7 | 0.4 | 1.2 | 1 | 1 | 2 |
| **Iraq** | 81 | low | 1.31 | 0 | 2.87 | 1 | 0 | 2 |
| **Croatia** | 71 | low | 1.47 | 0.84 | 2.1 | 1 | 1 | 1 |
| **Latvia** | 75 | low | 1.39 | 1.1 | 1.67 | 1 | 1 | 1 |
| **Spain** | 147 | low | 0.66 | 0.34 | 0.97 | 1 | 0 | 1 |
| **Sweden** | 450 | low | 0.2 | 0.1 | 0.4 | 1 | 0 | 2 |
| **Denmark** | 127 | low | 0.55 | 0.34 | 0.71 | 1 | 0 | 1 |
| **Slovakia** | 99 | low | 0.7 | 0.43 | 0.98 | 1 | 0 | 1 |
| **Austria** | 108 | low | 0.55 | 0.34 | 0.71 | 1 | 0 | 1 |
| **Czech Republic** | 80 | low | 0.7 | 0.43 | 0.98 | 1 | 0 | 1 |
| **Switzerland** | 101 | low | 0.55 | 0.34 | 0.71 | 1 | 0 | 1 |
| **Netherlands** | 412 | low | 0.1 | 0 | 0.2 | 0 | 0 | 1 |
| **Ireland** | 264 | low | 0.1 | 0 | 0.3 | 0 | 0 | 1 |
| **Finland** | 77 | low | 0.2 | 0.1 | 0.4 | 0 | 0 | 0 |

## The Netherlands

| **Country** | **Population** | **HBsAg endemicity** | **HBsAg Prevalence** | | | **Estimated number of CHB cases** | | |
| --- | --- | --- | --- | --- | --- | --- | --- | --- |
|  |  |  | **%** | **LL 95%CI** | **UL 95%CI** | **Cases** | **Lower** | **Upper** |
| **Turkey** | 196,536 | int-high | 4.29 | 3.7 | 4.88 | 8,431 | 7,272 | 9,591 |
| **Suriname** | 184,098 | int-high | 4.52 | 2.47 | 6.57 | 8,321 | 4,547 | 12,095 |
| **China** | 59,759 | int-high | 10.23 | 9.35 | 11.11 | 6,113 | 5,587 | 6,639 |
| **Indonesia** | 132,044 | int-high | 3.93 | 3.08 | 4.77 | 5,189 | 4,067 | 6,298 |
| **Former Dutch Antilles** | 86,371 | int-high | 4.52 | 2.47 | 6.57 | 3,904 | 2,133 | 5,675 |
| **Afghanistan** | 32,832 | int-high | 10.46 | 5.85 | 15.07 | 3,434 | 1,921 | 4,948 |
| **Somalia** | 24,605 | int-high | 12.4 | 8.89 | 15.92 | 3,051 | 2,187 | 3,917 |
| **Former Yugoslavia** | 50,357 | int-high | 3.98 | 1.32 | 6.64 | 2,004 | 665 | 3,344 |
| **Ghana** | 13,835 | int-high | 13.44 | 10.5 | 16.38 | 1,859 | 1,453 | 2,266 |
| **Vietnam** | 12,441 | int-high | 12.48 | 11.46 | 13.5 | 1,553 | 1,426 | 1,680 |
| **Former Soviet Union** | 38,194 | int-high | 3.83 | 2.74 | 4.91 | 1,463 | 1,047 | 1,875 |
| **Nigeria** | 6,846 | int-high | 13.31 | 11.57 | 15.06 | 911 | 792 | 1,031 |
| **Dominican Republic** | 8,369 | int-high | 10.68 | 5.89 | 15.46 | 894 | 493 | 1,294 |
| **Iran** | 27,989 | int-high | 3.1 | 2.69 | 3.5 | 868 | 753 | 980 |
| **Philippines** | 11,655 | int-high | 7.36 | 6.32 | 8.39 | 858 | 737 | 978 |
| **South Africa** | 13,048 | int-high | 6.2 | 4.68 | 7.71 | 809 | 611 | 1,006 |
| **Bulgaria** | 18,191 | int-high | 4.25 | 2.8 | 5.7 | 773 | 509 | 1,037 |
| **Romania** | 13,758 | int-high | 5.49 | 5.24 | 5.73 | 755 | 721 | 788 |
| **Thailand** | 13,415 | int-high | 5.54 | 4.64 | 6.43 | 743 | 622 | 863 |
| **India** | 20,683 | int-high | 3.23 | 2.92 | 3.55 | 668 | 604 | 734 |
| **Cape Verde** | 11,691 | int-high | 5.65 | 0.16 | 11.14 | 661 | 19 | 1,302 |
| **Angola** | 5,387 | int-high | 11.44 | 8.46 | 14.43 | 616 | 456 | 777 |
| **Egypt** | 12,335 | int-high | 4.18 | 1.85 | 6.51 | 516 | 228 | 803 |
| **Ethiopia** | 9,115 | int-high | 5.47 | 2.55 | 8.39 | 499 | 232 | 765 |
| **Pakistan** | 11,620 | int-high | 4.17 | 3.59 | 4.75 | 485 | 417 | 552 |
| **Syria** | 7,673 | int-high | 5.62 | 4.82 | 6.42 | 431 | 370 | 493 |
| **Greece** | 12,810 | int-high | 2.33 | 1.54 | 3.11 | 298 | 197 | 398 |
| **South Korea** | 6,845 | int-high | 4.33 | 3.94 | 4.73 | 296 | 270 | 324 |
| **Sri Lanka** | 10,193 | int-high | 2.41 | 0 | 5.53 | 246 | 0 | 564 |
| **Morocco** | 168,236 | low | 1.8 | 1.51 | 5.89 | 3,028 | 2,540 | 9,909 |
| **Poland** | 86,541 | low | 1.44 | 1.16 | 1.72 | 1,246 | 1,004 | 1,489 |
| **Germany** | 121,823 | low | 0.6 | 0.4 | 0.8 | 731 | 487 | 975 |
| **Iraq** | 40,562 | low | 1.31 | 0 | 2.87 | 531 | 0 | 1,164 |
| **Italy** | 22,519 | low | 1.89 | 1.26 | 2.52 | 426 | 284 | 567 |
| **Belgium** | 51,942 | low | 0.7 | 0.4 | 1.2 | 364 | 208 | 623 |
| **Brazil** | 15,035 | low | 1.78 | 1.54 | 2.02 | 268 | 232 | 304 |
| **UK** | 47,789 | low | 0.54 | 0.3 | 0.6 | 258 | 143 | 287 |
| **Portugal** | 15,795 | low | 1.35 | 0.66 | 2.04 | 213 | 104 | 322 |
| **Colombia** | 13,718 | low | 1.2 | 0.27 | 2.13 | 165 | 37 | 292 |
| **France** | 22,940 | low | 0.68 | 0.44 | 1.05 | 156 | 101 | 241 |
| **Spain** | 22,576 | low | 0.66 | 0.34 | 0.97 | 149 | 77 | 219 |
| **Hungary** | 11,836 | low | 1.08 | 0.04 | 2.11 | 128 | 5 | 250 |
| **Australia** | 10,049 | low | 0.87 | 0.39 | 1.35 | 87 | 39 | 136 |
| **United States** | 26,282 | low | 0.27 | 0.2 | 0.34 | 71 | 53 | 89 |
| **Canada** | 9,542 | low | 0.7 | 0.6 | 0.9 | 67 | 57 | 86 |
| **Israel** | 5,164 | low | 1.26 | 0.97 | 1.55 | 65 | 50 | 80 |
| **Czech Republic and Slovakia** | 8,688 | low | 0.7 | 0.43 | 0.98 | 61 | 37 | 85 |
| **Japan** | 5,826 | low | 0.63 | 0.6 | 0.7 | 37 | 35 | 41 |
| **Switzerland** | 6,637 | low | 0.55 | 0.34 | 0.71 | 37 | 23 | 47 |
| **Austria** | 6,561 | low | 0.55 | 0.34 | 0.71 | 36 | 22 | 47 |

## Norway

| **Country** | **Population** | **HBsAg endemicity** | **HBsAg Prevalence** | | | **Estimated number of CHB cases** | | |
| --- | --- | --- | --- | --- | --- | --- | --- | --- |
|  |  |  | **%** | **LL 95%CI** | **UL 95%CI** | **Cases** | **Lower** | **Upper** |
| **Somalia** | 23,670 | int-high | 12.4 | 8.89 | 15.92 | 2,935 | 2,104 | 3,768 |
| **Vietnam** | 13,481 | int-high | 12.48 | 11.46 | 13.5 | 1,682 | 1,545 | 1,820 |
| **Eritrea** | 10,097 | int-high | 15.52 | 2.02 | 29.02 | 1,567 | 204 | 2,930 |
| **Philippines** | 17,586 | int-high | 7.36 | 6.32 | 8.39 | 1,294 | 1,111 | 1,475 |
| **Afghanistan** | 11,980 | int-high | 10.46 | 5.85 | 15.07 | 1,253 | 701 | 1,805 |
| **China** | 11,450 | int-high | 10.23 | 9.35 | 11.11 | 1,171 | 1,071 | 1,272 |
| **Thailand** | 16,322 | int-high | 5.54 | 4.64 | 6.43 | 904 | 757 | 1,050 |
| **Pakistan** | 18,543 | int-high | 4.17 | 3.59 | 4.75 | 773 | 666 | 881 |
| **Lithuania** | 28,610 | int-high | 2.03 | 1.37 | 2.69 | 581 | 392 | 770 |
| **Bosnia and Herzegovina** | 13,110 | int-high | 3.63 | 2.26 | 5 | 476 | 296 | 656 |
| **Iran** | 15,121 | int-high | 3.1 | 2.69 | 3.5 | 469 | 407 | 529 |
| **Turkey** | 10,860 | int-high | 4.29 | 3.7 | 4.88 | 466 | 402 | 530 |
| **Russia** | 16,107 | int-high | 2.89 | 2.16 | 3.62 | 465 | 348 | 583 |
| **Romania** | 8,118 | int-high | 5.49 | 5.24 | 5.73 | 446 | 425 | 465 |
| **Ethiopia** | 6,689 | int-high | 5.47 | 2.55 | 8.39 | 366 | 171 | 561 |
| **Myanmar (Burma)** | 3,072 | int-high | 11.63 | 9.53 | 13.73 | 357 | 293 | 422 |
| **India** | 10,085 | int-high | 3.23 | 2.92 | 3.55 | 326 | 294 | 358 |
| **South Korea** | 7,311 | int-high | 4.33 | 3.94 | 4.73 | 317 | 288 | 346 |
| **Kosovo** | 9,603 | int-high | 3.29 | 2.33 | 4.24 | 316 | 224 | 407 |
| **Sri Lanka** | 9,088 | int-high | 2.41 | 0 | 5.53 | 219 | 0 | 503 |
| **Palestine** | 3,195 | int-high | 6.62 | 4.6 | 8.64 | 212 | 147 | 276 |
| **Bulgaria** | 3,970 | int-high | 4.25 | 2.8 | 5.7 | 169 | 111 | 226 |
| **Serbia** | 3,128 | int-high | 3.29 | 2.33 | 4.24 | 103 | 73 | 133 |
| **Ukraine** | 3,515 | int-high | 2.2 | 1.15 | 3.24 | 77 | 40 | 114 |
| **Macedonia** | 2,336 | int-high | 3.29 | 2.33 | 4.24 | 77 | 54 | 99 |
| **Poland** | 76,723 | low | 1.44 | 1.16 | 1.72 | 1,105 | 890 | 1,320 |
| **Iraq** | 22,113 | low | 1.31 | 0 | 2.87 | 290 | 0 | 635 |
| **Germany** | 27,786 | low | 0.6 | 0.4 | 0.8 | 167 | 111 | 222 |
| **Denmark** | 23,811 | low | 0.55 | 0.34 | 0.71 | 131 | 81 | 169 |
| **Latvia** | 8,117 | low | 1.39 | 1.1 | 1.67 | 113 | 89 | 136 |
| **United Kingdom** | 18,615 | low | 0.54 | 0.3 | 0.6 | 101 | 56 | 112 |
| **Sweden** | 47,873 | low | 0.2 | 0.1 | 0.4 | 96 | 48 | 191 |
| **Morocco** | 5,249 | low | 1.8 | 1.51 | 5.89 | 94 | 79 | 309 |
| **Brazil** | 4,476 | low | 1.78 | 1.54 | 2.02 | 80 | 69 | 90 |
| **Colombia** | 5,360 | low | 1.2 | 0.27 | 2.13 | 64 | 14 | 114 |
| **Italy** | 3,178 | low | 1.89 | 1.26 | 2.52 | 60 | 40 | 80 |
| **United States** | 16,983 | low | 0.27 | 0.2 | 0.34 | 46 | 34 | 58 |
| **Croatia** | 3,007 | low | 1.47 | 0.84 | 2.1 | 44 | 25 | 63 |
| **Iceland** | 7,497 | low | 0.55 | 0.34 | 0.71 | 41 | 25 | 53 |
| **France** | 5,721 | low | 0.68 | 0.44 | 1.05 | 39 | 25 | 60 |
| **Hungary** | 3,164 | low | 1.08 | 0.04 | 2.11 | 34 | 1 | 67 |
| **Spain** | 4,594 | low | 0.66 | 0.34 | 0.97 | 30 | 16 | 45 |
| **Portugal** | 1,961 | low | 1.35 | 0.66 | 2.04 | 26 | 13 | 40 |
| **Chile** | 6,547 | low | 0.4 | 0.11 | 0.69 | 26 | 7 | 45 |
| **Estonia** | 4,408 | low | 0.58 | 0.42 | 0.74 | 26 | 19 | 33 |
| **Slovakia** | 3,240 | low | 0.7 | 0.43 | 0.98 | 23 | 14 | 32 |
| **Canada** | 2,969 | low | 0.7 | 0.6 | 0.9 | 21 | 18 | 27 |
| **Finland** | 6,648 | low | 0.2 | 0.1 | 0.4 | 13 | 7 | 27 |
| **Switzerland** | 2,101 | low | 0.55 | 0.34 | 0.71 | 12 | 7 | 15 |
| **Netherlands** | 8,128 | low | 0.1 | 0 | 0.2 | 8 | 0 | 16 |

## Poland

| **Country** | **Population** | **HBsAg endemicity** | **HBsAg Prevalence** | | | **Estimated number of CHB cases** | | |
| --- | --- | --- | --- | --- | --- | --- | --- | --- |
|  |  |  | **%** | **LL 95%CI** | **UL 95%CI** | **Cases** | **Lower** | **Upper** |
| **Ukraine** | 227,995 | int-high | 2.2 | 1.15 | 3.24 | 5,016 | 2,622 | 7,387 |
| **Belarus** | 83,842 | int-high | 3.19 | 1.72 | 4.65 | 2,675 | 1,442 | 3,899 |
| **Russia** | 41,344 | int-high | 2.89 | 2.16 | 3.62 | 1,195 | 893 | 1,497 |
| **Lithuania** | 55,592 | int-high | 2.03 | 1.37 | 2.69 | 1,129 | 762 | 1,495 |
| **Vietnam** | 3,112 | int-high | 12.48 | 11.46 | 13.5 | 388 | 357 | 420 |
| **Kazakhstan** | 5,128 | int-high | 4.95 | 3.34 | 6.56 | 254 | 171 | 336 |
| **Romania** | 2,744 | int-high | 5.49 | 5.24 | 5.73 | 151 | 144 | 157 |
| **Bosnia and Herzegovina** | 3,819 | int-high | 3.63 | 2.26 | 5 | 139 | 86 | 191 |
| **China** | 1,273 | int-high | 10.23 | 9.35 | 11.11 | 130 | 119 | 141 |
| **Bulgaria** | 1,921 | int-high | 4.25 | 2.8 | 5.7 | 82 | 54 | 109 |
| **Greece** | 3,491 | int-high | 2.33 | 1.54 | 3.11 | 81 | 54 | 109 |
| **Moldova** | 629 | int-high | 9.61 | 6.92 | 12.29 | 60 | 44 | 77 |
| **Nigeria** | 449 | int-high | 13.31 | 11.57 | 15.06 | 60 | 52 | 68 |
| **Turkey** | 1,014 | int-high | 4.29 | 3.7 | 4.88 | 44 | 38 | 49 |
| **Syria** | 661 | int-high | 5.62 | 4.82 | 6.42 | 37 | 32 | 42 |
| **Uzbekistan** | 557 | int-high | 6.34 | 4.22 | 8.46 | 35 | 24 | 47 |
| **Serbia** | 1,032 | int-high | 3.29 | 2.33 | 4.24 | 34 | 24 | 44 |
| **Mongolia** | 371 | int-high | 8.97 | 8.47 | 9.48 | 33 | 31 | 35 |
| **India** | 973 | int-high | 3.23 | 2.92 | 3.55 | 31 | 28 | 35 |
| **South Africa** | 434 | int-high | 6.2 | 4.68 | 7.71 | 27 | 20 | 33 |
| **Tunisia** | 484 | int-high | 4.9 | 1.51 | 5.89 | 24 | 7 | 29 |
| **Egypt** | 554 | int-high | 4.18 | 1.85 | 6.51 | 23 | 10 | 36 |
| **Algeria** | 657 | int-high | 2.6 | 0 | 12.19 | 17 | 0 | 80 |
| **Georgia** | 370 | int-high | 3.89 | 1.25 | 6.54 | 14 | 5 | 24 |
| **Germany** | 84,336 | low | 0.6 | 0.4 | 0.8 | 506 | 337 | 675 |
| **Italy** | 11,122 | low | 1.89 | 1.26 | 2.52 | 210 | 140 | 280 |
| **United Kingdom** | 38,517 | low | 0.54 | 0.3 | 0.6 | 208 | 116 | 231 |
| **France** | 28,878 | low | 0.68 | 0.44 | 1.05 | 196 | 127 | 303 |
| **United States** | 15,378 | low | 0.27 | 0.2 | 0.34 | 42 | 31 | 52 |
| **Belgium** | 5,799 | low | 0.7 | 0.4 | 1.2 | 41 | 23 | 70 |
| **Spain** | 5,457 | low | 0.66 | 0.34 | 0.97 | 36 | 19 | 53 |
| **Czech Republic** | 4,987 | low | 0.7 | 0.43 | 0.98 | 35 | 21 | 49 |
| **Latvia** | 1,718 | low | 1.39 | 1.1 | 1.67 | 24 | 19 | 29 |
| **Austria** | 4,254 | low | 0.55 | 0.34 | 0.71 | 23 | 14 | 30 |
| **Canada** | 3,329 | low | 0.7 | 0.6 | 0.9 | 23 | 20 | 30 |
| **Hungary** | 1,492 | low | 1.08 | 0.04 | 2.11 | 16 | 1 | 31 |
| **Armenia** | 2,384 | low | 0.57 | 0.19 | 0.94 | 14 | 5 | 22 |
| **Croatia** | 870 | low | 1.47 | 0.84 | 2.1 | 13 | 7 | 18 |
| **Slovakia** | 1,438 | low | 0.7 | 0.43 | 0.98 | 10 | 6 | 14 |
| **Norway** | 1,784 | low | 0.55 | 0.34 | 0.71 | 10 | 6 | 13 |
| **Australia** | 1,035 | low | 0.87 | 0.39 | 1.35 | 9 | 4 | 14 |
| **Lebanon** | 433 | low | 1.66 | 0.86 | 2.46 | 7 | 4 | 11 |
| **Morocco** | 364 | low | 1.8 | 1.51 | 5.89 | 7 | 5 | 21 |
| **Switzerland** | 1,157 | low | 0.55 | 0.34 | 0.71 | 6 | 4 | 8 |
| **Iraq** | 477 | low | 1.31 | 0 | 2.87 | 6 | 0 | 14 |
| **Denmark** | 994 | low | 0.55 | 0.34 | 0.71 | 5 | 3 | 7 |
| **Japan** | 576 | low | 0.63 | 0.6 | 0.7 | 4 | 3 | 4 |
| **Sweden** | 1,512 | low | 0.2 | 0.1 | 0.4 | 3 | 2 | 6 |
| **Netherlands** | 2,920 | low | 0.1 | 0 | 0.2 | 3 | 0 | 6 |

## Portugal

| **Country** | **Population** | **HBsAg endemicity** | **HBsAg Prevalence** | | | **Estimated number of CHB cases** | | |
| --- | --- | --- | --- | --- | --- | --- | --- | --- |
|  |  |  | **%** | **LL 95%CI** | **UL 95%CI** | **Cases** | **Lower** | **Upper** |
| **Angola** | 162,604 | int-high | 11.44 | 8.46 | 14.43 | 18,602 | 13,756 | 23,464 |
| **Mozambique** | 73,084 | int-high | 9.66 | 7.06 | 12.26 | 7,060 | 5,160 | 8,960 |
| **Guinea-Bissau** | 29,578 | int-high | 13.17 | 10.18 | 16.17 | 3,895 | 3,011 | 4,783 |
| **Cape Verde** | 61,953 | int-high | 5.65 | 0.16 | 11.14 | 3,500 | 99 | 6,902 |
| **Sao Tome and Principe** | 18,645 | int-high | 13.17 | 10.18 | 16.17 | 2,456 | 1,898 | 3,015 |
| **Moldova** | 14,324 | int-high | 9.61 | 6.92 | 12.29 | 1,377 | 991 | 1,760 |
| **Romania** | 23,689 | int-high | 5.49 | 5.24 | 5.73 | 1,301 | 1,241 | 1,357 |
| **China** | 10,887 | int-high | 10.23 | 9.35 | 11.11 | 1,114 | 1,018 | 1,210 |
| **Ukraine** | 33,172 | int-high | 2.2 | 1.15 | 3.24 | 730 | 381 | 1,075 |
| **South Africa** | 11,477 | int-high | 6.2 | 4.68 | 7.71 | 712 | 537 | 885 |
| **Congo** | 2,332 | int-high | 11.44 | 8.46 | 14.43 | 267 | 197 | 337 |
| **India** | 8,129 | int-high | 3.23 | 2.92 | 3.55 | 263 | 237 | 289 |
| **Bulgaria** | 5,391 | int-high | 4.25 | 2.8 | 5.7 | 229 | 151 | 307 |
| **Zimbabwe** | 1,478 | int-high | 13.91 | 10.7 | 17.11 | 206 | 158 | 253 |
| **Senegal** | 1,374 | int-high | 12.66 | 10.14 | 15.18 | 174 | 139 | 209 |
| **Russia** | 5,906 | int-high | 2.89 | 2.16 | 3.62 | 171 | 128 | 214 |
| **Guinea** | 733 | int-high | 16.33 | 14.61 | 18.05 | 120 | 107 | 132 |
| **DRC Congo** | 833 | int-high | 11.44 | 8.46 | 14.43 | 95 | 70 | 120 |
| **Pakistan** | 2,015 | int-high | 4.17 | 3.59 | 4.75 | 84 | 72 | 96 |
| **Timor-Leste** | 1,789 | int-high | 3.93 | 3.08 | 4.77 | 70 | 55 | 85 |
| **Bangladesh** | 1,080 | int-high | 4.83 | 4.02 | 5.64 | 52 | 43 | 61 |
| **Uzbekistan** | 705 | int-high | 6.34 | 4.22 | 8.46 | 45 | 30 | 60 |
| **Kazakhstan** | 714 | int-high | 4.95 | 3.34 | 6.56 | 35 | 24 | 47 |
| **Philippines** | 474 | int-high | 7.36 | 6.32 | 8.39 | 35 | 30 | 40 |
| **Georgia** | 773 | int-high | 3.89 | 1.25 | 6.54 | 30 | 10 | 51 |
| **Thailand** | 491 | int-high | 5.54 | 4.64 | 6.43 | 27 | 23 | 32 |
| **Nepal** | 971 | int-high | 2.32 | 1.71 | 2.93 | 23 | 17 | 28 |
| **Belarus** | 554 | int-high | 3.19 | 1.72 | 4.65 | 18 | 10 | 26 |
| **Brazil** | 139,703 | low | 1.78 | 1.54 | 2.02 | 2,487 | 2,151 | 2,822 |
| **France** | 94,484 | low | 0.68 | 0.44 | 1.05 | 642 | 416 | 992 |
| **Venezuela** | 25,157 | low | 1.93 | 1.13 | 2.73 | 486 | 284 | 687 |
| **Germany** | 28,000 | low | 0.6 | 0.4 | 0.8 | 168 | 112 | 224 |
| **Spain** | 16,489 | low | 0.66 | 0.34 | 0.97 | 109 | 56 | 160 |
| **United Kingdom** | 19,131 | low | 0.54 | 0.3 | 0.6 | 103 | 57 | 115 |
| **Switzerland** | 16,482 | low | 0.55 | 0.34 | 0.71 | 91 | 56 | 117 |
| **Italy** | 2,837 | low | 1.89 | 1.26 | 2.52 | 54 | 36 | 71 |
| **Canada** | 7,572 | low | 0.7 | 0.6 | 0.9 | 53 | 45 | 68 |
| **Morocco** | 2,436 | low | 1.8 | 1.51 | 5.89 | 44 | 37 | 143 |
| **Belgium** | 3,560 | low | 0.7 | 0.4 | 1.2 | 25 | 14 | 43 |
| **Luxembourg** | 4,086 | low | 0.55 | 0.34 | 0.71 | 22 | 14 | 29 |
| **United States** | 7,242 | low | 0.27 | 0.2 | 0.34 | 20 | 14 | 25 |
| **Poland** | 1,014 | low | 1.44 | 1.16 | 1.72 | 15 | 12 | 17 |
| **Argentina** | 1,375 | low | 1 | 0.39 | 1.62 | 14 | 5 | 22 |
| **Australia** | 1,252 | low | 0.87 | 0.39 | 1.35 | 11 | 5 | 17 |
| **Colombia** | 767 | low | 1.2 | 0.27 | 2.13 | 9 | 2 | 16 |
| **Cuba** | 978 | low | 0.86 | 0.58 | 1.14 | 8 | 6 | 11 |
| **Netherlands** | 4,122 | low | 0.1 | 0 | 0.2 | 4 | 0 | 8 |
| **Austria** | 525 | low | 0.55 | 0.34 | 0.71 | 3 | 2 | 4 |
| **Sweden** | 837 | low | 0.2 | 0.1 | 0.4 | 2 | 1 | 3 |
| **Ireland** | 1,626 | low | 0.1 | 0 | 0.3 | 2 | 0 | 5 |

## Romania

| **Country** | **Population** | **HBsAg endemicity** | **HBsAg Prevalence** | | | **Estimated number of CHB cases** | | |
| --- | --- | --- | --- | --- | --- | --- | --- | --- |
|  |  |  | **%** | **LL 95%CI** | **UL 95%CI** | **Cases** | **Lower** | **Upper** |
| **Moldova** | 59,670 | int-high | 9.61 | 6.92 | 12.29 | 5,734 | 4,129 | 7,333 |
| **Bulgaria** | 11,163 | int-high | 4.25 | 2.8 | 5.7 | 474 | 313 | 636 |
| **China** | 2,978 | int-high | 10.23 | 9.35 | 11.11 | 305 | 278 | 331 |
| **Turkey** | 5,057 | int-high | 4.29 | 3.7 | 4.88 | 217 | 187 | 247 |
| **Ukraine** | 8,743 | int-high | 2.2 | 1.15 | 3.24 | 192 | 101 | 283 |
| **Russia** | 4,952 | int-high | 2.89 | 2.16 | 3.62 | 143 | 107 | 179 |
| **Syria** | 2,295 | int-high | 5.62 | 4.82 | 6.42 | 129 | 111 | 147 |
| **Greece** | 4,085 | int-high | 2.33 | 1.54 | 3.11 | 95 | 63 | 127 |
| **Tunisia** | 1,034 | int-high | 4.9 | 1.51 | 5.89 | 51 | 16 | 61 |
| **Serbia** | 1,529 | int-high | 3.29 | 2.33 | 4.24 | 50 | 36 | 65 |
| **Iran** | 1,114 | int-high | 3.1 | 2.69 | 3.5 | 35 | 30 | 39 |
| **Ghana** | 106 | int-high | 13.44 | 10.5 | 16.38 | 14 | 11 | 17 |
| **Mali** | 107 | int-high | 13.17 | 10.18 | 16.17 | 14 | 11 | 17 |
| **Nigeria** | 93 | int-high | 13.31 | 11.57 | 15.06 | 12 | 11 | 14 |
| **Guinea** | 73 | int-high | 16.33 | 14.61 | 18.05 | 12 | 11 | 13 |
| **Albania** | 102 | int-high | 9 | 8.1 | 9.8 | 9 | 8 | 10 |
| **Mauritania** | 48 | int-high | 13.17 | 10.18 | 16.17 | 6 | 5 | 8 |
| **Pakistan** | 149 | int-high | 4.17 | 3.59 | 4.75 | 6 | 5 | 7 |
| **Bangladesh** | 118 | int-high | 4.83 | 4.02 | 5.64 | 6 | 5 | 7 |
| **Equatorial Guinea** | 45 | int-high | 11.44 | 8.46 | 14.43 | 5 | 4 | 6 |
| **Senegal** | 37 | int-high | 12.66 | 10.14 | 15.18 | 5 | 4 | 6 |
| **Vietnam** | 31 | int-high | 12.48 | 11.46 | 13.5 | 4 | 4 | 4 |
| **Dominican Republic** | 35 | int-high | 10.68 | 5.89 | 15.46 | 4 | 2 | 5 |
| **Cameroon** | 27 | int-high | 11.44 | 8.46 | 14.43 | 3 | 2 | 4 |
| **Algeria** | 81 | int-high | 2.6 | 0 | 12.19 | 2 | 0 | 10 |
| **India** | 39 | int-high | 3.23 | 2.92 | 3.55 | 1 | 1 | 1 |
| **Egypt** | 29 | int-high | 4.18 | 1.85 | 6.51 | 1 | 1 | 2 |
| **Italy** | 22,486 | low | 1.89 | 1.26 | 2.52 | 425 | 283 | 567 |
| **Spain** | 18,827 | low | 0.66 | 0.34 | 0.97 | 124 | 64 | 183 |
| **Hungary** | 5,795 | low | 1.08 | 0.04 | 2.11 | 63 | 2 | 122 |
| **France** | 3,780 | low | 0.68 | 0.44 | 1.05 | 26 | 17 | 40 |
| **Germany** | 3,759 | low | 0.6 | 0.4 | 0.8 | 23 | 15 | 30 |
| **Israel** | 1,665 | low | 1.26 | 0.97 | 1.55 | 21 | 16 | 26 |
| **Iraq** | 1,136 | low | 1.31 | 0 | 2.87 | 15 | 0 | 33 |
| **United Kingdom** | 2,604 | low | 0.54 | 0.3 | 0.6 | 14 | 8 | 16 |
| **United States** | 2,360 | low | 0.27 | 0.2 | 0.34 | 6 | 5 | 8 |
| **Lebanon** | 118 | low | 1.66 | 0.86 | 2.46 | 2 | 1 | 3 |
| **Poland** | 89 | low | 1.44 | 1.16 | 1.72 | 1 | 1 | 2 |
| **Portugal** | 81 | low | 1.35 | 0.66 | 2.04 | 1 | 1 | 2 |
| **Morocco** | 38 | low | 1.8 | 1.51 | 5.89 | 1 | 1 | 2 |
| **Austria** | 121 | low | 0.55 | 0.34 | 0.71 | 1 | 0 | 1 |
| **Belgium** | 54 | low | 0.7 | 0.4 | 1.2 | 0 | 0 | 1 |
| **Cuba** | 39 | low | 0.86 | 0.58 | 1.14 | 0 | 0 | 0 |
| **Czech Republic** | 45 | low | 0.7 | 0.43 | 0.98 | 0 | 0 | 0 |
| **Denmark** | 47 | low | 0.55 | 0.34 | 0.71 | 0 | 0 | 0 |
| **Mexico** | 52 | low | 0.49 | 0.34 | 0.65 | 0 | 0 | 0 |
| **Sweden** | 54 | low | 0.2 | 0.1 | 0.4 | 0 | 0 | 0 |
| **Netherlands** | 83 | low | 0.1 | 0 | 0.2 | 0 | 0 | 0 |

## Slovakia

| **Country** | **Population** | **HBsAg endemicity** | **HBsAg Prevalence** | | | **Estimated number of CHB cases** | | |
| --- | --- | --- | --- | --- | --- | --- | --- | --- |
|  |  |  | **%** | **LL 95%CI** | **UL 95%CI** | **Cases** | **Lower** | **Upper** |
| **Romania** | 5,301 | int-high | 5.49 | 5.24 | 5.73 | 291 | 278 | 304 |
| **Ukraine** | 9,753 | int-high | 2.2 | 1.15 | 3.24 | 215 | 112 | 316 |
| **Vietnam** | 1,596 | int-high | 12.48 | 11.46 | 13.5 | 199 | 183 | 215 |
| **Russia** | 2,303 | int-high | 2.89 | 2.16 | 3.62 | 67 | 50 | 83 |
| **China** | 611 | int-high | 10.23 | 9.35 | 11.11 | 63 | 57 | 68 |
| **Bulgaria** | 1,303 | int-high | 4.25 | 2.8 | 5.7 | 55 | 36 | 74 |
| **Serbia** | 1,581 | int-high | 3.29 | 2.33 | 4.24 | 52 | 37 | 67 |
| **Chad** | 149 | int-high | 11.44 | 8.46 | 14.43 | 17 | 13 | 22 |
| **Afghanistan** | 126 | int-high | 10.46 | 5.85 | 15.07 | 13 | 7 | 19 |
| **South Korea** | 303 | int-high | 4.33 | 3.94 | 4.73 | 13 | 12 | 14 |
| **Bosnia and Herzegovina** | 329 | int-high | 3.63 | 2.26 | 5 | 12 | 7 | 16 |
| **Syria** | 174 | int-high | 5.62 | 4.82 | 6.42 | 10 | 8 | 11 |
| **Taiwan** | 82 | int-high | 10.23 | 9.35 | 11.11 | 8 | 8 | 9 |
| **Turkey** | 182 | int-high | 4.29 | 3.7 | 4.88 | 8 | 7 | 9 |
| **Kazakhstan** | 152 | int-high | 4.95 | 3.34 | 6.56 | 8 | 5 | 10 |
| **Moldova** | 73 | int-high | 9.61 | 6.92 | 12.29 | 7 | 5 | 9 |
| **Slovenia** | 208 | int-high | 3.29 | 2.33 | 4.24 | 7 | 5 | 9 |
| **Greece** | 240 | int-high | 2.33 | 1.54 | 3.11 | 6 | 4 | 7 |
| **Egypt** | 128 | int-high | 4.18 | 1.85 | 6.51 | 5 | 2 | 8 |
| **Belarus** | 163 | int-high | 3.19 | 1.72 | 4.65 | 5 | 3 | 8 |
| **Tunisia** | 85 | int-high | 4.9 | 1.51 | 5.89 | 4 | 1 | 5 |
| **India** | 100 | int-high | 3.23 | 2.92 | 3.55 | 3 | 3 | 4 |
| **Iran** | 74 | int-high | 3.1 | 2.69 | 3.5 | 2 | 2 | 3 |
| **Algeria** | 68 | int-high | 2.6 | 0 | 12.19 | 2 | 0 | 8 |
| **Lithuania** | 86 | int-high | 2.03 | 1.37 | 2.69 | 2 | 1 | 2 |
| **Czech Republic** | 86,378 | low | 0.7 | 0.43 | 0.98 | 605 | 371 | 847 |
| **Hungary** | 16,636 | low | 1.08 | 0.04 | 2.11 | 180 | 7 | 351 |
| **Poland** | 4,591 | low | 1.44 | 1.16 | 1.72 | 66 | 53 | 79 |
| **Italy** | 1,851 | low | 1.89 | 1.26 | 2.52 | 35 | 23 | 47 |
| **United Kingdom** | 4,943 | low | 0.54 | 0.3 | 0.6 | 27 | 15 | 30 |
| **Germany** | 3,041 | low | 0.6 | 0.4 | 0.8 | 18 | 12 | 24 |
| **France** | 2,329 | low | 0.68 | 0.44 | 1.05 | 16 | 10 | 24 |
| **Austria** | 2,607 | low | 0.55 | 0.34 | 0.71 | 14 | 9 | 19 |
| **Croatia** | 602 | low | 1.47 | 0.84 | 2.1 | 9 | 5 | 13 |
| **United States** | 2,338 | low | 0.27 | 0.2 | 0.34 | 6 | 5 | 8 |
| **Belgium** | 666 | low | 0.7 | 0.4 | 1.2 | 5 | 3 | 8 |
| **Spain** | 592 | low | 0.66 | 0.34 | 0.97 | 4 | 2 | 6 |
| **Switzerland** | 595 | low | 0.55 | 0.34 | 0.71 | 3 | 2 | 4 |
| **Canada** | 462 | low | 0.7 | 0.6 | 0.9 | 3 | 3 | 4 |
| **Australia** | 251 | low | 0.87 | 0.39 | 1.35 | 2 | 1 | 3 |
| **Israel** | 132 | low | 1.26 | 0.97 | 1.55 | 2 | 1 | 2 |
| **Norway** | 283 | low | 0.55 | 0.34 | 0.71 | 2 | 1 | 2 |
| **Portugal** | 82 | low | 1.35 | 0.66 | 2.04 | 1 | 1 | 2 |
| **Cuba** | 108 | low | 0.86 | 0.58 | 1.14 | 1 | 1 | 1 |
| **Ireland** | 814 | low | 0.1 | 0 | 0.3 | 1 | 0 | 2 |
| **Argentina** | 73 | low | 1 | 0.39 | 1.62 | 1 | 0 | 1 |
| **Denmark** | 116 | low | 0.55 | 0.34 | 0.71 | 1 | 0 | 1 |
| **Armenia** | 109 | low | 0.57 | 0.19 | 0.94 | 1 | 0 | 1 |
| **Netherlands** | 430 | low | 0.1 | 0 | 0.2 | 0 | 0 | 1 |
| **Sweden** | 147 | low | 0.2 | 0.1 | 0.4 | 0 | 0 | 1 |

## Slovenia

| **Country** | **Population** | **HBsAg endemicity** | **HBsAg Prevalence** | | | **Estimated number of CHB cases** | | |
| --- | --- | --- | --- | --- | --- | --- | --- | --- |
|  |  |  | **%** | **LL 95%CI** | **UL 95%CI** | **Cases** | **Lower** | **Upper** |
| **Bosnia and Herzegovina** | 98,527 | int-high | 3.63 | 2.26 | 5 | 3,577 | 2,227 | 4,926 |
| **Serbia** | 26,742 | int-high | 3.29 | 2.33 | 4.24 | 880 | 623 | 1,134 |
| **FYR Macedonia** | 14,730 | int-high | 3.29 | 2.33 | 4.24 | 485 | 343 | 625 |
| **Kosovo** | 10,414 | int-high | 3.29 | 2.33 | 4.24 | 343 | 243 | 442 |
| **Montenegro** | 2,834 | int-high | 3.29 | 2.33 | 4.24 | 93 | 66 | 120 |
| **China** | 839 | int-high | 10.23 | 9.35 | 11.11 | 86 | 78 | 93 |
| **Bulgaria** | 1,059 | int-high | 4.25 | 2.8 | 5.7 | 45 | 30 | 60 |
| **Russia** | 1,413 | int-high | 2.89 | 2.16 | 3.62 | 41 | 31 | 51 |
| **Ukraine** | 1,605 | int-high | 2.2 | 1.15 | 3.24 | 35 | 18 | 52 |
| **Moldova** | 327 | int-high | 9.61 | 6.92 | 12.29 | 31 | 23 | 40 |
| **Romania** | 361 | int-high | 5.49 | 5.24 | 5.73 | 20 | 19 | 21 |
| **Dominican Republic** | 165 | int-high | 10.68 | 5.89 | 15.46 | 18 | 10 | 26 |
| **Albania** | 142 | int-high | 9 | 8.1 | 9.8 | 13 | 12 | 14 |
| **Thailand** | 209 | int-high | 5.54 | 4.64 | 6.43 | 12 | 10 | 13 |
| **Philippines** | 91 | int-high | 7.36 | 6.32 | 8.39 | 7 | 6 | 8 |
| **Belarus** | 132 | int-high | 3.19 | 1.72 | 4.65 | 4 | 2 | 6 |
| **Jordan** | 61 | int-high | 6.36 | 3.72 | 9 | 4 | 2 | 5 |
| **Turkey** | 90 | int-high | 4.29 | 3.7 | 4.88 | 4 | 3 | 4 |
| **Egypt** | 92 | int-high | 4.18 | 1.85 | 6.51 | 4 | 2 | 6 |
| **India** | 117 | int-high | 3.23 | 2.92 | 3.55 | 4 | 3 | 4 |
| **Kazakhstan** | 54 | int-high | 4.95 | 3.34 | 6.56 | 3 | 2 | 4 |
| **Tunisia** | 51 | int-high | 4.9 | 1.51 | 5.89 | 2 | 1 | 3 |
| **Greece** | 96 | int-high | 2.33 | 1.54 | 3.11 | 2 | 1 | 3 |
| **Iran** | 69 | int-high | 3.1 | 2.69 | 3.5 | 2 | 2 | 2 |
| **Croatia** | 48,294 | low | 1.47 | 0.84 | 2.1 | 710 | 406 | 1,014 |
| **Italy** | 3,354 | low | 1.89 | 1.26 | 2.52 | 63 | 42 | 85 |
| **Germany** | 8,025 | low | 0.6 | 0.4 | 0.8 | 48 | 32 | 64 |
| **Austria** | 3,033 | low | 0.55 | 0.34 | 0.71 | 17 | 10 | 22 |
| **France** | 1,220 | low | 0.68 | 0.44 | 1.05 | 8 | 5 | 13 |
| **Switzerland** | 1,038 | low | 0.55 | 0.34 | 0.71 | 6 | 4 | 7 |
| **Poland** | 391 | low | 1.44 | 1.16 | 1.72 | 6 | 5 | 7 |
| **Hungary** | 404 | low | 1.08 | 0.04 | 2.11 | 4 | 0 | 9 |
| **Argentina** | 408 | low | 1 | 0.39 | 1.62 | 4 | 2 | 7 |
| **Slovakia** | 534 | low | 0.7 | 0.43 | 0.98 | 4 | 2 | 5 |
| **Australia** | 387 | low | 0.87 | 0.39 | 1.35 | 3 | 2 | 5 |
| **United Kingdom** | 587 | low | 0.54 | 0.3 | 0.6 | 3 | 2 | 4 |
| **Spain** | 447 | low | 0.66 | 0.34 | 0.97 | 3 | 2 | 4 |
| **Canada** | 414 | low | 0.7 | 0.6 | 0.9 | 3 | 2 | 4 |
| **Czech Republic** | 378 | low | 0.7 | 0.43 | 0.98 | 3 | 2 | 4 |
| **Belgium** | 313 | low | 0.7 | 0.4 | 1.2 | 2 | 1 | 4 |
| **United States** | 700 | low | 0.27 | 0.2 | 0.34 | 2 | 1 | 2 |
| **Brazil** | 102 | low | 1.78 | 1.54 | 2.02 | 2 | 2 | 2 |
| **Venezuela** | 49 | low | 1.93 | 1.13 | 2.73 | 1 | 1 | 1 |
| **Iraq** | 55 | low | 1.31 | 0 | 2.87 | 1 | 0 | 2 |
| **Sweden** | 355 | low | 0.2 | 0.1 | 0.4 | 1 | 0 | 1 |
| **Cuba** | 58 | low | 0.86 | 0.58 | 1.14 | 0 | 0 | 1 |
| **Japan** | 74 | low | 0.63 | 0.6 | 0.7 | 0 | 0 | 1 |
| **Netherlands** | 334 | low | 0.1 | 0 | 0.2 | 0 | 0 | 1 |
| **Denmark** | 50 | low | 0.55 | 0.34 | 0.71 | 0 | 0 | 0 |
| **Mexico** | 52 | low | 0.49 | 0.34 | 0.65 | 0 | 0 | 0 |

## Spain

| **Country** | **Population** | **HBsAg endemicity** | **HBsAg Prevalence** | | | **Estimated number of CHB cases** | | |
| --- | --- | --- | --- | --- | --- | --- | --- | --- |
|  |  |  | **%** | **LL 95%CI** | **UL 95%CI** | **Cases** | **Lower** | **Upper** |
| **Romania** | 715,033 | int-high | 5.49 | 5.24 | 5.73 | 39,255 | 37,468 | 40,971 |
| **China** | 160,460 | int-high | 10.23 | 9.35 | 11.11 | 16,415 | 15,003 | 17,827 |
| **Dominican Republic** | 152,947 | int-high | 10.68 | 5.89 | 15.46 | 16,335 | 9,009 | 23,646 |
| **Senegal** | 53,347 | int-high | 12.66 | 10.14 | 15.18 | 6,754 | 5,409 | 8,098 |
| **Bulgaria** | 140,046 | int-high | 4.25 | 2.8 | 5.7 | 5,952 | 3,921 | 7,983 |
| **Bolivia** | 174,288 | int-high | 3.03 | 0.08 | 5.98 | 5,281 | 139 | 10,422 |
| **Nigeria** | 33,970 | int-high | 13.31 | 11.57 | 15.06 | 4,521 | 3,930 | 5,116 |
| **Philippines** | 41,895 | int-high | 7.36 | 6.32 | 8.39 | 3,083 | 2,648 | 3,515 |
| **Pakistan** | 63,946 | int-high | 4.17 | 3.59 | 4.75 | 2,667 | 2,296 | 3,037 |
| **Mali** | 19,931 | int-high | 13.17 | 10.18 | 16.17 | 2,625 | 2,029 | 3,223 |
| **Equatorial Guinea** | 19,858 | int-high | 11.44 | 8.46 | 14.43 | 2,272 | 1,680 | 2,866 |
| **Russia** | 71,482 | int-high | 2.89 | 2.16 | 3.62 | 2,066 | 1,544 | 2,588 |
| **Ghana** | 13,238 | int-high | 13.44 | 10.5 | 16.38 | 1,779 | 1,390 | 2,168 |
| **Ukraine** | 80,755 | int-high | 2.2 | 1.15 | 3.24 | 1,777 | 929 | 2,616 |
| **Guinea** | 10,615 | int-high | 16.33 | 14.61 | 18.05 | 1,733 | 1,551 | 1,916 |
| **Moldova** | 16,986 | int-high | 9.61 | 6.92 | 12.29 | 1,632 | 1,175 | 2,088 |
| **Algeria** | 57,961 | int-high | 2.6 | 0 | 12.19 | 1,507 | 0 | 7,065 |
| **India** | 35,591 | int-high | 3.23 | 2.92 | 3.55 | 1,150 | 1,039 | 1,263 |
| **Gambia** | 18,103 | int-high | 3.41 | 2.4 | 4.46 | 617 | 434 | 807 |
| **Bangladesh** | 11,007 | int-high | 4.83 | 4.02 | 5.64 | 532 | 442 | 621 |
| **Lithuania** | 17,884 | int-high | 2.03 | 1.37 | 2.69 | 363 | 245 | 481 |
| **Morocco** | 740,097 | low | 1.8 | 1.51 | 5.89 | 13,322 | 11,175 | 43,592 |
| **Colombia** | 366,000 | low | 1.2 | 0.27 | 2.13 | 4,392 | 988 | 7,796 |
| **Peru** | 193,645 | low | 1.85 | 1.29 | 2.42 | 3,582 | 2,498 | 4,686 |
| **Venezuela** | 156,265 | low | 1.93 | 1.13 | 2.73 | 3,016 | 1,766 | 4,266 |
| **Argentina** | 264,031 | low | 1 | 0.39 | 1.62 | 2,640 | 1,030 | 4,277 |
| **Ecuador** | 452,396 | low | 0.47 | 0.42 | 0.51 | 2,126 | 1,900 | 2,307 |
| **Brazil** | 111,711 | low | 1.78 | 1.54 | 2.02 | 1,988 | 1,720 | 2,257 |
| **Italy** | 99,321 | low | 1.89 | 1.26 | 2.52 | 1,877 | 1,251 | 2,503 |
| **United Kingdom** | 321,138 | low | 0.54 | 0.3 | 0.6 | 1,734 | 963 | 1,927 |
| **Portugal** | 123,404 | low | 1.35 | 0.66 | 2.04 | 1,666 | 814 | 2,517 |
| **France** | 208,366 | low | 0.68 | 0.44 | 1.05 | 1,417 | 917 | 2,188 |
| **Germany** | 209,645 | low | 0.6 | 0.4 | 0.8 | 1,258 | 839 | 1,677 |
| **Cuba** | 124,013 | low | 0.86 | 0.58 | 1.14 | 1,067 | 719 | 1,414 |
| **Poland** | 68,707 | low | 1.44 | 1.16 | 1.72 | 989 | 797 | 1,182 |
| **Paraguay** | 71,854 | low | 0.78 | 0.52 | 1.05 | 560 | 374 | 754 |
| **Switzerland** | 63,097 | low | 0.55 | 0.34 | 0.71 | 347 | 215 | 448 |
| **Belgium** | 43,307 | low | 0.7 | 0.4 | 1.2 | 303 | 173 | 520 |
| **Uruguay** | 77,857 | low | 0.38 | 0.3 | 0.46 | 296 | 234 | 358 |
| **Mexico** | 49,166 | low | 0.49 | 0.34 | 0.65 | 241 | 167 | 320 |
| **Chile** | 59,714 | low | 0.4 | 0.11 | 0.69 | 239 | 66 | 412 |
| **Honduras** | 37,191 | low | 0.56 | 0.41 | 0.72 | 208 | 152 | 268 |
| **Nicaragua** | 19,029 | low | 0.68 | 0 | 1.78 | 129 | 0 | 339 |
| **United States** | 39,739 | low | 0.27 | 0.2 | 0.34 | 107 | 79 | 135 |
| **Norway** | 16,200 | low | 0.55 | 0.34 | 0.71 | 89 | 55 | 115 |
| **Denmark** | 11,500 | low | 0.55 | 0.34 | 0.71 | 63 | 39 | 82 |
| **Netherlands** | 47,610 | low | 0.1 | 0 | 0.2 | 48 | 0 | 95 |
| **Sweden** | 19,433 | low | 0.2 | 0.1 | 0.4 | 39 | 19 | 78 |
| **Finland** | 11,768 | low | 0.2 | 0.1 | 0.4 | 24 | 12 | 47 |
| **Ireland** | 14,623 | low | 0.1 | 0 | 0.3 | 15 | 0 | 44 |

## Sweden

| **Country** | **Population** | **HBsAg endemicity** | **HBsAg Prevalence** | | | **Estimated number of CHB cases** | | |
| --- | --- | --- | --- | --- | --- | --- | --- | --- |
|  |  |  | **%** | **LL 95%CI** | **UL 95%CI** | **CHB Cases** | **Lower** | **Upper** |
| **Somalia** | 43,966 | int-high | 12.4 | 8.89 | 15.92 | 5,452 | 3,909 | 6,999 |
| **China** | 27,422 | int-high | 10.23 | 9.35 | 11.11 | 2,805 | 2,564 | 3,047 |
| **Former Yugo-slavia (bf.1992)** | 69,269 | int-high | 3.98 | 1.32 | 6.64 | 2,757 | 914 | 4,599 |
| **Afghanistan** | 21,484 | int-high | 10.46 | 5.85 | 15.07 | 2,247 | 1,257 | 3,238 |
| **Eritrea** | 13,735 | int-high | 15.52 | 2.02 | 29.02 | 2,132 | 277 | 3,986 |
| **Bosnia and Herzegovina** | 56,595 | int-high | 3.63 | 2.26 | 5 | 2,054 | 1,279 | 2,830 |
| **Iran** | 65,649 | int-high | 3.1 | 2.69 | 3.5 | 2,035 | 1,766 | 2,298 |
| **Thailand** | 35,554 | int-high | 5.54 | 4.64 | 6.43 | 1,970 | 1,650 | 2,286 |
| **Vietnam** | 15,677 | int-high | 12.48 | 11.46 | 13.5 | 1,956 | 1,797 | 2,116 |
| **Turkey** | 45,085 | int-high | 4.29 | 3.7 | 4.88 | 1,934 | 1,668 | 2,200 |
| **Syria** | 27,510 | int-high | 5.62 | 4.82 | 6.42 | 1,546 | 1,326 | 1,766 |
| **Romania** | 22,079 | int-high | 5.49 | 5.24 | 5.73 | 1,212 | 1,157 | 1,265 |
| **Ethiopia** | 14,844 | int-high | 5.47 | 2.55 | 8.39 | 812 | 379 | 1,245 |
| **Philippines** | 10,981 | int-high | 7.36 | 6.32 | 8.39 | 808 | 694 | 921 |
| **India** | 19,415 | int-high | 3.23 | 2.92 | 3.55 | 627 | 567 | 689 |
| **Russia** | 17,320 | int-high | 2.89 | 2.16 | 3.62 | 501 | 374 | 627 |
| **South Korea** | 10,560 | int-high | 4.33 | 3.94 | 4.73 | 457 | 416 | 499 |
| **Pakistan** | 10,741 | int-high | 4.17 | 3.59 | 4.75 | 448 | 386 | 510 |
| **Bangladesh** | 6,716 | int-high | 4.83 | 4.02 | 5.64 | 324 | 270 | 379 |
| **Bulgaria** | 7,357 | int-high | 4.25 | 2.8 | 5.7 | 313 | 206 | 419 |
| **Greece** | 13,205 | int-high | 2.33 | 1.54 | 3.11 | 308 | 203 | 411 |
| **Serbia** | 7,274 | int-high | 3.29 | 2.33 | 4.24 | 239 | 169 | 308 |
| **Former Soviet Union** | 6,048 | int-high | 3.83 | 2.74 | 4.91 | 232 | 166 | 297 |
| **Former Yugoslav Republic** | 6,313 | int-high | 3.29 | 2.33 | 4.24 | 208 | 147 | 268 |
| **Lithuania** | 8,815 | int-high | 2.03 | 1.37 | 2.69 | 179 | 121 | 237 |
| **Sri Lanka** | 6,885 | int-high | 2.41 | 0 | 5.53 | 166 | 0 | 381 |
| **Ukraine** | 5,804 | int-high | 2.2 | 1.15 | 3.24 | 128 | 67 | 188 |
| **Iraq** | 127,860 | low | 1.31 | 0 | 2.87 | 1,675 | 0 | 3,670 |
| **Poland** | 75,323 | low | 1.44 | 1.16 | 1.72 | 1,085 | 874 | 1,296 |
| **Lebanon** | 24,743 | low | 1.66 | 0.86 | 2.46 | 411 | 213 | 609 |
| **Finland** | 163,867 | low | 0.2 | 0.1 | 0.4 | 328 | 164 | 655 |
| **Germany** | 48,731 | low | 0.6 | 0.4 | 0.8 | 292 | 195 | 390 |
| **Denmark** | 44,209 | low | 0.55 | 0.34 | 0.71 | 243 | 150 | 314 |
| **Norway** | 42,884 | low | 0.55 | 0.34 | 0.71 | 236 | 146 | 304 |
| **Hungary** | 15,678 | low | 1.08 | 0.04 | 2.11 | 169 | 6 | 331 |
| **Italy** | 8,636 | low | 1.89 | 1.26 | 2.52 | 163 | 109 | 218 |
| **Morocco** | 8,174 | low | 1.8 | 1.51 | 5.89 | 147 | 123 | 481 |
| **Colombia** | 11,131 | low | 1.2 | 0.27 | 2.13 | 134 | 30 | 237 |
| **Peru** | 7,127 | low | 1.85 | 1.29 | 2.42 | 132 | 92 | 172 |
| **UK** | 22,670 | low | 0.54 | 0.3 | 0.6 | 122 | 68 | 136 |
| **Brazil** | 6,516 | low | 1.78 | 1.54 | 2.02 | 116 | 100 | 132 |
| **Chile** | 28,425 | low | 0.4 | 0.11 | 0.69 | 114 | 31 | 196 |
| **Croatia** | 6,422 | low | 1.47 | 0.84 | 2.1 | 94 | 54 | 135 |
| **Estonia** | 10,145 | low | 0.58 | 0.42 | 0.74 | 59 | 43 | 75 |
| **France** | 8,385 | low | 0.68 | 0.44 | 1.05 | 57 | 37 | 88 |
| **Spain** | 7,708 | low | 0.66 | 0.34 | 0.97 | 51 | 26 | 75 |
| **United States** | 18,312 | low | 0.27 | 0.2 | 0.34 | 49 | 37 | 62 |
| **Former Czechoslovakia** | 5,692 | low | 0.7 | 0.43 | 0.98 | 40 | 24 | 56 |
| **Austria** | 5,788 | low | 0.55 | 0.34 | 0.71 | 32 | 20 | 41 |
| **Netherlands** | 9,401 | low | 0.1 | 0 | 0.2 | 9 | 0 | 19 |

## United Kingdom

| **Country** | **Population** | **HBsAg endemicity** | **HBsAg Prevalence** | | | **Estimated number of CHB cases** | | |
| --- | --- | --- | --- | --- | --- | --- | --- | --- |
|  |  |  | **%** | **LL 95%CI** | **UL 95%CI** | **Cases** | **Lower** | **Upper** |
| **China** | 284,070 | int-high | 10.23 | 9.35 | 11.11 | 29,060 | 26,561 | 31,560 |
| **Nigeria** | 201,185 | int-high | 13.31 | 11.57 | 15.06 | 26,778 | 23,277 | 30,298 |
| **India** | 722,435 | int-high | 3.23 | 2.92 | 3.55 | 23,335 | 21,095 | 25,646 |
| **Pakistan** | 502,795 | int-high | 4.17 | 3.59 | 4.75 | 20,967 | 18,050 | 23,883 |
| **Zimbabwe** | 123,670 | int-high | 13.91 | 10.7 | 17.11 | 17,202 | 13,233 | 21,160 |
| **Ghana** | 95,665 | int-high | 13.44 | 10.5 | 16.38 | 12,857 | 10,045 | 15,670 |
| **Somalia** | 103,050 | int-high | 12.4 | 8.89 | 15.92 | 12,778 | 9,161 | 16,406 |
| **South Africa** | 203,475 | int-high | 6.2 | 4.68 | 7.71 | 12,615 | 9,523 | 15,688 |
| **Bangladesh** | 214,090 | int-high | 4.83 | 4.02 | 5.64 | 10,341 | 8,606 | 12,075 |
| **Philippines** | 129,835 | int-high | 7.36 | 6.32 | 8.39 | 9,556 | 8,206 | 10,893 |
| **Kenya** | 140,535 | int-high | 5.7 | 4.21 | 7.2 | 8,010 | 5,917 | 10,119 |
| **Afghanistan** | 63,495 | int-high | 10.46 | 5.85 | 15.07 | 6,642 | 3,714 | 9,569 |
| **Jamaica** | 160,775 | int-high | 3.94 | 0.81 | 7.07 | 6,335 | 1,302 | 11,367 |
| **Uganda** | 60,885 | int-high | 10.27 | 8.54 | 11.99 | 6,253 | 5,200 | 7,300 |
| **Romania** | 83,170 | int-high | 5.49 | 5.24 | 5.73 | 4,566 | 4,358 | 4,766 |
| **Turkey** | 93,915 | int-high | 4.29 | 3.7 | 4.88 | 4,029 | 3,475 | 4,583 |
| **Mauritius** | 41,980 | int-high | 9.32 | 8.15 | 10.48 | 3,913 | 3,421 | 4,400 |
| **Malaysia** | 69,940 | int-high | 5.58 | 4.27 | 6.88 | 3,903 | 2,986 | 4,812 |
| **Vietnam** | 30,170 | int-high | 12.48 | 11.46 | 13.5 | 3,765 | 3,457 | 4,073 |
| **Sri Lanka** | 129,075 | int-high | 2.41 | 0 | 5.53 | 3,111 | 0 | 7,138 |
| **Iran** | 84,735 | int-high | 3.1 | 2.69 | 3.5 | 2,627 | 2,279 | 2,966 |
| **Singapore** | 42,190 | int-high | 6.02 | 5.03 | 7.01 | 2,540 | 2,122 | 2,958 |
| **Thailand** | 44,085 | int-high | 5.54 | 4.64 | 6.43 | 2,442 | 2,046 | 2,835 |
| **Lithuania** | 108,710 | int-high | 2.03 | 1.37 | 2.69 | 2,207 | 1,489 | 2,924 |
| **Bulgaria** | 48,260 | int-high | 4.25 | 2.8 | 5.7 | 2,051 | 1,351 | 2,751 |
| **Tanzania** | 35,995 | int-high | 5.65 | 4.43 | 6.86 | 2,034 | 1,595 | 2,469 |
| **Egypt** | 31,340 | int-high | 4.18 | 1.85 | 6.51 | 1,310 | 580 | 2,040 |
| **Nepal** | 50,880 | int-high | 2.32 | 1.71 | 2.93 | 1,180 | 870 | 1,491 |
| **Russian Federation** | 39,530 | int-high | 2.89 | 2.16 | 3.62 | 1,142 | 854 | 1,431 |
| **Greece** | 36,935 | int-high | 2.33 | 1.54 | 3.11 | 861 | 569 | 1,149 |
| **Poland** | 654,010 | low | 1.44 | 1.16 | 1.72 | 9,418 | 7,587 | 11,249 |
| **Italy** | 141,205 | low | 1.89 | 1.26 | 2.52 | 2,669 | 1,779 | 3,558 |
| **Germany** | 299,745 | low | 0.6 | 0.4 | 0.8 | 1,798 | 1,199 | 2,398 |
| **Portugal** | 92,065 | low | 1.35 | 0.66 | 2.04 | 1,243 | 608 | 1,878 |
| **Australia** | 126,315 | low | 0.87 | 0.39 | 1.35 | 1,099 | 493 | 1,705 |
| **Iraq** | 75,295 | low | 1.31 | 0 | 2.87 | 986 | 0 | 2,161 |
| **France** | 139,420 | low | 0.68 | 0.44 | 1.05 | 948 | 613 | 1,464 |
| **Brazil** | 52,150 | low | 1.78 | 1.54 | 2.02 | 928 | 803 | 1,053 |
| **Latvia** | 61,440 | low | 1.39 | 1.1 | 1.67 | 854 | 676 | 1,026 |
| **Cyprus** | 78,900 | low | 0.9 | 0.3 | 2 | 710 | 237 | 1,578 |
| **Hungary** | 52,250 | low | 1.08 | 0.04 | 2.11 | 564 | 21 | 1,102 |
| **Canada** | 80,390 | low | 0.7 | 0.6 | 0.9 | 563 | 482 | 724 |
| **Spain** | 84,820 | low | 0.66 | 0.34 | 0.97 | 560 | 288 | 823 |
| **United States of America** | 197,355 | low | 0.27 | 0.2 | 0.34 | 533 | 395 | 671 |
| **Ireland** | 468,185 | low | 0.1 | 0 | 0.3 | 468 | 0 | 1,405 |
| **Slovakia** | 63,055 | low | 0.7 | 0.43 | 0.98 | 441 | 271 | 618 |
| **New Zealand** | 62,585 | low | 0.5 | 0.42 | 0.56 | 313 | 263 | 350 |
| **Czech Republic** | 38,705 | low | 0.7 | 0.43 | 0.98 | 271 | 166 | 379 |
| **Japan** | 37,330 | low | 0.63 | 0.6 | 0.7 | 235 | 224 | 261 |
| **Netherlands** | 63,715 | low | 0.1 | 0 | 0.2 | 64 | 0 | 127 |
